# Cytochrome B5 type A alleviates HCC metastasis via regulating STOML2 related autophagy and promoting sensitivity to ruxolitinib

**DOI:** 10.1038/s41419-022-05053-8

**Published:** 2022-07-18

**Authors:** Hongrui Guo, Shuhang Liang, Yan Wang, Shuo Zhou, Dalong Yin, Shugeng Zhang, Jizhou Wang, Dehai Wu, Kun Ma, Yufeng Liu, Linmao Sun, Changyong Ji, Xianying Li, Huanran Zhou, Guangchao Yang, Xinyu Guo, Tianming Cui, Zihao Li, Yao Liu, Jiabei Wang, Lianxin Liu

**Affiliations:** 1 Department of Hepatic Surgery, Key Laboratory of Hepatosplenic Surgery, Ministry of Education, The First Affiliated Hospital of Harbin Medical University, Harbin, China; 2 Department of Hepatobiliary Surgery, Anhui Province Key Laboratory of Hepatopancreatobiliary Surgery, The First Affiliated Hospital of USTC, Division of Life Sciences and Medicine, University of Science and Technology of China, Hefei, Anhui 230001 China; 3 Department of Colorectal Surgery, Harbin Medical University Cancer Hospital, Harbin, 150001 Heilongjiang China

**Keywords:** Gastrointestinal cancer, Metastasis

## Abstract

The incidence of hepatocellular carcinoma (HCC) is increasing in the world. However, its role and underlying molecular mechanism in HCC progression remain unclear. We found that CYB5A plays a key role in HCC metastasis by inhibiting the JAK1/STAT3 pathway through binding to STOML2. CYB5A combined with STOML2 can predict the outcome of patients. To demonstrate the effect of CYB5A on JAK1 inhibitor function, we applied Ruxolitinib in metastatic tumors with high CYB5A expression and found that it slowed disease progression and prolonged survival in mice. To the best of our knowledge, this study is the first to report the Ruxolitinib effect on the metastatic ability of HCC cells in vivo and in vitro.

## Introduction

Hepatic blood flow is abundant, and hepatocellular carcinoma (HCC) mainly metastases through blood. HCC cells gradually infiltrate into adjacent blood vessels, forming adjacent microvascular invasion, follow by forming macrovascular invasion, and finally, intrahepatic metastasis, which is also the most important site for HCC metastasis. HCC cells can also enter the blood system and travel with the blood to distant organs, most commonly the lungs, followed by the bones and brain, and eventually lead to a patient’s death from multiple organ dysfunction syndrome. Therefore, HCC treatment needs to find the key molecules and corresponding signaling pathways that affect metastasis.

Cytochrome B5 Type A (CYB5A) is also known as Cytochrome B5, Cyb5, MCB5. The protein encoded by this gene binds to the membrane that is mainly expressed in the liver. CYB5A binds iron porphyrin elements acting as an electron carrier for membrane-bound oxygenases to reduce hemoglobin into the ferrous hemoglobin required for stearyl coenzyme desaturase activity [1]. Defects in this gene are one of the causes of inherited hereditary methemoglobinemia [2, 3]. In fact, CYB5A is associated with the initiation and progression of tumors. In various cancer types, such as prostate cancer and adrenal cortical cancer, scholars found that CYB5A is significantly downregulated in cancer compared with the corresponding adjacent normal tissues through statistical analysis without subjective impression [4–6]. This seems to suggest that CYB5A may be a tumor-suppressor gene in pan-cancer. Unfortunately, the antitumor effect of CYB5A in pancreatic cancer has only been briefly discussed so far [7, 8]. The CYB5A role in HCC and the underlying mechanism of CYB5A in inhibiting tumors have not been explored.

Signal Transducer and Activator of Transcription 3 (STAT3) signaling pathway have been reported in many inflammations and tumors [9]. IL-6, or interleukin-6, binds to a cell surface receptor and autophosphorylates Janus kinase (JAK) in the cytoplasmic region of gp130 and then phosphorylates STAT3 and activates it [10]. The activated STAT3 binds in pairings and enters the nucleus as a transcription factor, where it binds to specific sites on the chromosome and thus initiating the expression of related genes. Studies have shown that STAT3 can promote the expression of B cell lymphoma protein-2 (BCL2), Matrix metalloproteinase-9 (MMP-9), Hypoxia Inducible Factor 1 Subunit Alpha (HIF1A), and so on [11–15]. In addition, many researchers have found that STAT3 activation can act as a proto-oncogene by inhibiting autophagy activity in many other tumors [16–19].

Here, we demonstrated that reduced CYB5A expression levels are associated with HCC progression and poor prognosis. Silencing CYB5A enhances the stability of Stomatin Like 2 (STOML2), which stimulates STAT3 phosphorylation. Moreover, JAK inhibitor Ruxolitinib’s application in metastatic tumors with high CYB5A expression inhibited disease progression and prolonged survival in mice. Overall, our study demonstrates a novel signaling pathway that mediates HCC progression produced by CYB5A.

## Materials and methods

### Duolink® PLA

Before starting, the samples should be deposited on glass slides and cells were fixed, using PBS to wash cells three times, disposing of cells with 0.25%Triton-X100 for 5 min, then washing cells with PBST solution. Following the steps of Duolink® PLA Fluorescence Protocol(sigma) to test. Using the fluorescence or confocal microscope to analyze the images.

### Confocal microscope

HCC cells were plated in confocal culture dishes. MRFP-GFP-LC3 adenoviral vectors were purchased from HanBio Technology. LC3 puncta were examined with Zeiss LSM800 confocal microscope (Carl Zeiss).

Other Materials and Methods are described in S Materials and Methods.

## Results

### CYB5A is frequently downregulated in human HCC

**Fig. 1:**
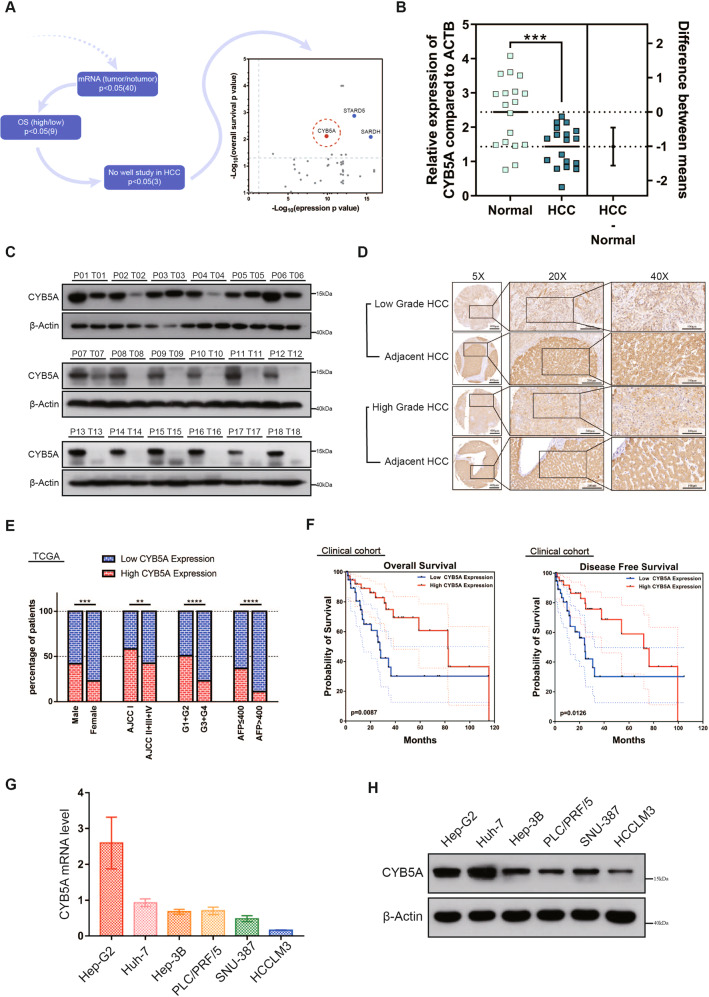
CYB5A is frequently downregulated in human HCC. **A** Workflow for selecting prognostic proteins with dots showing the 42 candidate proteins. **B** CYB5A mRNA level was analyzed in 18 HCC and paracancerous tissue specimens using real-time quantitative reverse transcription-polymerase chain reaction (qRT-PCR). **C**, **D** Western blot and immunohistochemical (IHC) analyses of CYB5A protein expression in HCC tissues and adjacent normal tissues. T: tumor; P: normal paracancerous tissue. **E** Low CYB5A expression was associated with poor clinicopathological features, including gender, AJCC stage (I and II–IV), pathological grade (G1, G2, or G3–G4), and serum AFP (<400 μg/L or ≥400 μg/L) in TCGA cohort (*n* = 360). *p < 0.05, **p < 0.01, ***p < 0.001, ****p < 0.0001; ns, not significant. **F** A Kaplan–Meier analysis of overall survival (OS) and disease-free survival (DFS) in a clinical cohort (*n* = 80) with different CYB5A expressions. **G**, **H** Relative CYB5A levels in six HCC cell lines were analyzed by qRT-PCR and western blot. Data are means ± SD of three independent experiments.

To study the differentially expressed genes in HCC patients, We referred to the QiangGao report that studied the protein expression levels of patients with HBV-related HCC [20]. We performed a supervised analysis to identify robust and representative prognostic proteins (Fig. 1A). Three downregulated proteins that mainly converged on amino acid metabolism and oxidoreductase activity were identified after stringent filtering (Supplementary data 1). This article is devoted to studying CYB5A, one of the three genes. The other two genes, SARDH and STARD5, were studied in our other topics.

From HCCDB data, an integrative molecular database of HCC, we know that CYB5A expression is the highest in normal human liver tissues, compared with other normal tissues (Supplementary fig. 1-1A), and it decreased significantly in HCC tissues (Supplementary fig. 1-1B). qRT-PCR results manifested that CYB5A mRNA was decreased in human HCC tissues (*n* = 18) compared with that in normal liver samples *p* < 0.05 (Fig. 1B), and we obtained the same conclusion from RNA sequencing results of 7 GEO databases (Supplementary fig. 1-1C). Besides, CYB5A protein was expressed at a significantly lower level in HCC samples by western blot analysis (Fig. 1C, Supplementary fig. 1-1D), and IHC (Fig. 1D). Moreover, TCGA clinicopathological characteristic analysis indicated that CYB5A expression is negatively associated with AJCC stage (*p* < 0.01), pathological grade (*p* < 0.0001), and serum AFP (*p* < 0.0001), and is related to gender (*p* < 0.001) (Fig. 1E and Supplementary data 2). Our data reveal that CYB5A is pathologically associated with cancer recurrence and patient outcome. The Kaplan-Meier curves showed that patients in low CYB5A expression group exhibited a decreased trend in overall survival (OS) and disease-free survival (DFS) than high CYB5A expression group (Fig. 1F), consistent with TCGA, GEO(GSE14520) and HCCDB dataset analysis (Supplementary figs. 1–2A–F). Unfortunately, univariate and multivariate Cox regression analyses in Clinical Cohort and TCGA Cohort revealed that CYB5A was not an independent predictor of HCC aggressiveness (Supplementary figs. 1–2G–J). CYB5A mRNA level and protein levels decreased with the increasing malignant degree of HCC cell lines (Fig. 1G, H). As widely used normal human liver cell lines (e.g., LO2, Chang liver, and WRL-68) are reported as Hela contaminants [21–23], we have not confirmed their relationship to HCC cell lines.

These results indicated that CYB5A is frequently downregulated in HCC patients and may act as a predictor for HCC survival and recurrence.

### CYB5A inhibits HCC metastasis

In order to further clarify the function of CYB5A in HCC, we first need to explore whether there are mutations in patients. However, we found that CYB5A is rarely mutated, by mining the TCGA database (only 1 of 360 HCC patients) (Supplementary data 3). And there are almost no pathogenic reports of human CYB5A gene mutation. Therefore, the effect of CYB5A gene mutation on the differential expression of HCC was not considered.

Then, we selected two cell lines with high expression of CYB5A (Huh-7 and Hep-G2) and two HCC cell lines with low expression of CYB5A (HCCLM3 and SNU-387). CYB5A was knocked down or overexpressed in the corresponding cell lines by lentivirus transfection shRNA and 3xflag-CYB5A, respectively, and the gene expression was confirmed by immunoblot and qRT-PCR (Supplementary figs. 2–1A–D). Simultaneously, we surprisingly found that CYB5A may affect the expression of some commonly used loading control, such as GAPDH and α/β-Tublin. So, we chose β-Actin as a loading control (Supplementary fig. 2–1E).

**Fig. 2:**
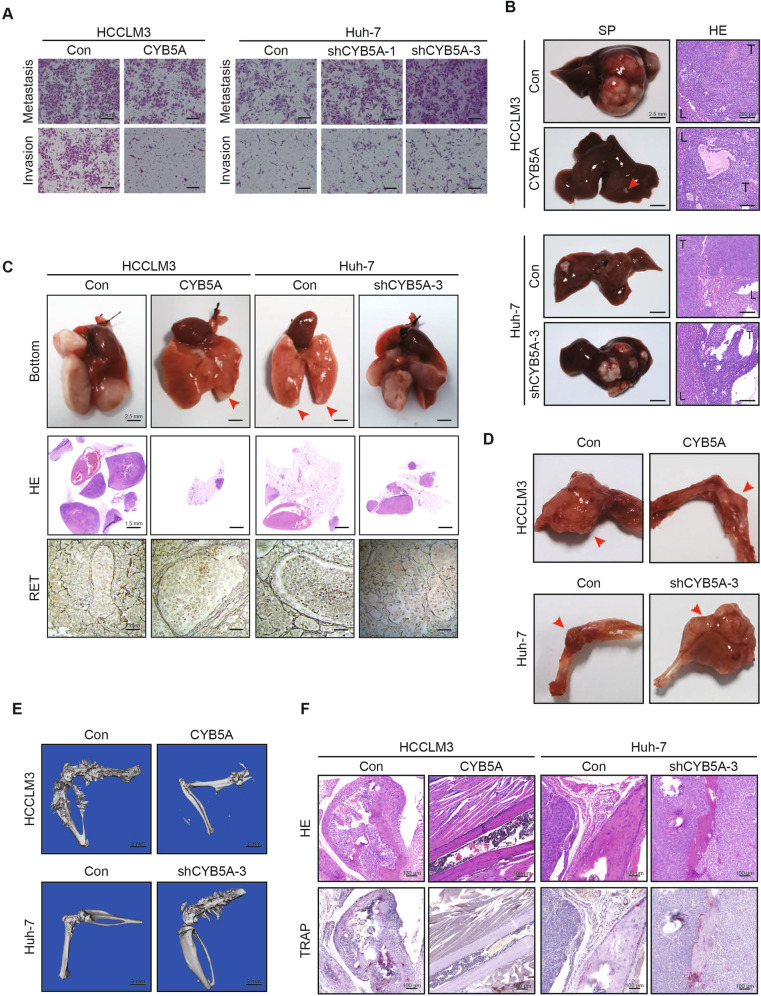
CYB5A inhibits metastasis of HCC. **A** Invasion and migration transwell assays for indicated cell lines. Scale bar: 50 μm. **B** Representative images of intrahepatic metastases (*n* = 10). SP: specimen, HE: Hematoxylin-eosin staining. T: tumor, L: normal liver. **C** Representative images of pulmonary metastases (*n* = 10). Damaged reticular fibers indicate tumor metastasis. RET: reticular fiber staining. **D**–**F** Representative images of bone or brain metastases (*n* = 10). The areas of overt osteolysis were tracked by micro-computed tomography (Micro-CT) analysis. Osteoclasts during osteolysis of the tumor were shown by tartrate-resistant acid phosphatase (TRAP) staining.

Wound-healing assays showed that CYB5A overexpression reduced SNU-387 and HCCLM3 migration whereas CYB5A knockdown accelerated Hep-G2 and Huh-7 migration (Supplementary fig. 2-2A). Matrigel-uncoated (migration) and -coated (invasion) transwell assays showed that CYB5A upregulation in SNU-387 and HCCLM3 significantly reduced cell migration and invasion whereas CYB5A downregulation in Hep-G2 and Huh-7 enhanced cell migration and invasion (Fig. 2A, Supplementary fig. 2-2B). In order to reduce the workload, Lv-shCYB5A-3 was only used in most of the subsequent knockdown experiments, and the group was labeled as shCYB5A-3 or shCYB5A.

The hepatic metastatic models of HCC cells injected through the spleen demonstrated that CYB5A knockdown increased the number of metastatic nodules while CYB5A overexpression reduced it (Fig. 2B). We evaluated CYB5A’s role in tumor metastasis by injecting HCC cells into nude mice via their tail veins and monitoring lung metastatic nodules. CYB5A repression increased lung metastasis incidence and metastatic nodules’ number whereas CYB5A induction decreased them (Fig. 2C, Supplementary fig. 2-2C, D). Through reticular fiber (RET) staining of the lung tumor tissues, it was found that RET was more complete in tissues with relatively high expression of CYB5A. Besides, we injected HCC cells into nude mice via their left ventricle. After waiting for four weeks, we observed that cell lines with relatively low expression of CYB5A had less bone and brain metastatic nodule (Fig. 2D, Supplementary fig. 2-2E). Furthermore, while proving bone metastasis, micro-computed tomography (Micro-CT) (Fig. 2E) and tartrate-resistant acid phosphatase (TRAP) staining showed that cell lines that knockdown CYB5A caused stronger osteolytic destruction of cortical bone and that CYB5A overexpression would weaken it (Fig. 2F). In short, we proved the inhibitory effect of CYB5A on the HCC metastasis in vivo and in vitro. The relevant statistics are shown in the supplemental figs. (Supplementary figs. 2–3A–G).

Overexpression or knockdown of CYB5A had no significant effect on the proliferation of HCC lines HCCLM3 or Huh-7 as measured using CCK8 proliferation analysis (Supplementary figs. 2–4A, B). Similar to its effects on cell proliferation, no significant difference was found in colonies’ number in colony formation assays (Supplementary figs. 2–4C, D). Therefore, we think that CYB5A’s effect on HCC proliferation is not obvious.

### The inhibition of metastasis by CYB5A is related to its ability to increase autophagy

**Fig. 3:**
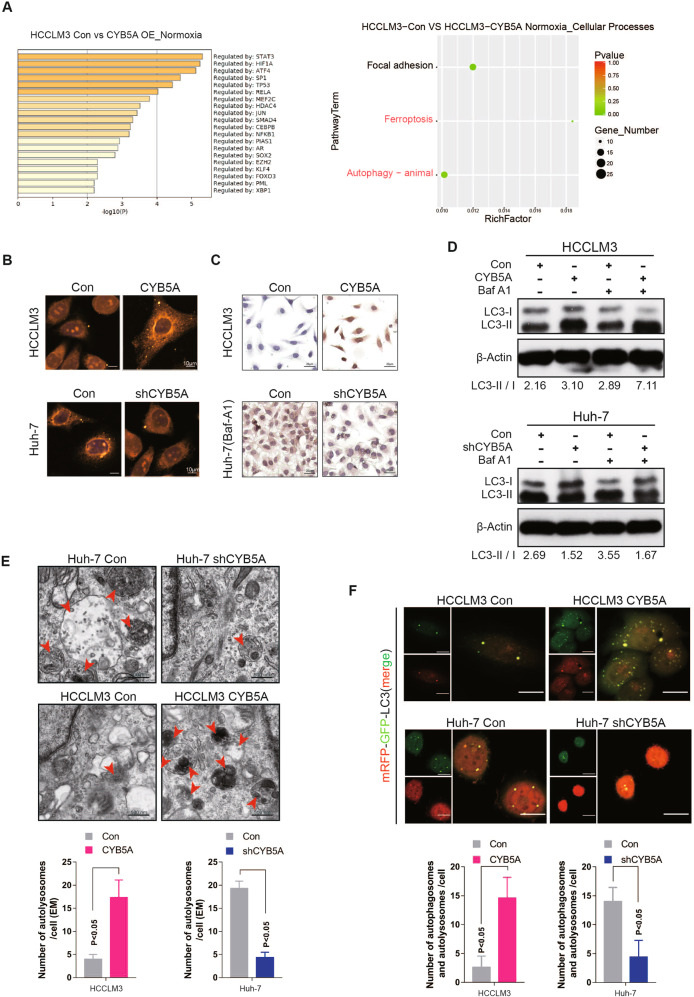
The inhibition of metastasis by CYB5A is related to its ability to increase autophagy. **A** The different expression genes under normoxia between HCCLM3-control and HCCLM3-CYB5A cell lines. The significant thresholds were |fold change | > 2 and *p* < 0.05. Significant different expression genes were filtered and enriched in GO TRRUST analysis and KEGG signaling pathway. **B**, **C** After the cells were fixed by paraformaldehyde, LC3 expression was detected by immunofluorescence (IF) and immunocytochemistry (ICC). **D** Cell lines treated with or without 100 nM bafilomycin A1 (Baf A1) for 4 h. Cell lysates were analyzed by western blotting. **E** Images represented the typical transmission electron microscopy of indicated cell lines in the top panel and statistical analysis of autophagolysosome (ASS) numbers are in the bottom panel. The red arrows indicate ASS. **F** Images represented the typically indicated cell lines that stably expressed mRFP-EGFP-LC3 fusion protein which was shown by confocal microscopic analysis in the top panel and statistical analysis of autophagosome and ASS numbers in the bottom panel. Scale bar: 20 μm.

In order to explain the phenomenon that CYB5A can inhibit HCC metastasis, mRNA sequencing analysis was performed. The differential expression genes (|fold change| > 2, *p* < 0.05) were filtered (Supplementary data 4). After gene enrichment analysis, STAT3 and Hypoxia Inducible Factor 1 Subunit Alpha (HIF1A) are at the top of the list. This suggests that CYB5A may affect the state of cells under hypoxia. Similarly, we sequenced mRNA under the condition of hypoxia. Combined with the two analyses’ results, the enrichment of differential genes is most concentrated on “Cellular Processes”, and most of the sub-pathways are related to autophagy (Fig. 3A, Supplementary figs. 3–1 A–C). Autophagy is known to decrease the metastatic ability of tumor cells [24–26]. The metastatic ability decreased upon rapamycin (200 nM, 24 h) treatment in HCC cell lines (Supplementary figs. 3-2A, B).

To detect the occurrence of autophagy after CYB5A overexpression or knockdown, we conducted immunocytochemistry (ICC), immunofluorescence (IF), and western blot analysis to examine the levels of autophagy-related protein: LC-3B. As shown the expression levels of LC3-II/I proteins were increased in CYB5A-overexpressing HCC cells and decreased in CYB5A-knockdown HCC cells (Fig. 3B, C). Since the increased LC3-II/I may be due to either autophagy induction or inhibition of autophagic flux, Bafilomycin A1 (BafA1), an inhibitor of fusion between autophagosomes and lysosomes [27], was used in this study. The BafA1-treated negative control cells displayed increased accumulation of LC3-II, and the ectopic expression of CYB5A may enhance these effects (Fig. 3D). Taken together, our data indicated that inductive effects of CYB5A on autophagy could be resulted from early induction stages of autophagy rather than from autophagosome degradation suppression. Furthermore, autophagosomes were evaluated in HCCLM3, SNU-387, Huh-7, and Hep-G2 cells with or without CYB5A expression regulation exposed to 20 h of hypoxia (5% oxygen). Transmission electron microscopy showed an increase in the formation of autophagic vesicles in cells with relatively high expression of CYB5A (Fig. 3E, Supplementary fig. 3–1D, E). In addition, we established HCCLM3, SNU-387, Huh-7, and Hep-G2 cells that stably expressed a tandem mRFP-EGFP-LC3 construct (Fig. 3F, Supplementary fig. 3–1F, G). We found that CYB5A induced the autophagic flux in cells with relatively high expression of CYB5A. As mentioned above CYB5A expression was correlated with HIF1A. Besides, hypoxia is an important way to activate autophagy [24, 26, 28]. Consequently, we further investigated the relationship between them. Analysis of TCGA database suggested a negative correlation between CYB5A and HIF1A (Supplementary fig. 3-3A–D); The cell status of HCCLM3 cell lines overexpressing CYB5A was significantly worse than that of the control group under hypoxia for 24 h, in contrast, the cell status of Huh-7 cell line of CYB5A was knocked down was significantly better than that of the control group (Supplementary fig. 3-3E). The apoptosis rate under hypoxia was increased by CYB5A overexpression, and decreased by CYB5A inhibition (Supplementary fig. 3-3F–H). These suggested that CYB5A further inhibited tumor progression in hypoxia.

Collectively, our data indicate that CYB5A inhibits HCC cell metastasis through autophagy pathway.

### CYB5A promotes autophagy by inhibiting the STAT3 signaling pathway

**Fig. 4:**
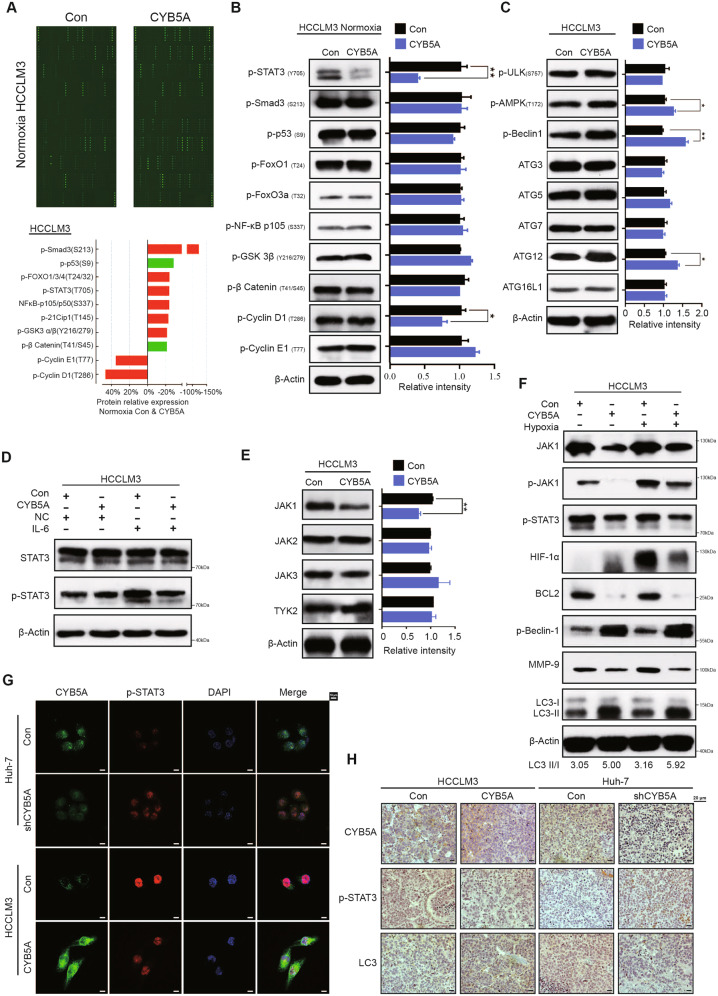
CYB5A promotes autophagy by inhibiting the STAT3 signaling pathway. **A** The image of phosphorylated protein content determination array (top panel). Core differential proteins were screened by |fold change| > 1.2 of the phosphorylation Ab array (bottom panel). **B** Western blotting showed that p-STAT3 was the most differentially expressed protein. **C** Western blot was performed to detect autophagy-related protein expression in HCCLM3 cells. The relative density of each band was analyzed by ImageJ software. **D** Indicated Cells were exposed to IL-6 (20 ng/mL, 24 h). Whole-cell lysates were analyzed by Western blotting. **E** Western blot was performed to detect JAK1, JAK2, JAK3, and TYK2 expression in the indicated cells. **F** Indicated cells were exposed to Hypoxia (20 h). Whole-cell lysates were analyzed by Western blotting. **G** Typical IF images of CYB5A and p-STAT3 expression in the indicated HCC cell lines. **H** p-STAT3, LC-3B, and CYB5A levels in lung xenograft tumors were shown by IHC. To prove that CYB5A inhibits STAT3 rather than parallelism, we performed rescue experiments in vitro and in vivo (Supplementary figs. 4–1F and 4–2).

Because mRNA level may not affect the corresponding protein’s activation state, we performed phosphorylated protein content determination microarray. And the typical differences of phosphorylated proteins were selected (Fig. 4A, Supplementary fig. 4–1A, Supplementary data 5) and tested by western blot analysis (Fig. 4B, Supplementary fig. 4–1B). Coincidentally, the results showed that p-STAT3(Y705) was the most differentiated phosphorylated protein.

To further confirm that CYB5A does play a role in this autophagy pathway rather than in other pathways, we measured the relative density of autophagy-related proteins (Fig. 4C, Supplementary fig. 4–1C). As expected, p-Beclin-1 exhibited the biggest difference. (Several articles have reported that JAK1/STAT3 pathway is upstream of the autophagy star protein p-Beclin-1 [29]). Further, HCC cells exposed to IL-6 (20 ng/mL, 24 h) showed p-STAT3(Y705) concentration had a more obvious diversity, but STAT3 concentration had no significant difference (Fig. 4D, Supplementary fig. 4–1D). This implies that CYB5A may affect kinase expression in STAT3. To examine this possibility, we measured the expression of the four most important Janus kinases (JAK1, JAK2, JAK3, and TYK2) upstream of STAT3 [30, 31]. The JAK1 expression was significantly negatively regulated by CYB5A (Fig. 4E, Supplementary fig. 4–1E). Differences in relative density of proteins in the related pathways demonstrated by western blotting analysis, JAK1, p-JAK1, p-STAT3, BCL2, p-Beclin-1, MMP-9, and LC3 had a trend of change consistent with expectations (Fig. 4F, Supplementary fig. 4–1F). The purpose of hypoxia is to induce stronger autophagy.

Immunofluorescence (IF) showed the difference in relative concentrations of CYB5A and p-STAT3 (Fig. 4G) and IHC of lung xenograft tumors, demonstrating a similar trend (Fig. 4H).

To prove that CYB5A inhibits STAT3 rather than parallelism, we performed rescue experiments in vitro and in vivo (Supplementary figs. 4–1G and 4–2). In conclusion, we firmly believe that CYB5A induces autophagy by inhibiting STAT3 pathway as clearly sorted out in Supplementary fig. 4–1H.

### CYB5A inhibits the STAT3 signaling pathway by inhibiting STOML2

**Fig. 5:**
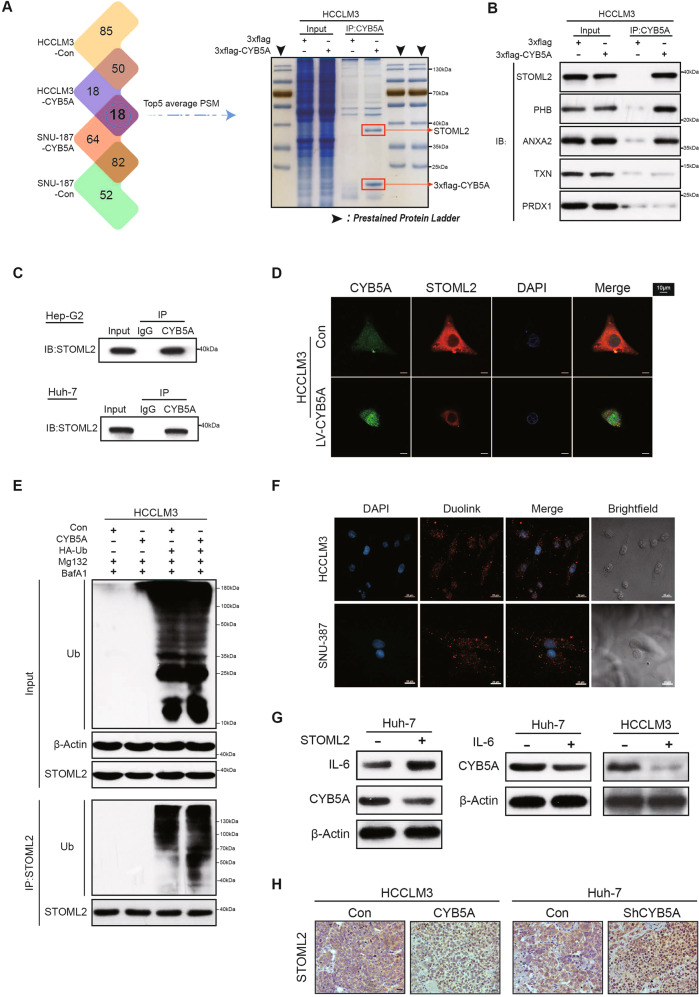
CYB5A inhibits the STAT3 signaling pathway by inhibiting STOML2. **A** The whole-cell lysates obtained from HCC cell lines were co-immunoprecipitated by Flag antibody and analyzed by liquid chromatography-tandem mass spectrometry (LC-MS). And the Coomassie-stained SDS–PAGE analysis of washed beads. **B** The results were obtained and the top five proteins in PSM were selected for WB verification. **C** The whole-cell lysates obtained from untreated Huh-7 cell lines and Hep-G2 cell lines were co-immunoprecipitated by CYB5A antibody. **D** IF demonstrating the colocalization of CYB5A and STOML2 HCC cells, also shown in Supplementary fig. 5–1A. **E** HCCLM3-Con and HCCLM3-CYB5A cell lines transfected with HA-Ub were treated with Baf A1 and MG-132. The cell extracts were analyzed by co-IP and western blot. **F** Duolink PLA demonstrating the close proximity of CYB5A and STOML2 in HCC cells. **G** The expression changes of IL-6 and CYB5A after STOML2 overexpression were demonstrated by western blot assay (left panel). And The change of CYB5A expression in Huh-7 and HCCLM3 cells treated with IL-6 (20 ng/mL, 24 h) was demonstrated by WB assay (right panel). **H** STOML2 levels in lung xenograft tumors are shown by IHC. In our rescue test (in vivo*,* in vitro and at protein expression levels), we used lentivirus to regulate STOML2 expression to confirm that CYB5A regulates STOML2 (Supplementary figs. 5–1B, 5–2, 5–3, 5–4).

To further study the possible mechanisms underlying the superior tumor-suppressor effect of CYB5A, we utilized co-immunoprecipitation (co-IP) and mass spectrometry analysis (Supplementary data 5). The top 5 peptide spectrum matches (PSM) were confirmed by western blotting analysis, and the result demonstrated a co-precipitation of the exogenous (3xflag-CYB5A) with Stomatin Like 2 (STOML2) (Fig. 5A, B). Endogenous co-IP in Huh-7 and Hep-G2 cell lines ruled out changes in the spatial structure of exogenous proteins (Fig. 5C). IF demonstrates that the cytoplasmic colocalization of CYB5A and STOML2 in cells and CYB5A and STOML2 are negatively correlated with relative content (Fig. 5D and Supplementary fig. 5–1A).

In our rescue test (in vivo*,* in vitro and at protein expression levels), we used lentivirus to regulate STOML2 expression to confirm that CYB5A regulates STOML2 (Supplementary figs. 5–1B, 5–2, 5–3, 5–4). In order to explain the changes in STOML2 density, we performed qRT-PCR, but no significant change was found in mRNA relative contents of STOML2 and STAT3, accompanied by a significant trend in the downstream genes of STAT3 (Supplementary fig. 5–1C). TCGA database indicated that the relative RNA content of CYB5A was not significantly linked to STOML2 and STAT3, but negatively correlated with the downstream gene of STAT3 (Supplementary fig. 5–1D). We further found that CYB5A enhanced STOML2 ubiquitination, indicating that CYB5A promotes STOML2 degradation (Fig. 5E). Duolink® in situ proximity ligation assay (PLA) analysis showed a strong PLA signal between CYB5A and STOML2 in the cytoplasm (Fig. 5F).

STOML2 promotes tumor progression by upregulating IL-6 to promote STAT3 pathway [32]. IL-6 is a highly expressed cytokine in the microenvironment of HCC patients [33]. The western blotting analysis also proved that STOML2 also promoted IL-6 expression in HCC cell lines; meanwhile, we unexpectedly found that Huh-7 cells exposed to IL-6 (20 ng/mL, 24 h) inhibited the protein level of CYB5A (Fig. 5G). As a result, CYB5A and STOML2 form a loop through IL-6. IHC of lung xenograft tumors shown a CYB5A is negatively correlated with STOML2 in vivo (Fig. 5H).

### Low CYB5A and high STOML2 suggest a poor prognosis

**Fig. 6:**
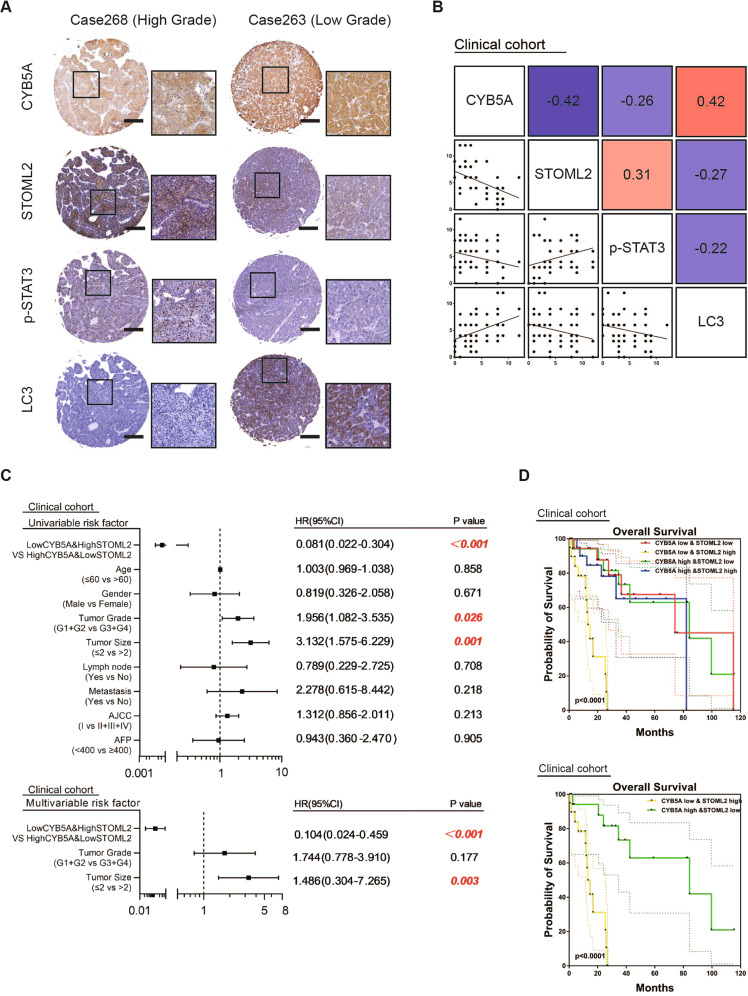
Low CYB5A and high STOML2 suggest a poor prognosis. **A** Representative images of immunohistochemical staining of CYB5A, STOML2, p-STAT3, and LC-3B in human HCC tumor sections (*n* = 80). **B** Correlation between immunohistochemistry staining intensity for CYB5A, STOML2, p-STAT3, and LC-3B in human HCC tumor sections (*n* = 80). **C** Univariate and Multivariate analyses were performed in the clinical cohort. The bar corresponds to 95% confidence intervals. **D** OS in the clinical cohort.

Similarly, IHC was performed on HCC samples from 80 clinical patients, resulting in a malignant degree of hepatocellular carcinoma, lower CYB5A and LC3 expression levels, and higher STOML2 and p-STAT3 expression levels (Fig. 6A, B).

Moreover, Cox regression analysis indicated that combining low CYB5A expression and high STOML2 expression was an independent risk factor for OS of HCC patients, and the combination indicated a worse overall survival (OS) (Fig. 6C, D, Supplementary fig. 6–1A, B). In summary, we stated that CYB5A regulates the STAT3 pathway by inhibiting the protein expression level and not the RNA level of STOML2.

### CYB5A and Ruxolitinib synergically inhibit HCC metastasis

**Fig. 7:**
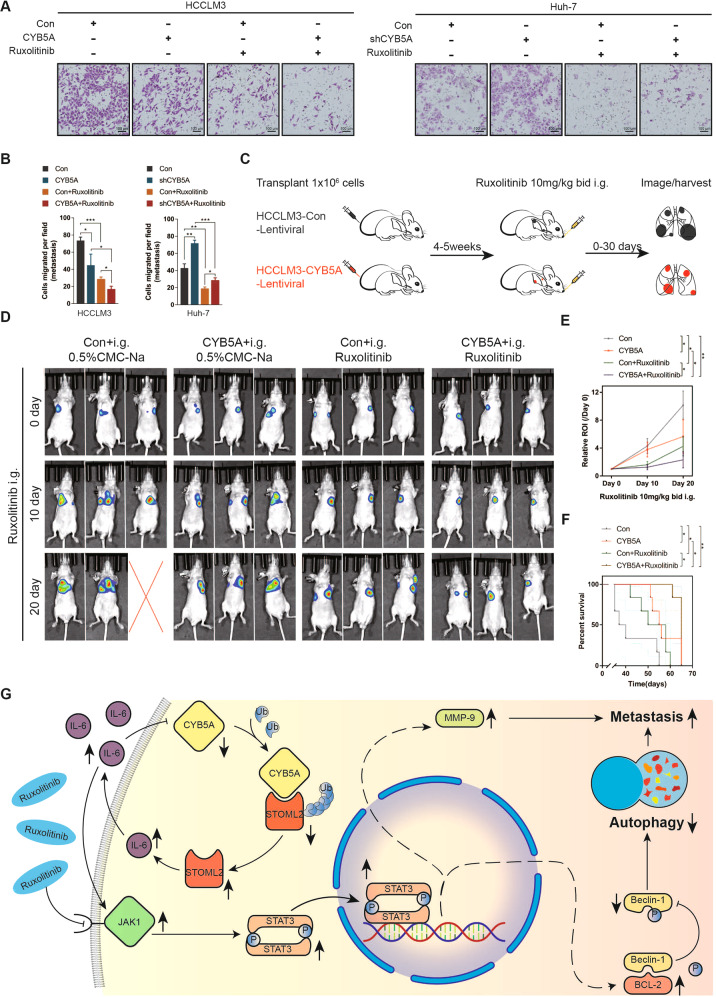
Ruxolitinib was more effective in inhibiting HCC metastasis at high CYB5A levels. **A**, **B** Tranwell assay and their statistical analysis of Ruxolitinib (50 μM, 24 h)-treated HCCLM3 and Huh-7 cells. **C** Schema for assessing the influence of Ruxolitinib in established tumors. Nude mice were given Ruxolitinib (10 mg/kg, bid, i.g.) after tumors were established in the lung xenograft HCC model. BLI was performed on 0, 10, and 20 days of administration, and ROI was measured. Bid: twice a day; i.g.: gastric injection (*n* = 6). **D** Typical image of BLI. **E**, **F** The ROI growth rate and OS of mouse treated with Ruxolitinib. ROI = region of interest. **G** Schematic diagram of the relationship among CYB5A, JAK1/STAT3 pathway, autophagy, and HCC metastasis.

To extensively investigate CYB5A clinical significance, a clinically available oral JAK1 inhibitor [30] inhibits tumor cell survival by activating autophagy [34, 35], and Ruxolitinib was applied to our experiment. Ruxolitinib can inhibit HCC cell lines’ proliferation, but JAK1 inhibitor has not been used clinically to treat HCC patients. As far as we know, our experiment is the first to explore Ruxolitinib’s impact on metastasis of HCC cells. Ruxolitinib could significantly inhibit the number of HCCLM3 cells and Huh-7 cells passing through transwell chamber, and in the same cell line treated with the same drug, the more CYB5A expression, the more obvious the inhibitory effect (Fig. 7A, B). Then, we established a Ruxolitinib treatment model of lung metastatic xenografts using HCCLM3 cell line (*n* = 6) (Fig. 7C). The region of interest (ROI) growth rate of BLI was utilized to evaluate the change in metastatic tumor tissue volume, and OS was deployed to assess mice’s prognosis. The results showed that Ruxolitinib could well inhibit the HCCLM3 metastases’ progression, and the therapeutic effect was a little more obvious when CYB5A was highly expressed (Fig. 7D–F). This suggests that, possibly, metastatic tumors in patients with high CYB5A expression exhibit more sensitivity to JAK1 inhibitors.

## Discussion

HCC is a common malignancy with an annual incidence and mortality, resulting in more than 800,000 yearly deaths. Even in the early stage of HCC, the 5-year recurrence rate is still as high as 70% [36–38]. The onset of HCC is incident, without obvious abnormal symptoms in the early stage. Most patients have developed to the late stage when HCC is diagnosed, without proper surgical treatment, leading to higher recurrence and mortality rates. Therefore, exhaustive and thorough HCC research is highly demanding.

Based on the above problems and research status, we screened three differential genes using the existing network data through rigorous bioinformatics analysis. Due to the limited length, we choose one of them, CYB5A, as this article’s topic. We found that CYB5A is closely related to tumor development as it is significantly downregulated in prostate cancer, adrenocortical cancer, breast cancer, and HCC [4, 6, 39, 40] and can inhibit the proliferation and metastasis of pancreatic cancer cells [8]. However, no research has yet been conducted to explain the mechanism, although as presented in various network databases and our validation cohort, CYB5A is characterized by low expression in HCC. To investigate the impact of CYB5A on the malignant phenotype of HCC cells, we performed several experiments in vitro. We found that although the enforced expression of CYB5A did not inhibit HCC cells’ proliferation, it significantly inhibited their metastatic ability.

Metastatic ability is a typical characteristic of HCC malignancy. Different Xenograft models were employed to simulate the top three common organ-specific metastases of HCC (intrahepatic, pulmonary, and skeletal metastases). Moreover, CYB5A was found to be strongly related to autophagy through gene sequencing. Much evidence shows that autophagy can inhibit tumor metastasis. Based on previous literature reports and experimental phenomena, we conclude a close relationship between CYB5A promoting autophagy and CYB5A inhibiting metastasis. We induced autophagy induction of HCC cells with Rapamycin and successfully inhibited metastasis. Various experiments confirmed that CYB5A stimulated autophagy of HCC cells.

As a cytochrome, CYB5A is a protein regulating cell REDOX. In this paper, the expression difference of CYB5A was found to verify its inhibition of metastasis, and there was no indication that the difference was caused by the gene mutation of CYB5A. To clarify the role of CYB5A in HCC, we looked for other possible causes. Similarly, autophagy is a key way for cells to overcome oxidative stress. Therefore, we paid special attention to the relationship between CYB5A expression and autophagy.

Iron metabolism is also a function of CYB5A. The REDOX of iron plays a key role in ferroptosis which downstream is the autophagy pathway. Ferroptosis is also a signaling pathway that shows differences after we regulate the expression of CYB5A. This is a very interesting phenomenon, which may indicate a certain relationship between CYB5A and ferroptosis. However, due to space limitation, this paper only discusses the influence of CYB5A on its downstream autophagy, and the relationship between CYB5A and ferroptosis will be further studied by our research group.

Since autophagy-related signaling pathways are complex, further studies are required to clarify the regulatory mechanisms of autophagy. We screened out the differential protein p-STAT3 by sequencing results combined with the phosphoric acid antibody chip, and detected the changes of p-Beclin-1 found in almost all the expression differences of autophagy-related proteins. Since p-STAT3 changes but total STAT3 does not, we believe that CYB5A regulates the phosphorylation level of STAT3. Fortunately, we found a change in the protein level of the upstream gene JAK1 of STAT3, highlighting that CYB5A affects the STAT3 pathway through JAK1.

Besides, STAT3/Bcl-2/Beclin-1 signaling is associated with the induction of autophagy inhibition. Previous reports have shown that Beclin-1, as an important autophagy-related protein, can bind to and be inhibited by Bcl-2 protein, thus preventing autophagy [41–43]. Besides, STAT3 can transcriptionally activate the apoptotic inhibitor protein Bcl-2, which also inhibits autophagy induction by disassociating the Bcl-2/Beclin-1 complex. Once activated, STAT3 upregulates Bcl-2 expression, leading to autophagy inhibition. In our study, after regulating STAT3 expression, the phosphorylation level of Beclin-1 was reversed, and the autophagy phenotype and metastatic phenotype were changed. These results indicated that STAT3 inhibited the induction of HCC autophagy. We hypothesized that CYB5A-mediated autophagy might occur by targeting STAT3, thereby weakening the metastatic ability of HCC cells. In fact, we found that CYB5A indeed promoted CYB5A cell autophagy and inhibited HCC metastasis in vivo and in vitro by promoting the disintegration of Bcl-2/Beclin-1 complex. To explain why CYB5A could inhibit JAK1 activity we found that CYB5A was closely linked to the oncogenic gene STOML2 using experiments on protein interactions. STOML2 is considered a star oncopromoter gene [44–47] and can activate the JAK/STAT3 pathway by stimulating IL-6 expression [32]. Our experiments showed that STOML2 could be inhibited by CYB5A. CYB5A, STOML2, and IL-6 seem to form a feedback loop.

In addition to its biological importance, our work may be relevant to clinical management of HCC patients. As CYB5A and STOML2 expression is associated with HCC recurrence risk, measuring CYB5A and STOML2 levels post-surgery may be an effective approach to predict patient outcomes. Furthermore, our data raise an important clinical question: are JAK1 inhibitors, including Ruxolitinib, suitable for HCC patients with a high CYB5A expression? Our animal experiments suggest that metastatic HCC patients with a high CYB5A expression may be treated with Ruxolitinib. Thus, detecting CYB5A and its associated pathway and differentially managing patients with different CYB5A levels is crucial.

## Supplementary information

Supplemental Figures

Supplemental Figure legends

Supplemental Materials and Methods

Supplementary data 1

Supplementary data 2

Supplementary data 3

Supplementary data 4

Supplementary data 5

Supplementary data 6

Supplementary data 7

Original Data File

aj-checklist

Co-author information changes statement

## Data Availability

All data generated and analyzed during this study are included in this published article and its additional file.
