# Supplementary material for: Cytochrome B5 type A alleviates HCC metastasis via regulating STOML2 related autophagy and promoting sensitivity to ruxolitinib
Source: Cell Death Dis. 2022 Jul 18;13(7):623. doi: 10.1038/s41419-022-05053-8 (PMC9293983; doi:10.1038/s41419-022-05053-8)

S Figure 1-1

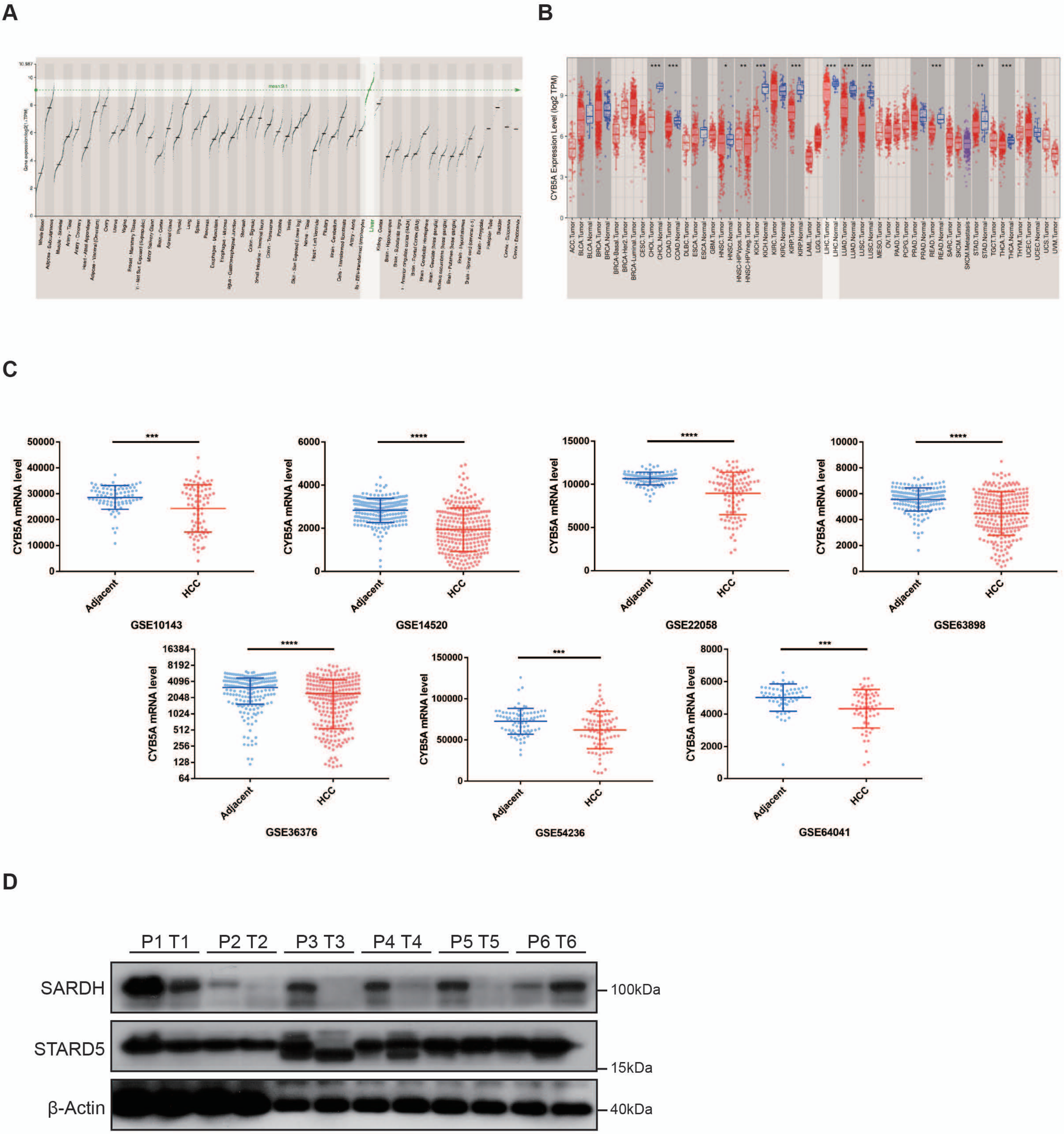
A
B
CYB5A Expression Level (log2 TPM)
C
CYB5A mRNA level
Adjacent
HCC
GSE10143
GSE14520
GSE22058
GSE63898
GSE36376
GSE54236
GSE64041
D
P1 T1
P2 T2
P3 T3
P4 T4
P5 T5
P6 T6
SARDH
STARD5
β-Actin
100kDa
15kDa
40kDa

S Figure 1-2

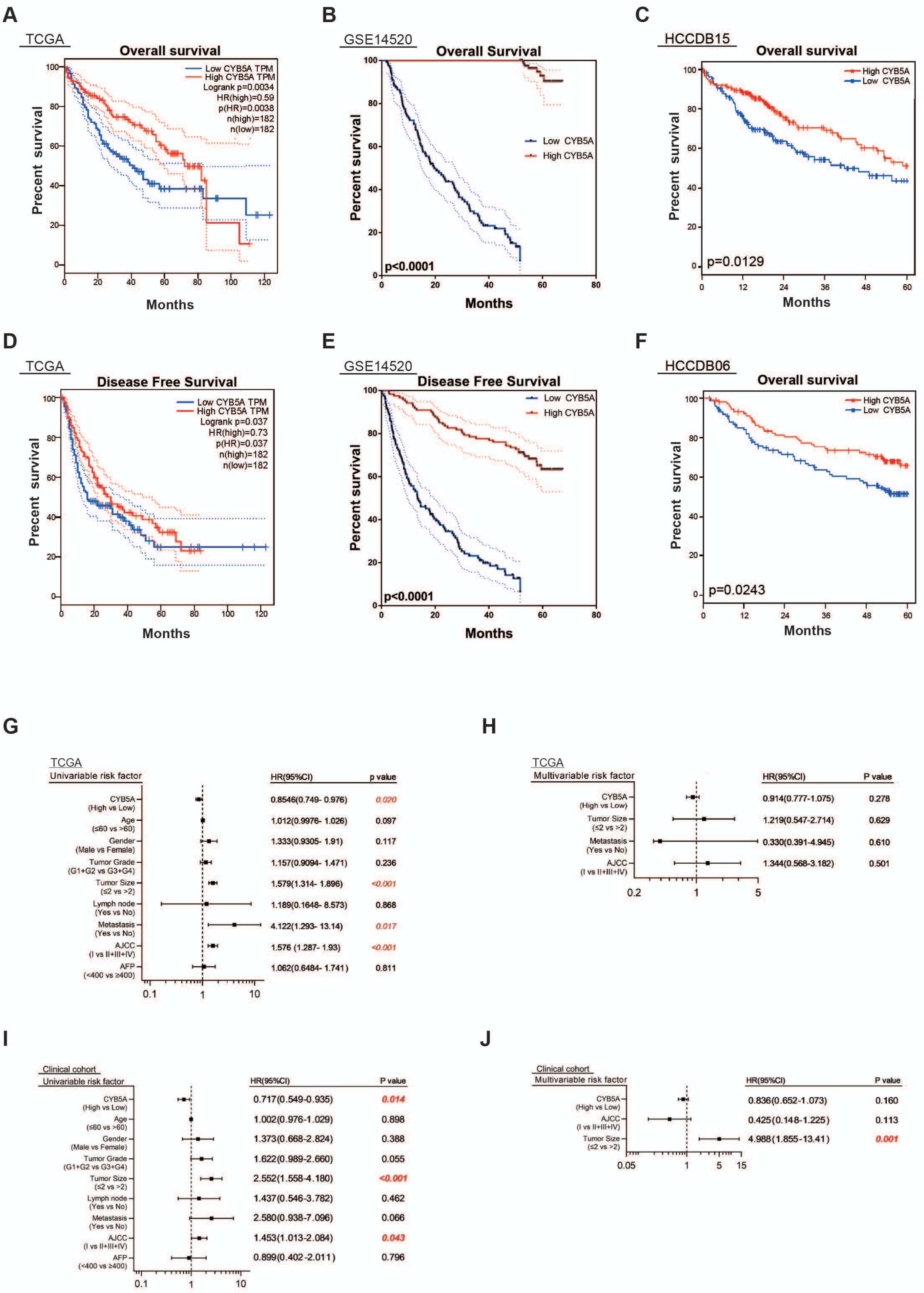
A
TCGA
Overall survival
Low CYB5A TPM
High CYB5A TPM
Logrank p=0.0034
HR(high)=0.59
p(HR)=0.0038
n(high)=182
n(low)=182
Precent survival
Months
B
GSE14520
Overall Survival
Low CYB5A
High CYB5A
Percent survival
p<0.0001
Months
C
HCCDB15
Overall survival
High CYB5A
Low CYB5A
Precent survival
p=0.0129
Months
D
TCGA
Disease Free Survival
Low CYB5A TPM
High CYB5A TPM
Logrank p=0.037
HR(high)=0.73
p(HR)=0.037
n(high)=182
n(low)=182
Precent survival
Months
E
GSE14520
Disease Free Survival
Low CYB5A
High CYB5A
Percent survival
p<0.0001
Months
F
HCCDB06
Overall survival
High CYB5A
Low CYB5A
Precent survival
p=0.0243
Months
G
TCGA
Univariable risk factor
HR(95%CI)
p value
CYB5A (High vs Low) 0.8546(0.749- 0.976) 0.020
Age (≤60 vs >60) 1.012(0.9976- 1.026) 0.097
Gender (Male vs Female) 1.333(0.9305- 1.91) 0.117
Tumor Grade (G1+G2 vs G3+G4) 1.157(0.9094- 1.471) 0.236
Tumor Size (≤2 vs >2) 1.579(1.314- 1.896) <0.001
Lymph node (Yes vs No) 1.189(0.1648- 8.573) 0.868
Metastasis (Yes vs No) 4.122(1.293- 13.14) 0.017
AJCC (I vs II+III+IV) 1.576 (1.287- 1.93) <0.001
AFP (<400 vs ≥400) 1.062(0.6484- 1.741) 0.811
H
TCGA
Multivariable risk factor
HR(95%CI)
P value
CYB5A (High vs Low) 0.914(0.777-1.075) 0.278
Tumor Size (≤2 vs >2) 1.219(0.547-2.714) 0.629
Metastasis (Yes vs No) 0.330(0.391-4.945) 0.610
AJCC (I vs II+III+IV) 1.344(0.568-3.182) 0.501
I
Clinical cohort
Univariable risk factor
HR(95%CI)
P value
CYB5A (High vs Low) 0.717(0.549-0.935) 0.014
Age (≤60 vs >60) 1.002(0.976-1.029) 0.898
Gender (Male vs Female) 1.373(0.668-2.824) 0.388
Tumor Grade (G1+G2 vs G3+G4) 1.622(0.989-2.660) 0.055
Tumor Size (≤2 vs >2) 2.552(1.558-4.180) <0.001
Lymph node (Yes vs No) 1.437(0.546-3.782) 0.462
Metastasis (Yes vs No) 2.580(0.938-7.096) 0.066
AJCC (I vs II+III+IV) 1.453(1.013-2.084) 0.043
AFP (<400 vs ≥400) 0.899(0.402-2.011) 0.796
J
Clinical cohort
Multivariable risk factor
HR(95%CI)
P value
CYB5A (High vs Low) 0.836(0.652-1.073) 0.160
AJCC (I vs II+III+IV) 0.425(0.148-1.225) 0.113
Tumor Size (≤2 vs >2) 4.988(1.855-13.41) 0.001

S Figure 2-1

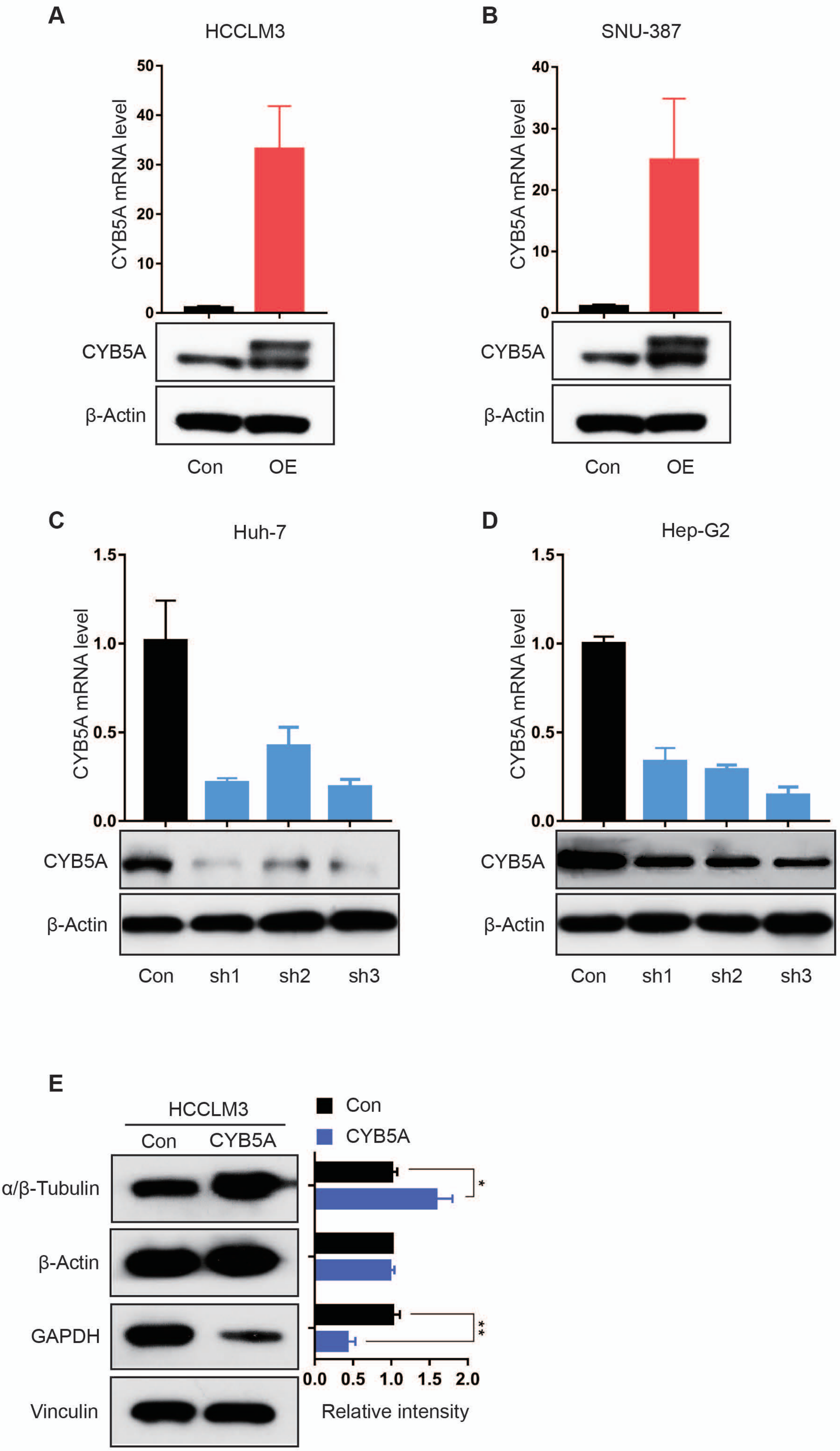

A
HCCLM3
CYB5A mRNA level
50
40
30
20
10
0
CYB5A
β-Actin
Con
OE
B
SNU-387
CYB5A mRNA level
40
30
20
10
0
CYB5A
β-Actin
Con
OE
C
Huh-7
CYB5A mRNA level
1.5
1.0
0.5
0.0
CYB5A
β-Actin
Con
sh1
sh2
sh3
D
Hep-G2
CYB5A mRNA level
1.5
1.0
0.5
0.0
CYB5A
β-Actin
Con
sh1
sh2
sh3
E
HCCLM3
Con
CYB5A
α/β-Tubulin
β-Actin
GAPDH
Vinculin
Con
CYB5A
*
**
0.0
0.5
1.0
1.5
2.0
Relative intensity

S Figure 2-2

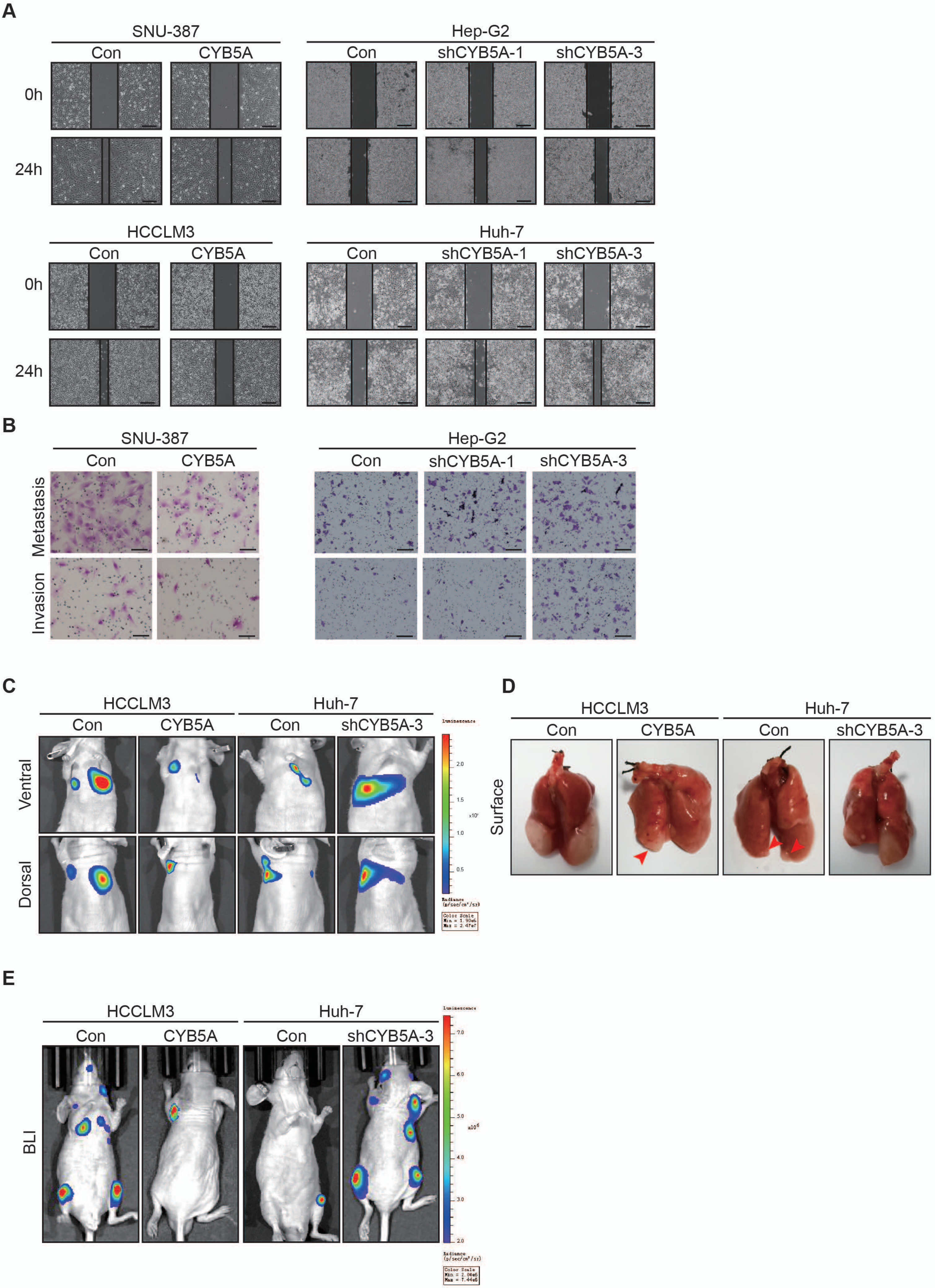

A
SNU-387
Con
CYB5A
Hep-G2
Con
shCYB5A-1
shCYB5A-3
0h
24h
HCCLM3
Con
CYB5A
Huh-7
Con
shCYB5A-1
shCYB5A-3
0h
24h
B
SNU-387
Con
CYB5A
Hep-G2
Con
shCYB5A-1
shCYB5A-3
Metastasis
Invasion
C
HCCLM3
Con
CYB5A
Huh-7
Con
shCYB5A-3
Ventral
Dorsal
D
HCCLM3
Con
CYB5A
Huh-7
Con
shCYB5A-3
Surface
E
HCCLM3
Con
CYB5A
Huh-7
Con
shCYB5A-3
BLI

S Figure 2-3

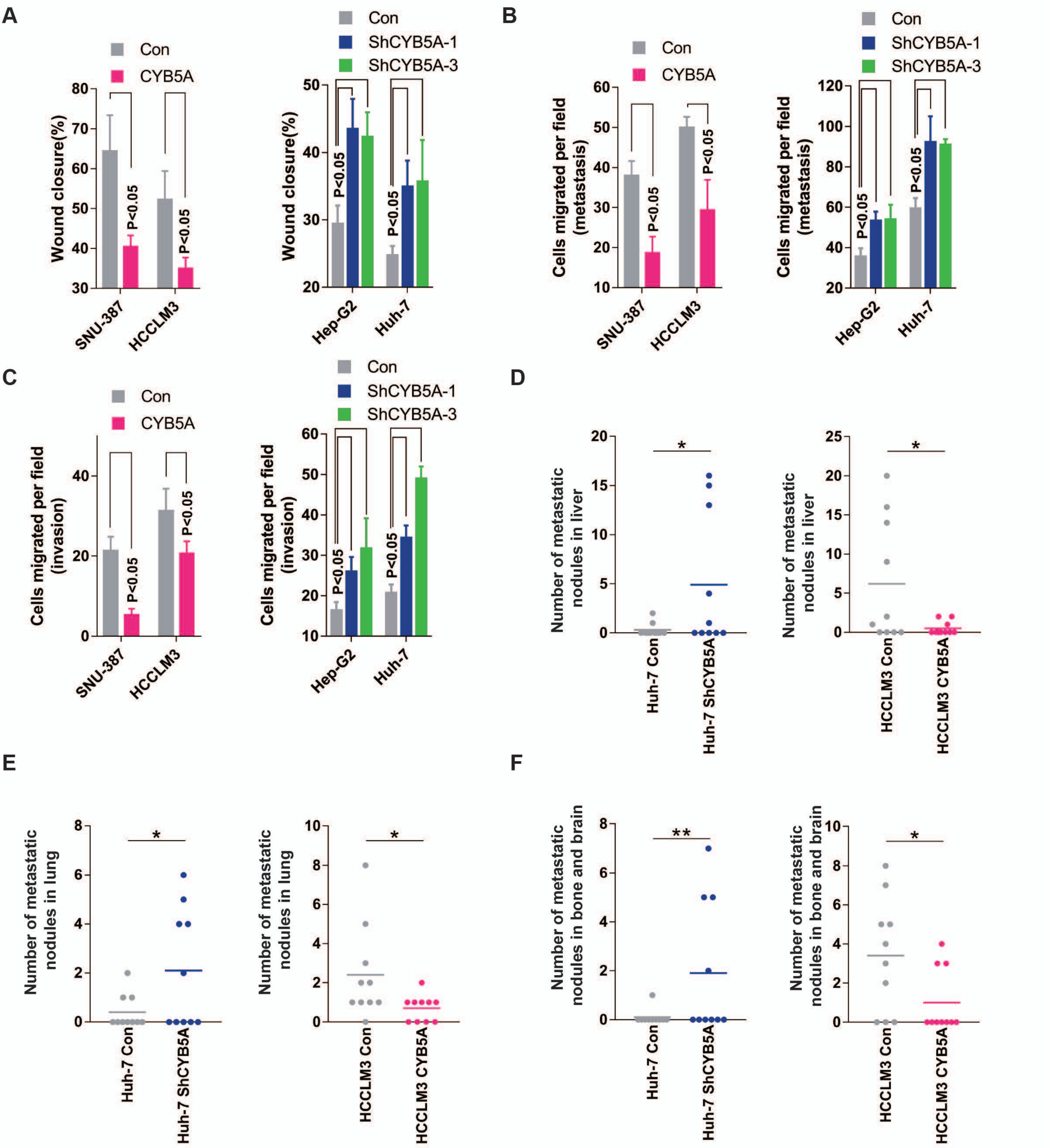

G

Incidence of liver metastasis

	Liver metastasis
Huh-7 Con	2/10
Huh-7 ShCYB5A	5/10
HCCLM3 Con	6/10
HCCLM3 CYB5A	3/10

Incidence of lung metastasis

	Lung metastasis
Huh-7 Con	3/10
Huh-7 ShCYB5A	5/10
HCCLM3 Con	9/10
HCCLM3 CYB5A	6/10

Incidence of bone&brain metastasis

	Bone&brain metastasis
Huh-7 Con	1/10
Huh-7 ShCYB5A	4/10
HCCLM3 Con	7/10
HCCLM3 CYB5A	3/10

S Figure 2-4

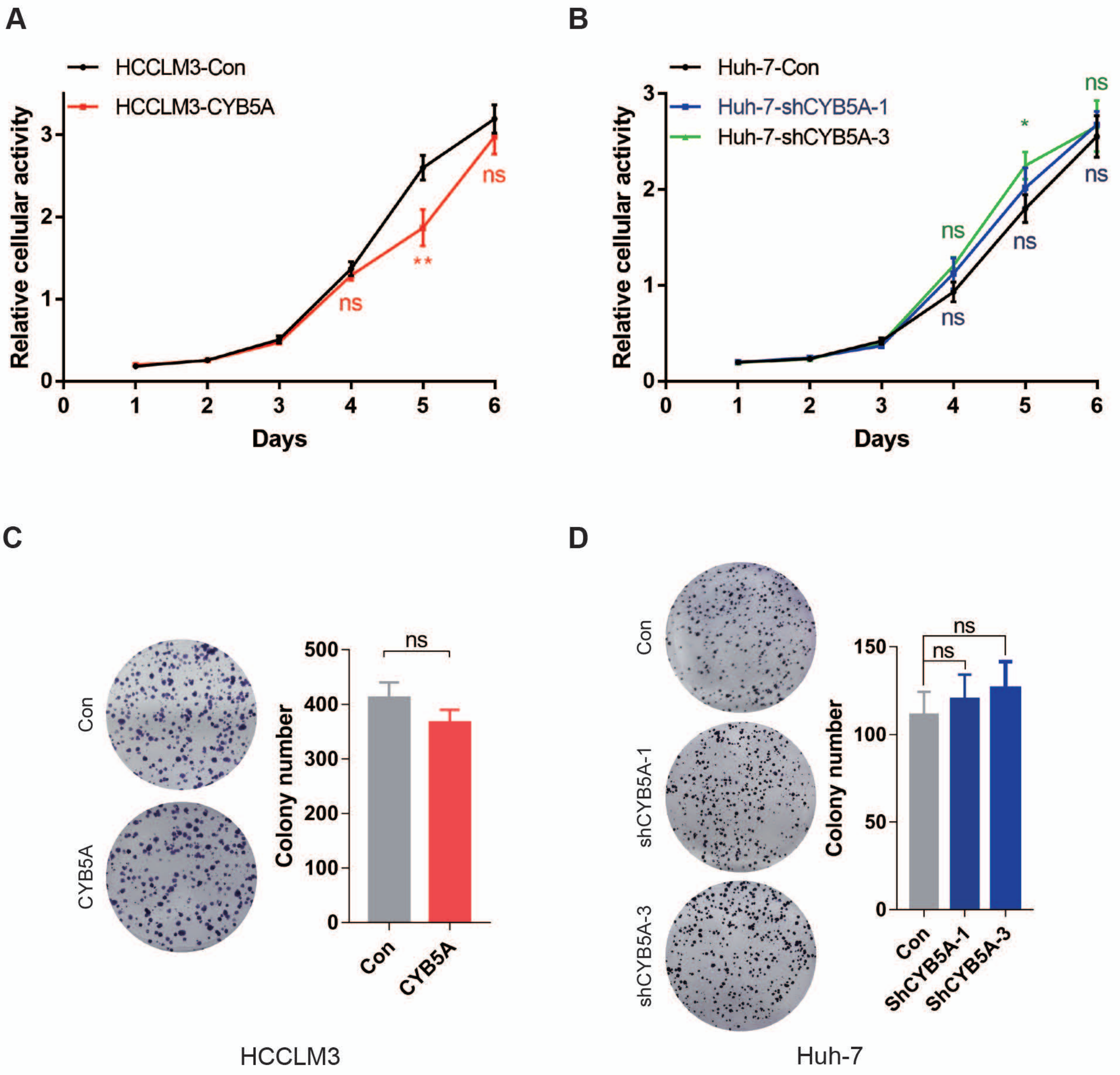
A
HCCLM3-Con
HCCLM3-CYB5A
Relative cellular activity
Days
ns
**
ns
B
Huh-7-Con
Huh-7-shCYB5A-1
Huh-7-shCYB5A-3
Relative cellular activity
Days
ns
*
ns
ns
ns
ns
C
Con
CYB5A
Colony number
ns
HCCLM3
D
Con
shCYB5A-1
shCYB5A-3
Colony number
ns
ns
ShCYB5A-1
ShCYB5A-3
Huh-7

S Figure 3-1

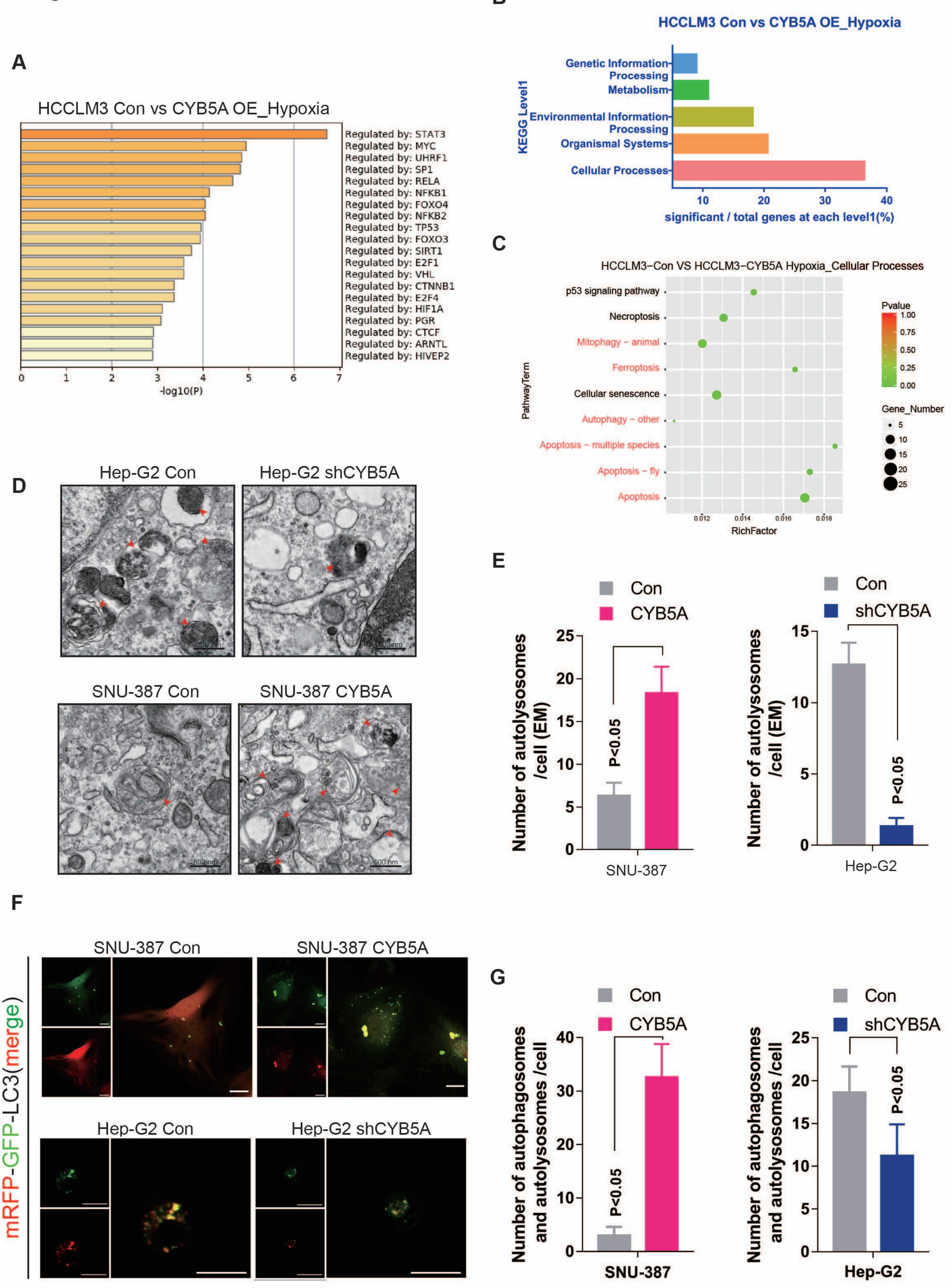
A
HCCLM3 Con vs CYB5A OE_Hypoxia
Regulated by: STAT3
Regulated by: MYC
Regulated by: UHRF1
Regulated by: SP1
Regulated by: RELA
Regulated by: NFKB1
Regulated by: FOXO4
Regulated by: NFKB2
Regulated by: TP53
Regulated by: FOXO3
Regulated by: SIRT1
Regulated by: E2F1
Regulated by: VHL
Regulated by: CTNNB1
Regulated by: E2F4
Regulated by: HIF1A
Regulated by: PGR
Regulated by: CTCF
Regulated by: ARNTL
Regulated by: HIVEP2
-log10(P)
B
HCCLM3 Con vs CYB5A OE_Hypoxia
KEGG Level1
Genetic Information Processing
Metabolism
Environmental Information Processing
Organismal Systems
Cellular Processes
significant / total genes at each level1(%)
C
HCCLM3-Con VS HCCLM3-CYB5A Hypoxia_Cellular Processes
PathwayTerm
p53 signaling pathway
Necroptosis
Mitophagy - animal
Ferroptosis
Cellular senescence
Autophagy - other
Apoptosis - multiple species
Apoptosis - fly
Apoptosis
RichFactor
Pvalue
Gene_Number
D
Hep-G2 Con
Hep-G2 shCYB5A
SNU-387 Con
SNU-387 CYB5A
500 nm
E
Con
CYB5A
shCYB5A
Number of autolysosomes /cell (EM)
P<0.05
SNU-387
Hep-G2
F
SNU-387 Con
SNU-387 CYB5A
Hep-G2 Con
Hep-G2 shCYB5A
mRFP-GFP-LC3(merge)
G
Con
CYB5A
shCYB5A
Number of autophagosomes and autolysosomes /cell
P<0.05
SNU-387
Hep-G2

S Figure 3-2

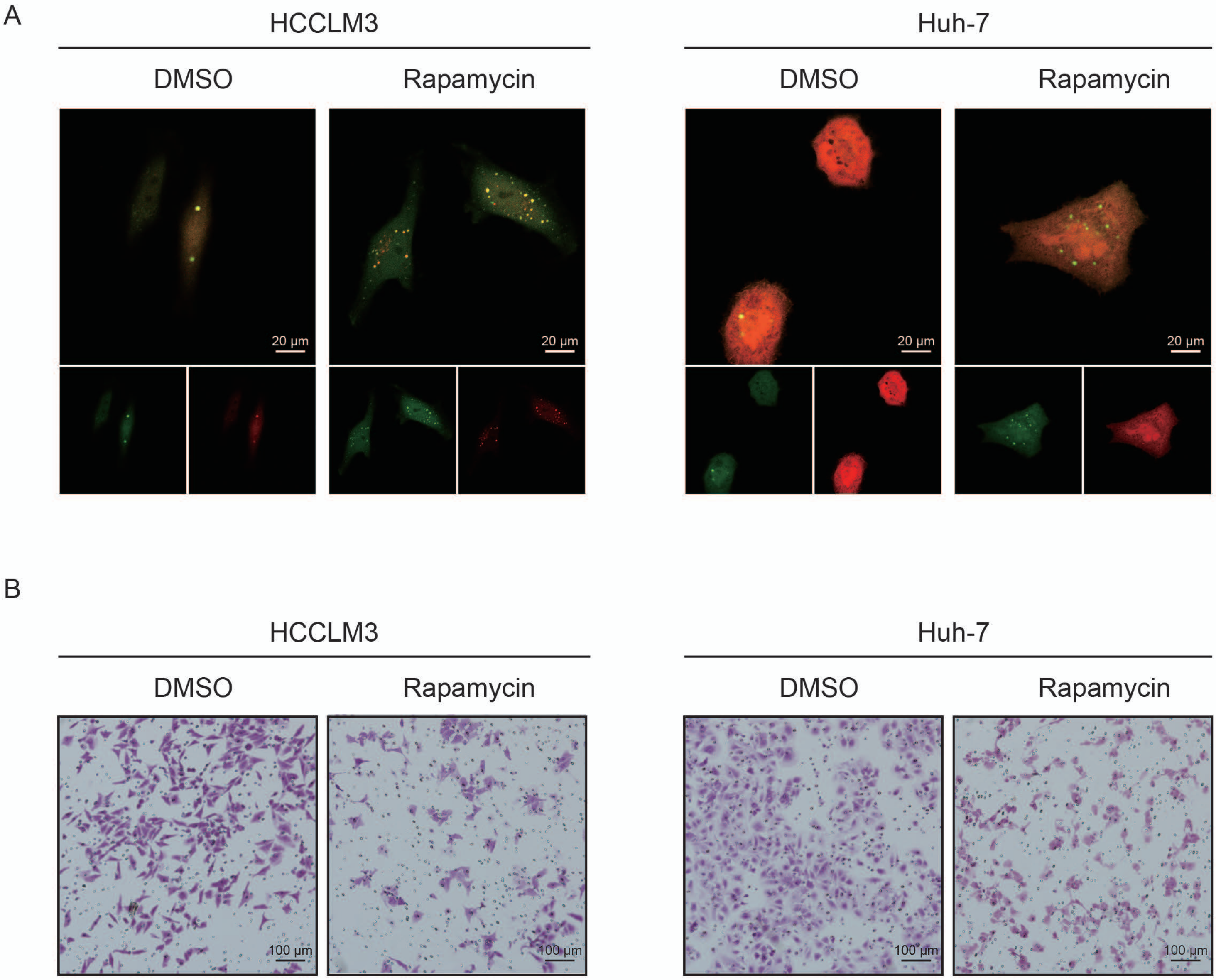
A
HCCLM3
Huh-7
DMSO
Rapamycin
DMSO
Rapamycin
20 μm
20 μm
20 μm
20 μm
B
HCCLM3
Huh-7
DMSO
Rapamycin
DMSO
Rapamycin
100 μm
100 μm
100 μm
100 μm

S Figure 3-3

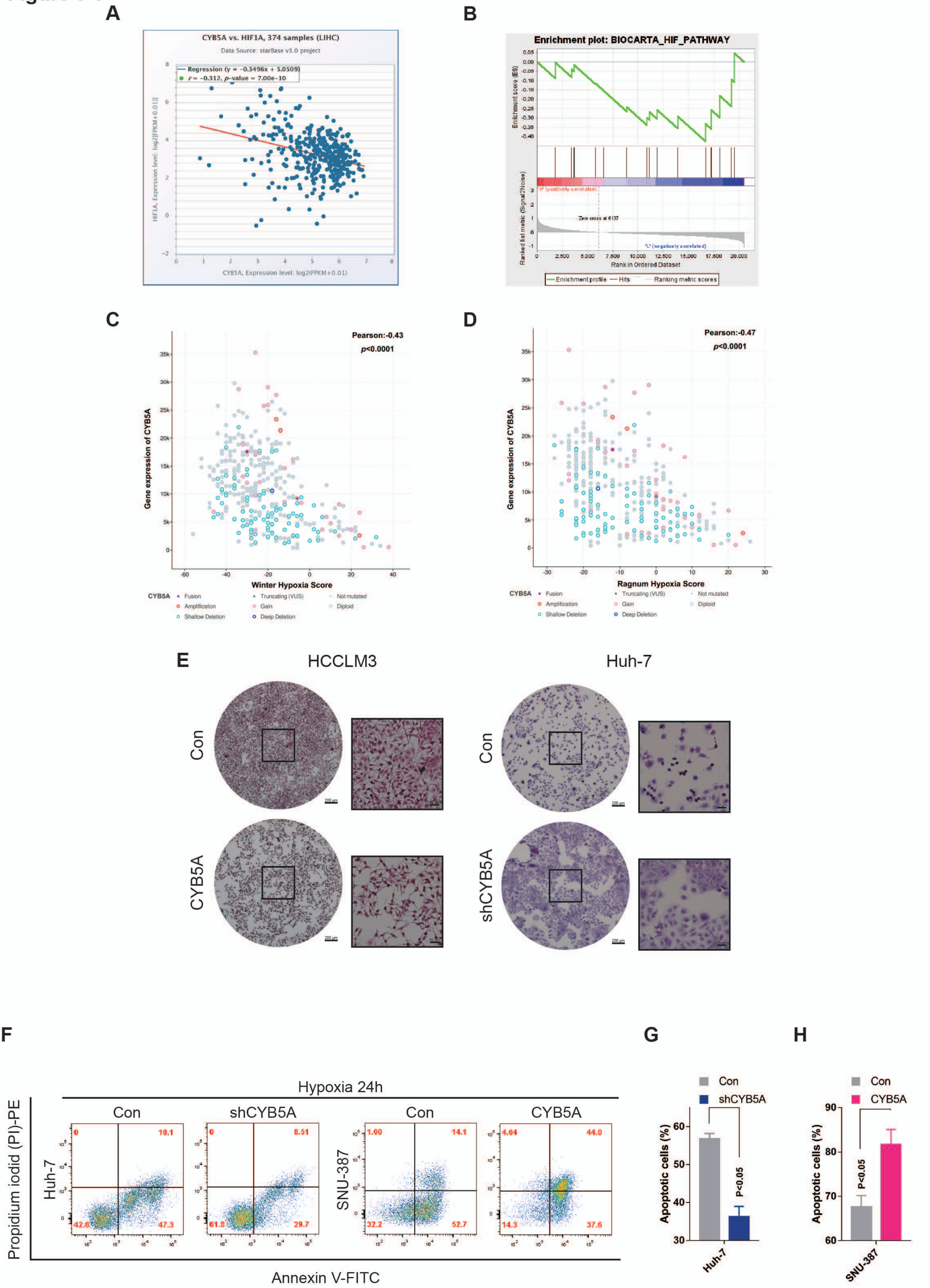
A
CYB5A vs. HIF1A, 374 samples (LIHC)
Data Source: starBase v3.0 project
Regression (y = -0.3496x + 5.0509)
r = -0.312, p-value = 7.00e-10
HIF1A, Expression level: log2(FPKM+0.01)
CYB5A, Expression level: log2(FPKM+0.01)
B
Enrichment plot: BIOCARTA_HIF_PATHWAY
Enrichment score (ES)
Ranked list metric (Signal2Noise)
Zero cross at 6137
Rank in Ordered Dataset
Enrichment profile
Hits
Ranking metric scores
C
Pearson:-0.43
p<0.0001
Gene expression of CYB5A
Winter Hypoxia Score
CYB5A
Fusion
Truncating (VUS)
Not mutated
Amplification
Gain
Diploid
Shallow Deletion
Deep Deletion
D
Pearson:-0.47
p<0.0001
Gene expression of CYB5A
Ragnum Hypoxia Score
E
HCCLM3
Huh-7
Con
CYB5A
shCYB5A
F
Hypoxia 24h
Propidium iodid (PI)-PE
Annexin V-FITC
Huh-7
SNU-387
G
Apoptotic cells (%)
P<0.05
H
80
90

S Figure 4-1

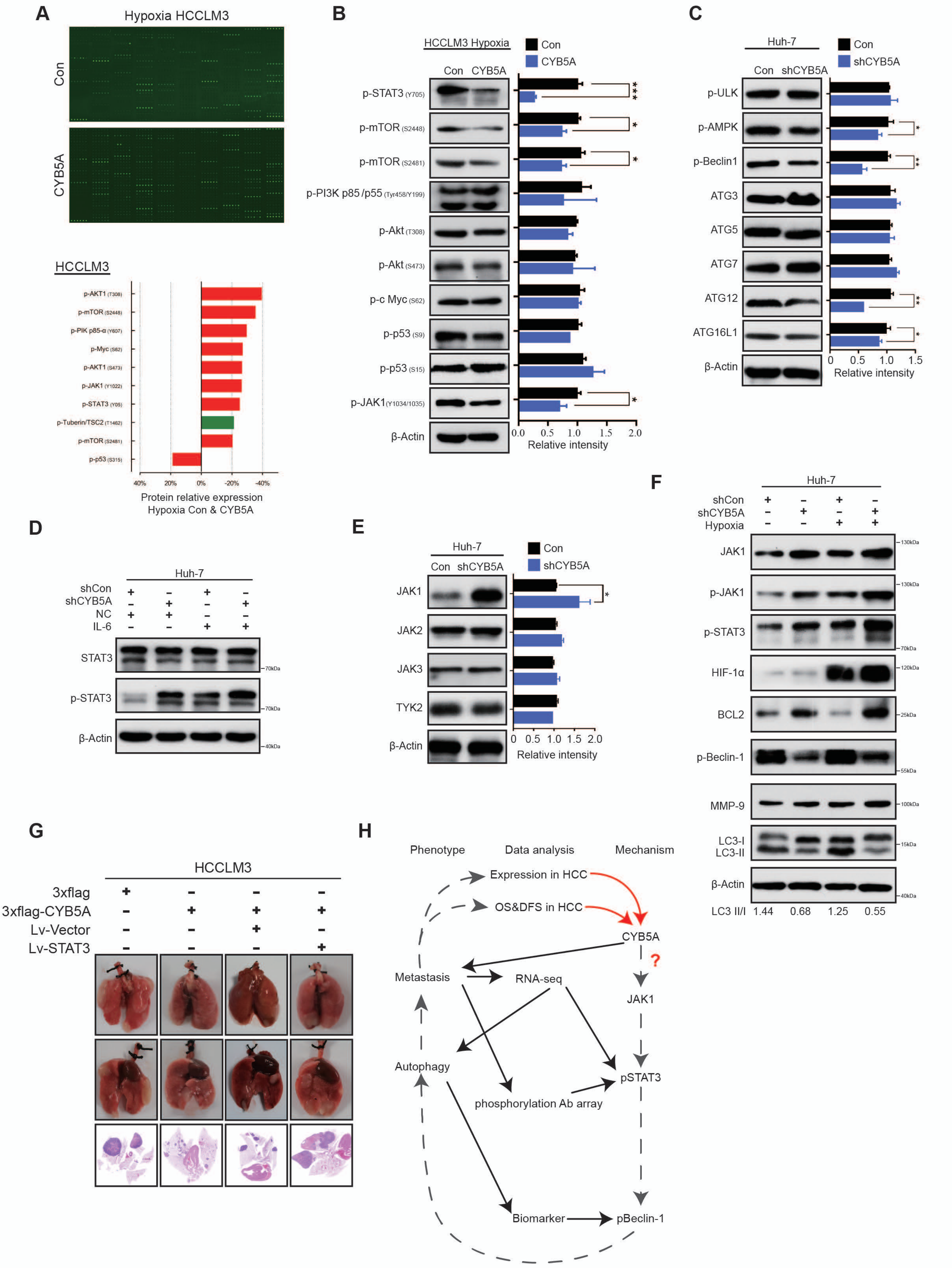

A
Hypoxia HCCLM3
Con
CYB5A
HCCLM3
p-AKT1 (T308)
p-mTOR (S2448)
p-PIK p85-α (Y607)
p-Myc (S62)
p-AKT1 (S473)
p-JAK1 (Y1022)
p-STAT3 (Y05)
p-Tuberin/TSC2 (T1462)
p-mTOR (S2481)
p-p53 (S315)
40% 20% 0% -20% -40%
Protein relative expression
Hypoxia Con & CYB5A
B
HCCLM3 Hypoxia
Con CYB5A
p-STAT3 (Y705)
p-mTOR (S2448)
p-mTOR (S2481)
p-PI3K p85/p55 (Tyr458/Y199)
p-Akt (T308)
p-Akt (S473)
p-c Myc (S62)
p-p53 (S9)
p-p53 (S15)
p-JAK1 (Y1034/1035)
β-Actin
0.0 0.5 1.0 1.5 2.0
Relative intensity
C
Huh-7
Con shCYB5A
p-ULK
p-AMPK
p-Beclin1
ATG3
ATG5
ATG7
ATG12
ATG16L1
β-Actin
0 0.5 1.0 1.5
Relative intensity
D
Huh-7
shCon
shCYB5A
NC
IL-6
STAT3
p-STAT3
β-Actin
E
Huh-7
Con shCYB5A
JAK1
JAK2
JAK3
TYK2
β-Actin
0 0.5 1.0 1.5 2.0
Relative intensity
F
Huh-7
shCon
shCYB5A
Hypoxia
JAK1
p-JAK1
p-STAT3
HIF-1α
BCL2
p-Beclin-1
MMP-9
LC3-I
LC3-II
β-Actin
LC3 II/I 1.44 0.68 1.25 0.55
G
HCCLM3
3xflag
3xflag-CYB5A
Lv-Vector
Lv-STAT3
H
Phenotype
Data analysis
Mechanism
Expression in HCC
OS&DFS in HCC
CYB5A
Metastasis
RNA-seq
JAK1
Autophagy
pSTAT3
phosphorylation Ab array
Biomarker
pBeclin-1

S Figure 4-2

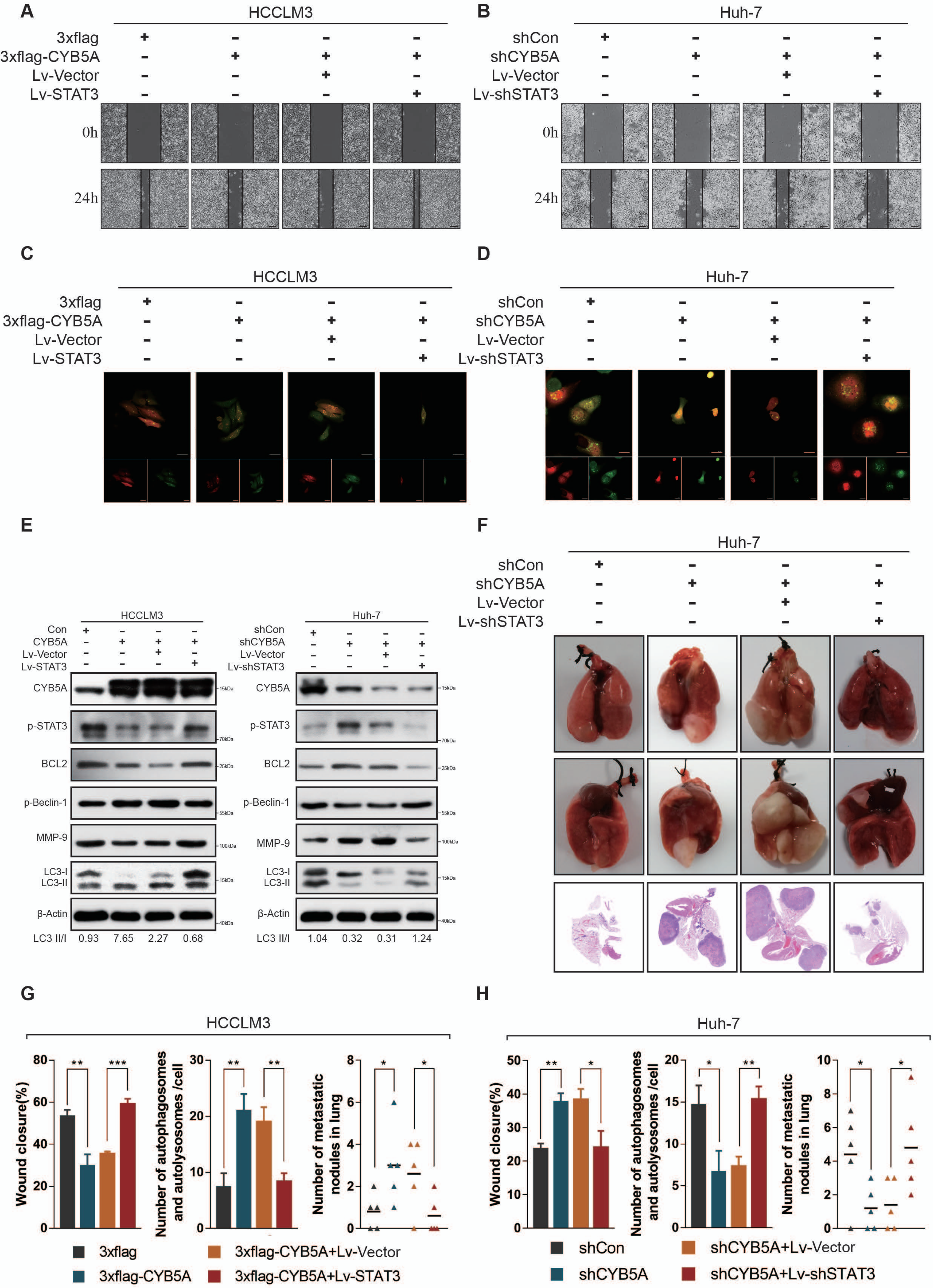

A
HCCLM3
3xflag
3xflag-CYB5A
Lv-Vector
Lv-STAT3
0h
24h
B
Huh-7
shCon
shCYB5A
Lv-Vector
Lv-shSTAT3
0h
24h
C
HCCLM3
3xflag
3xflag-CYB5A
Lv-Vector
Lv-STAT3
D
Huh-7
shCon
shCYB5A
Lv-Vector
Lv-shSTAT3
E
HCCLM3
Con
CYB5A
Lv-Vector
Lv-STAT3
CYB5A
p-STAT3
BCL2
p-Beclin-1
MMP-9
LC3-I
LC3-II
β-Actin
LC3 II/I 0.93 7.65 2.27 0.68
Huh-7
shCon
shCYB5A
Lv-Vector
Lv-shSTAT3
LC3 II/I 1.04 0.32 0.31 1.24
F
Huh-7
shCon
shCYB5A
Lv-Vector
Lv-shSTAT3
G
HCCLM3
Wound closure(%)
Number of autophagosomes and autolysosomes /cell
Number of metastatic nodules in lung
3xflag
3xflag-CYB5A
3xflag-CYB5A+Lv-Vector
3xflag-CYB5A+Lv-STAT3
H
Huh-7
Wound closure(%)
Number of autophagosomes and autolysosomes /cell
Number of metastatic nodules in lung
shCon
shCYB5A
shCYB5A+Lv-Vector
shCYB5A+Lv-shSTAT3

S Figure 5-1

A

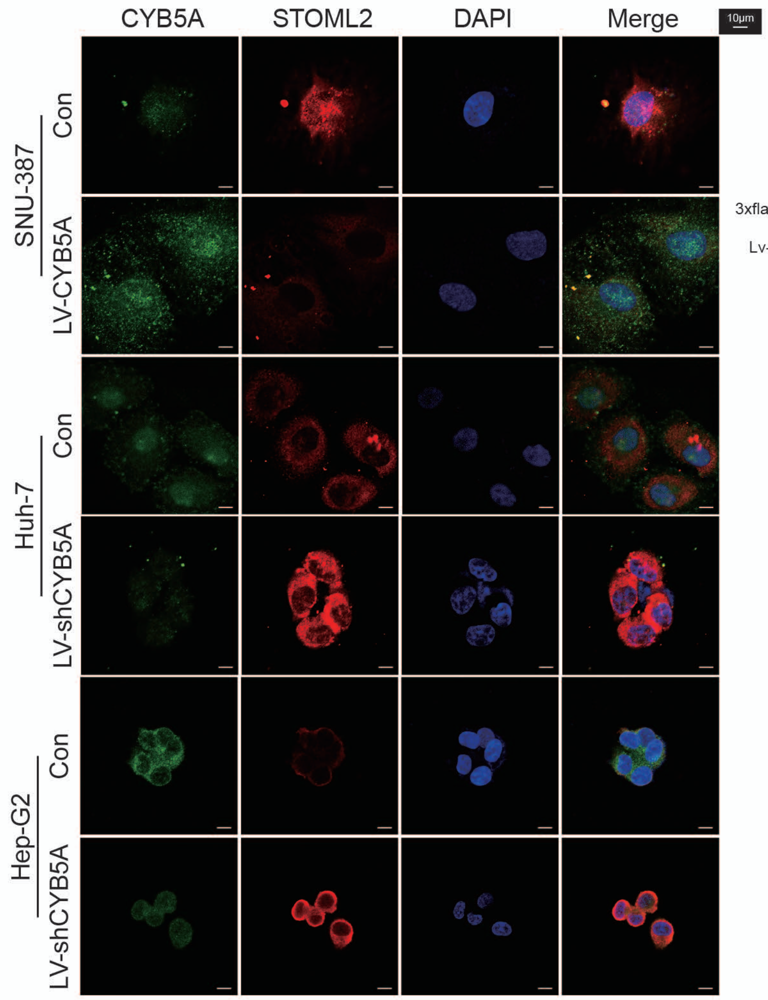

B

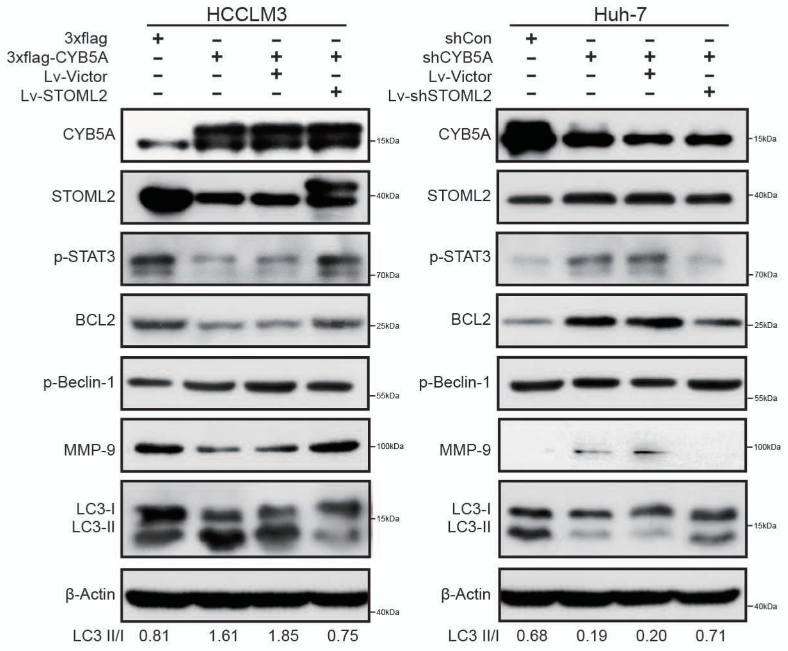

C

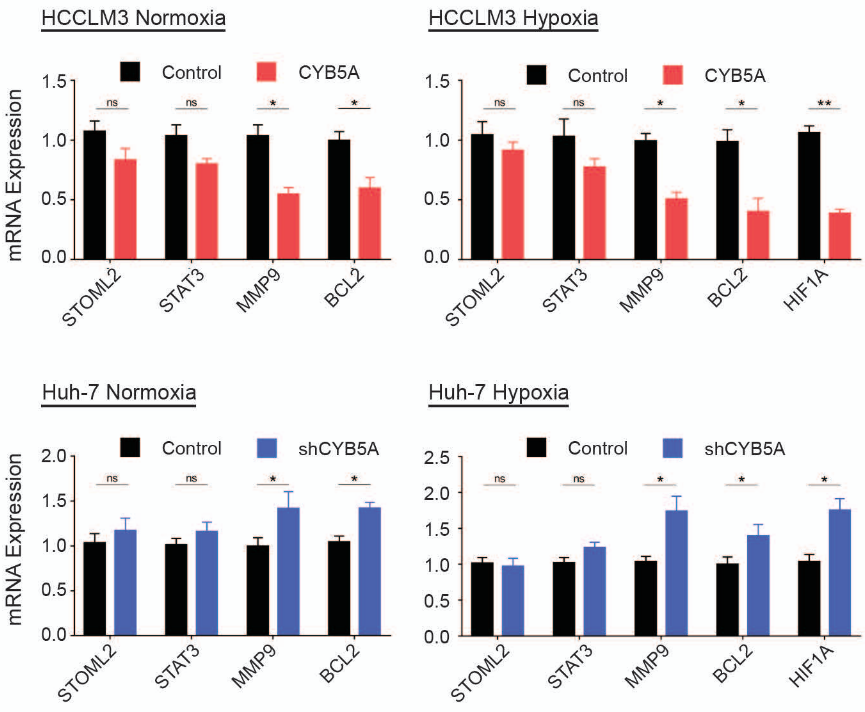

D

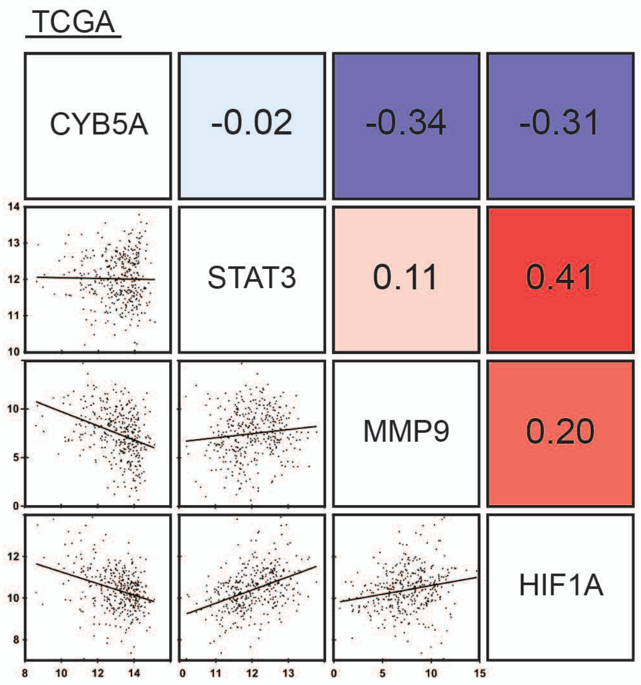

S Figure 5-2

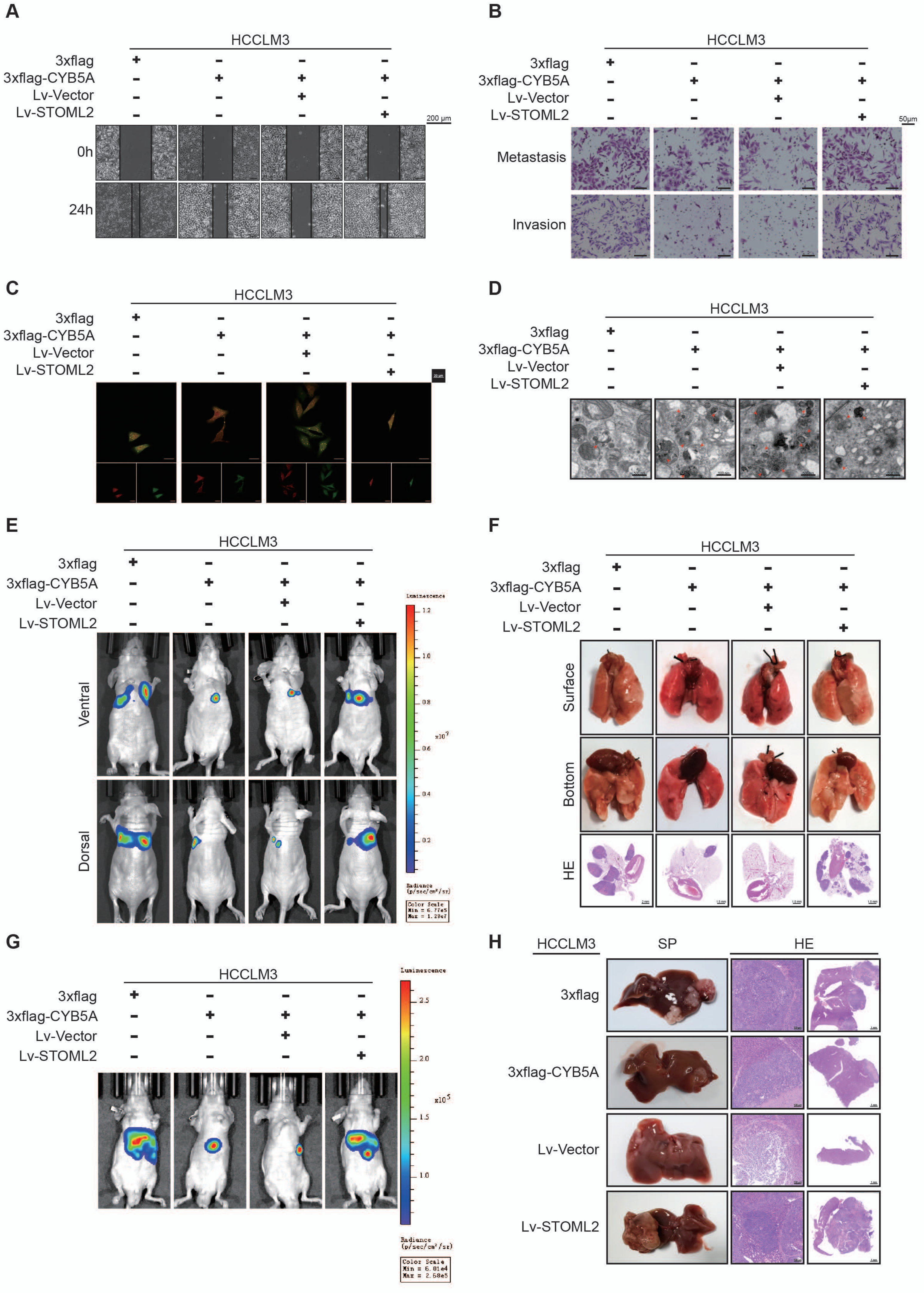
A
HCCLM3
3xflag
3xflag-CYB5A
Lv-Vector
Lv-STOML2
200 μm
0h
24h
B
HCCLM3
3xflag
3xflag-CYB5A
Lv-Vector
Lv-STOML2
50μm
Metastasis
Invasion
C
HCCLM3
3xflag
3xflag-CYB5A
Lv-Vector
Lv-STOML2
D
HCCLM3
3xflag
3xflag-CYB5A
Lv-Vector
Lv-STOML2
E
HCCLM3
3xflag
3xflag-CYB5A
Lv-Vector
Lv-STOML2
Ventral
Dorsal
Luminescence
x10^7
Radiance (p/sec/cm²/sr)
Color Scale
Min = 6.77e5
Max = 1.28e7
F
HCCLM3
3xflag
3xflag-CYB5A
Lv-Vector
Lv-STOML2
Surface
Bottom
HE
G
HCCLM3
3xflag
3xflag-CYB5A
Lv-Vector
Lv-STOML2
Luminescence
x10^5
Radiance (p/sec/cm²/sr)
Color Scale
Min = 6.01e4
Max = 2.68e5
H
HCCLM3
SP
HE
3xflag
3xflag-CYB5A
Lv-Vector
Lv-STOML2

S Figure 5-3

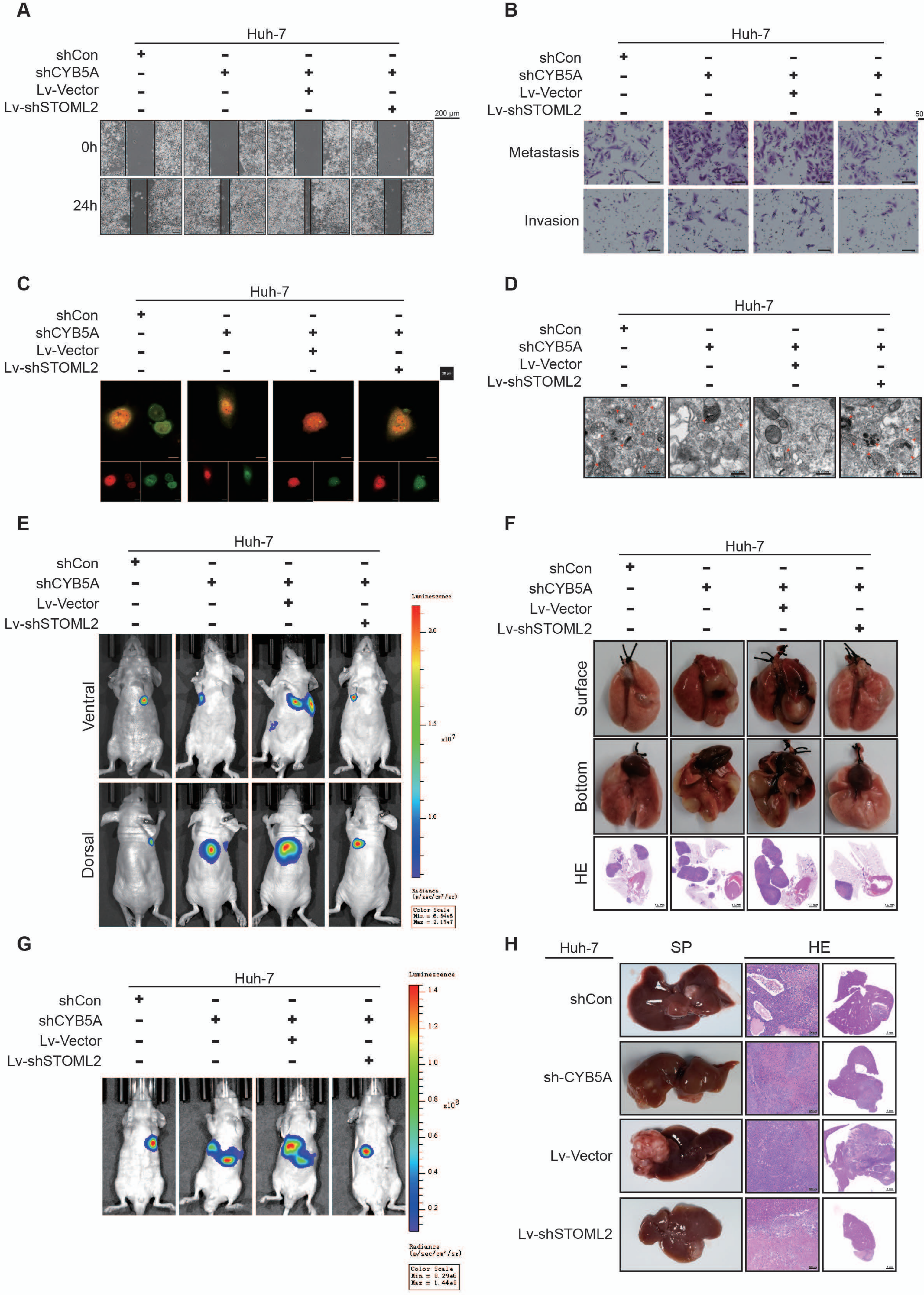

A
Huh-7
shCon
shCYB5A
Lv-Vector
Lv-shSTOML2
200 μm
0h
24h
B
Huh-7
shCon
shCYB5A
Lv-Vector
Lv-shSTOML2
50μm
Metastasis
Invasion
C
Huh-7
shCon
shCYB5A
Lv-Vector
Lv-shSTOML2
D
Huh-7
shCon
shCYB5A
Lv-Vector
Lv-shSTOML2
E
Huh-7
shCon
shCYB5A
Lv-Vector
Lv-shSTOML2
Ventral
Dorsal
Luminescence
2.0
1.5
1.0
x10^7
Radiance (p/sec/cm²/sr)
Color Scale Min = 6.84e6 Max = 2.15e7
F
Huh-7
shCon
shCYB5A
Lv-Vector
Lv-shSTOML2
Surface
Bottom
HE
G
Huh-7
shCon
shCYB5A
Lv-Vector
Lv-shSTOML2
Luminescence
1.4
1.2
1.0
0.8
0.6
0.4
0.2
x10^8
Radiance (p/sec/cm²/sr)
Color Scale Min = 8.29e6 Max = 1.44e8
H
Huh-7
SP
HE
shCon
sh-CYB5A
Lv-Vector
Lv-shSTOML2

S Figure 5-4

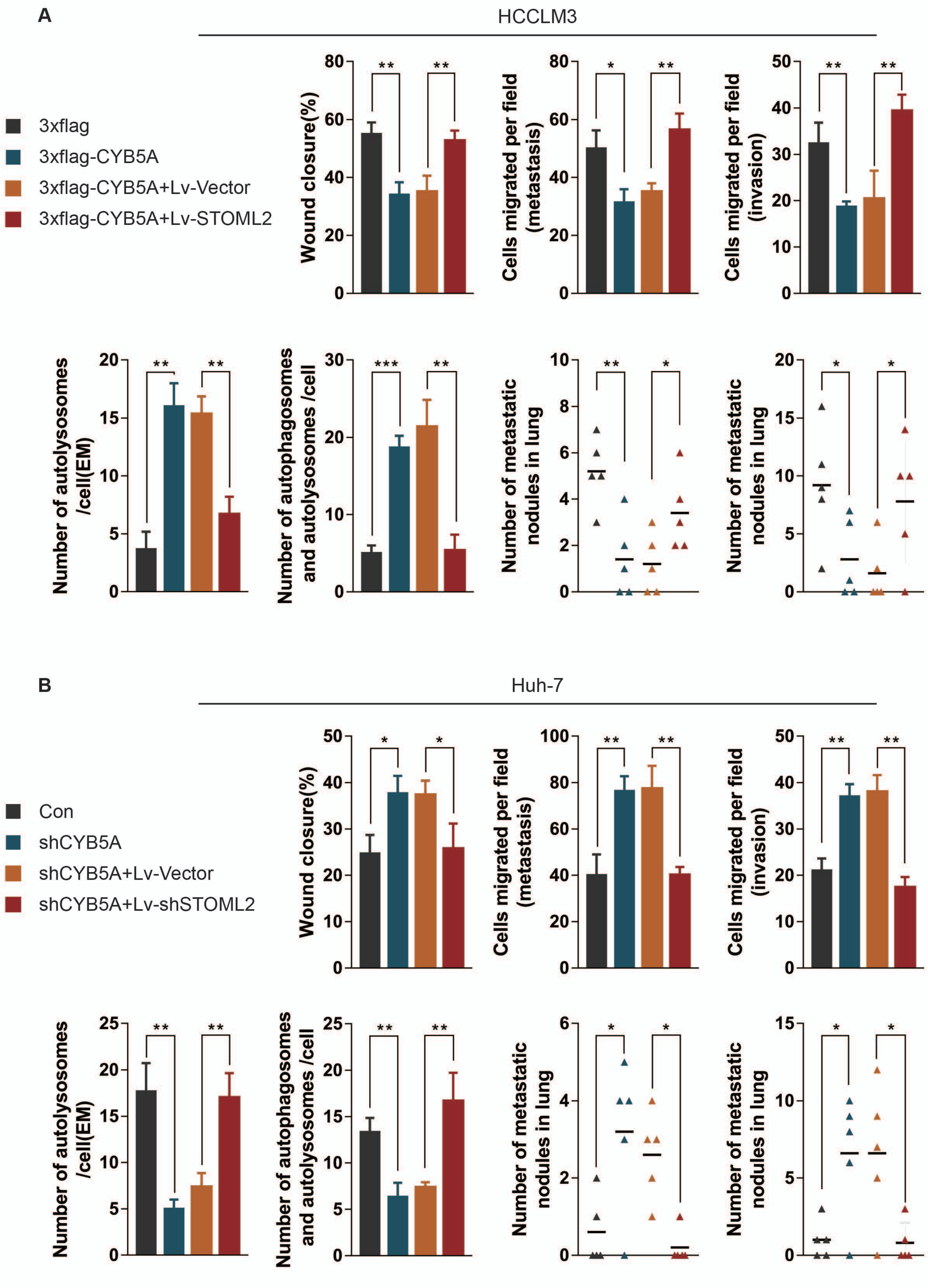
A
HCCLM3
3xflag
3xflag-CYB5A
3xflag-CYB5A+Lv-Vector
3xflag-CYB5A+Lv-STOML2
Wound closure(%)
Cells migrated per field (metastasis)
Cells migrated per field (invasion)
Number of autolysosomes /cell(EM)
Number of autophagosomes and autolysosomes /cell
Number of metastatic nodules in lung
B
Huh-7
Con
shCYB5A
shCYB5A+Lv-Vector
shCYB5A+Lv-shSTOML2
Wound closure(%)
Cells migrated per field (metastasis)
Cells migrated per field (invasion)
Number of autolysosomes /cell(EM)
Number of autophagosomes and autolysosomes /cell
Number of metastatic nodules in lung

S Figure 6-1

A

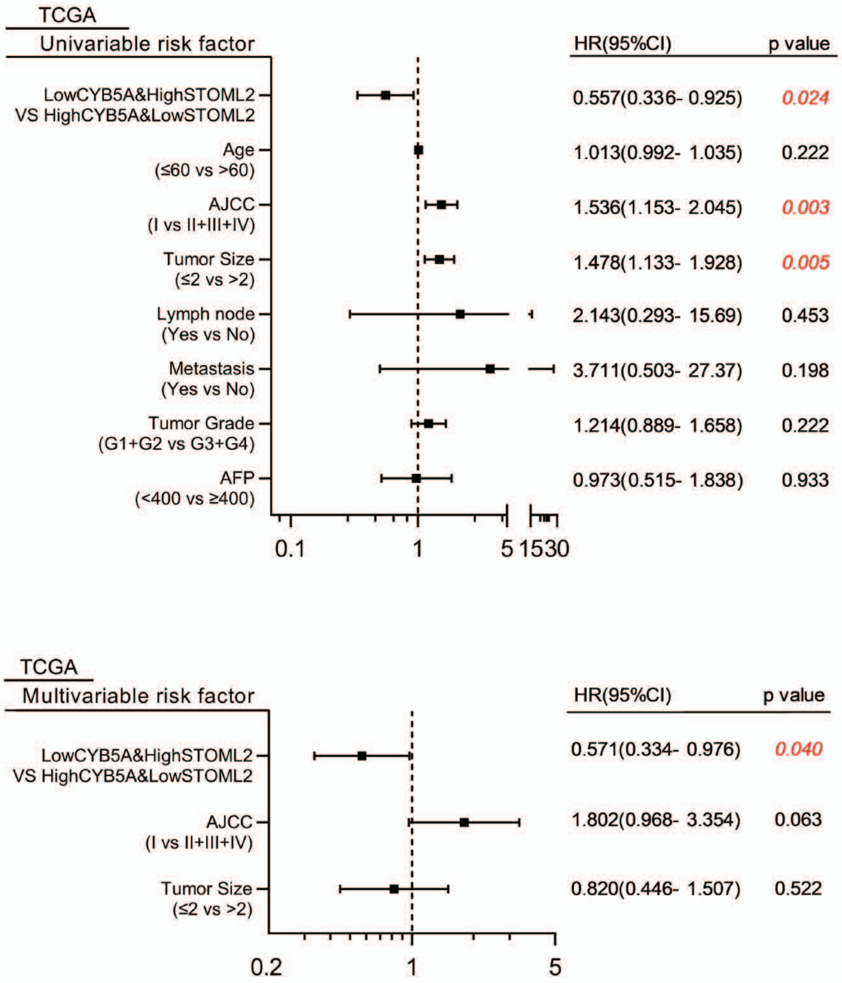

B

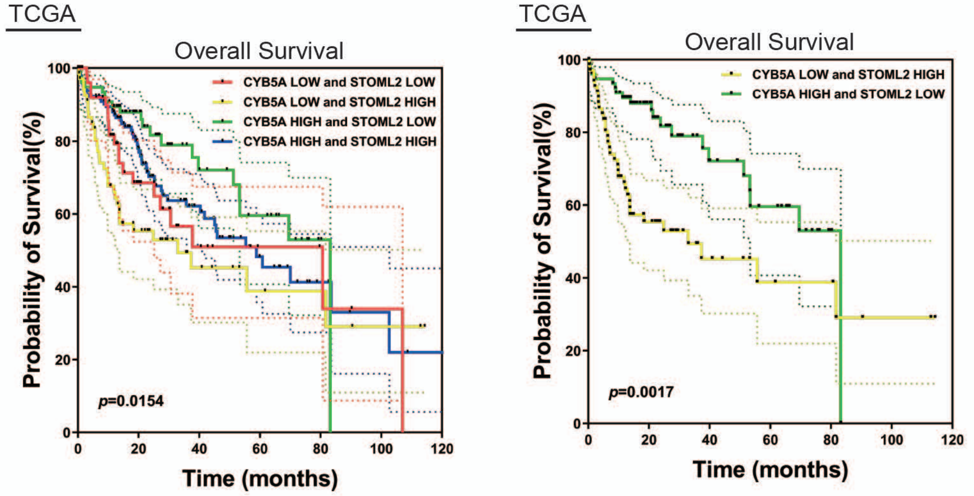

Supplement: Supplementary file 1 — Supplemental Figures [file 41419_2022_5053_MOESM1_ESM.pdf]
