# Supplementary material for: Cytochrome B5 type A alleviates HCC metastasis via regulating STOML2 related autophagy and promoting sensitivity to ruxolitinib
Source: Cell Death Dis. 2022 Jul 18;13(7):623. doi: 10.1038/s41419-022-05053-8 (PMC9293983; doi:10.1038/s41419-022-05053-8)
Supplement: Supplementary file 2 — Supplemental Figure legends [file 41419_2022_5053_MOESM2_ESM.docx]

**S FIGURE LEGENDS**

**S figure 1-1.** (A) Expression of CYB5A in normal human tissues. (B) Difference of CYB5A expression between tumors and adjacent non-cancerous in patients with different cancers. (C) Difference of CYB5A expression between HCC and adjacent normal tissues in patients from GEO database. (D) Western blot analyses of SARDH, and STARD5 protein expression in HCC tissues and adjacent normal tissues. T: tumor tissues; P: adjacent non-cancerous tissues.

**S Figure 1-2.** (A-F) The Kaplan-Meier analysis of OS and DFS in TCGA, GEO and HCCDB HCC database with different expression of CYB5A. (G-J) Univariate analyses were performed in the TCGA HCC cohort and clinical cohort (n=80). The bar correspond to 95% confidence intervals. Multivariate analyses were performed in the TCGA HCC cohort and clinical cohort (n=80). The bar correspond to 95% confidence intervals.

**S Figure 2-1.** (A-D) CYB5A expression was examined by qRT-PCR and western blot after Lenti-3xflag-CYB5A or Lenti-shCYB5A transfection in indicated HCC cell lines. (E) Effect of CYB5A on different loading control.

**S Figure 2-2.** (A) Wound healing assays for indicated cell lines. Scale bar: 200μm. (B) Invasion and migration transwell assays for indicated cell lines. Scale bar: 50μm. (C-D) Representative images of pulmonary metastases (n=10). (E) Representative images of bone and brain metastases (n=10).

**S Figure 2-3.** (A-G) Statistics for all experiments involved in **Figure 2** are in this figure.

**S Figure 2-4.** (A-B) Proliferation rate was analyzed by CCK-8 assay of indicated HCC cells. (C-D) Representative images of colony formation assays are shown in the left panels; the number of foci was counted as shown in the right panels.

**S Figure 3-1.** (A-C) The different expression genes under hypoxia between HCCLM3-control and HCCLM3-CYB5A cell lines. The significant thresholds were |fold change| > 2 and p < 0.05. Significant different expression genes were filtered and enriched in GO TRRUST analysis and KEGG signaling pathway. (D-E) Images represented the typical transmission electron microscopy of indicated cell lines and statistical analysis of autophagolysosome (ASS) numbers. The red arrows indicate ASS. (F-G) Images represented the typically indicated cell lines that stably expressed mRFP-EGFP-LC3 fusion protein which were shown by confocal microscopic analysis and statistical analysis of autophagosome and ASS numbers. Scale bar: 20μm.

**S Figure 3-2.** (A) Images represented the typically indicated cells treated with rapamycin (100 μM,24h) that expressed mRFP-EGFP-LC3 fusion protein shown by confocal microscopic analysis. Scale bar:20μm. (B) Migration transwell assays for indicated cells. Scale bar:100μm.

**S Figure 3-3.** (A) The expression correlation of CYB5A and HIF1A was shown in the TCGA HCC database. (B) The correlation of CYB5A and HIF1A pathway was shown by GSEA analysis. (C-D) The correlation of CYB5A and winter or ragnum hypoxia store. (E) The cell morphology and survival state after 20 hours of hypoxia treatment. (F) The cell apoptosis rate after 24 hours of hypoxia treatment.

**S Figure 4-1.** (A) The image of phosphorylated protein content determination array. (B) Western blot was performed to detect phosphorylated protein expression in HCCLM3 cells exposed in hypoxia. (C)Western blot was performed to detect autophagy-related protein expression in Huh-7 cells. (D) Indicated Cells were exposed to IL-6 (20 ng/mL, 24 h). Whole-cell lysates were analyzed by Western blotting. (E) Western blot was performed to detect JAK1, JAK2, JAK3 and TYK2 expression in the indicated cells. (F) Indicated cells were exposed to Hypoxia (20 h). Whole-cell lysates were analyzed by Western blotting. (G) The changes in size and number of pulmonary metastases in the indicated HCC cell lines after STAT3 was rescued by lentivirus. STAT3 rescue experiments were also shown in S Figure 4-2. (H) A simplified logical diagram of the preceding experiment (starting at the red arrow).

**S Figure 4-2.** Typical images and statistical analyses of STAT3 rescue experiments.

**S Figure 5-1.** (A) IF demonstrating the colocalization of CYB5A and STOML2 in HCC cells. (B) Typical images of STOML2 rescue western blot experiments. (C) The STOML2, STAT3, MMP9, BCL2, and HIF1A mRNA expression in Huh-7 and HCCLM3 cells was demonstrated by qRT-PCR. (D) Correlations among CYB5A, STAT3, MMP9 and HIF1A mRNA levels in human HCC tissues (TCGA HCC database).

**S Figure 5-2.** Typical images of STOML2 rescue experiments in HCCLM3 cell lines.

**S Figure 5-3.** Typical images of STOML2 rescue experiments in Huh-7 cell lines.

**S Figure 5-4.** Statistical analyses of STOML2 rescue experiments in HCCLM3 and Huh-7 cell lines.

**S Figure 6-1.** (A) Univariate and Multivariate analysis was performed in the TCGA database. The bar correspond to 95% confidence intervals. (B) OS in the TCGA database.
