# Supplementary material for: Cytochrome B5 type A alleviates HCC metastasis via regulating STOML2 related autophagy and promoting sensitivity to ruxolitinib
Source: Cell Death Dis. 2022 Jul 18;13(7):623. doi: 10.1038/s41419-022-05053-8 (PMC9293983; doi:10.1038/s41419-022-05053-8)
Supplement: Supplementary file 3 — Supplemental Materials and Methods [file 41419_2022_5053_MOESM3_ESM.docx]

**S Materials and Methods**

**Antibody**

ATG12 CST, #2011; ATG16L1 CST, #8089; ATG3 CST, #3415; ATG5 CST, #12994; ATG7 CST, #8858; Bcl-2 CST, #15071; CYB5A abcam, ab70350; CYB5A Proteintech, 12365-1-AP; DYKDDDK CST, #14793; DYKDDDK CST, #8146; GAPDH CST, 5174; HIF-1α CST, #36169; JAK1 CST, 3344; JAK2 CST, #3230; JAK3 CST, #8827; LC3B abcam, ab192890; MMP-9 abcam, ab76003; p-AKT CST, #4060; p-AKT CST, #13038; p-AMPK CST, #2535; p-Beclin-1 CST, #54101; p-Beclin-1 CST, #14717; p-Foxo1/3 CST, #9464; p-GSK-3β CST, #9323; p-JAK CST, #74129; p-mTOR CST, #2974; p-mTOR CST, #5536; p-p53 CST, #9288; p-p53 CST, #4030; p-Smad3 CST, #9520; p-Stat3 CST, #9145; p-ULK1 CST, #14202; p-β-Catenin CST, #4176; SARDH Proteintech, 22762-1-AP; STARD5 Proteintech,10487-1-AP; Stat3 CST, #9139; STOML2 abcam, ab191883; STOML2 Proteintech,60052-1-Ig; TYK2 CST, #14193; Ubiquitin CST, #3936; Vinculin CST, #13901; α/β-Tubulin CST, #2148; Actin Sigma, A2228

**Bioinformatics analysis**

RNA-seq original count data and clinical information of 360 cases of HCC cohort were downloaded from TCGA Database. Differential gene analysis (|log2 Fold Change|>1 & adjusted P value <0.05 identified as differential genes) and survival analysis (P value<0.05 is considered to have survival significance) were performed by applying Rstudio (version 3.6.3) and R packages (DESeq2, survival). The common parts of differential genes, survival prognostic significance and metabolic genes were selected to determine the gene to be studied and draw a heat map.

**Lentivirus and construction of stable cell line**

Lentiviral vectors for gene overexpression and knockdown (3xflag-CYB5A, shCYB5A， STOML2, STAT3, shSTOML2 and shSTAT3)) was constructed by GeneChem Corporation (Shanghai, China). The empty vectors (Lv-Con and Lv-shCon) were used as negative control. Puromycin was then added to the medium to select stable cell lines.

**Cell proliferation and colony formation assay**

In 96-well plates, cells were seeded at 1-1.5× 10^3^/well. Cell viability at various time points was determined using CCK-8 assays. In 6-well plates, cells were seeded at 1-1.5× 10^3^/well. The culturing medium was removed after 1-2 weeks of incubation, and the colonies were counted.

**Wound healing assay**

After seeding cells into six-well plates, cells were cultured until confluent. PBS was then used three times to wash the cells. In each well, a 10 μL pipette tip was used to scratch the bottom. The wound healing was detected at 0 h, 12 h and 24 h.

**Migration and invasion assays**

Invasion and migration assays were carried out using a 24-well insert system. 500 μL 0% FBS medium were mixed with 1-5 × 10^4^ cells, seeded in the upper chambers. Next, we added 700 μL 10-20% FBS medium to the lower chambers. As part of the Matrigel invasion assay, Matrigel 39 μL was precoated on the filters. Incubation for 24 hours, we fixed the cells on the lower surface of the filter membrane.

**Patients and tissue samples**

80 HCCs and their adjacent non-cancerous tissue were acquired during routine surgeries between 2013 and 2018 at the First Affiliated Hospital of Harbin Medical University (Harbin, China). According to WHO guidelines, the histopathology diagnosis. A written informed consent form from each patient was obtained following ethical approval by The First Affiliated Hospital of Harbin Medical University Research Ethics Committee.

**Cell lines and cell culture**

Hep-G2, SNU-387, HEP-3B, PLC/PRF/5 cell lines were purchased from ATCC. Huh-7 cell line were purchased from JCRB Cell Bank. HCCLM3 was purchased from the Institute of Biochemistry and Cell Biology. All cell lines were STR tested for identity annually. And mycoplasma was tested every 6 mounths. Culture of all cell lines was performed in DMEM or RPMI 1640 supplemented with 10% FBS and 1% antibiotics (100 U/mL penicillin and 100 μg/mL streptomycin) at 37 °C in a 5% CO_2_ incubator.

**Animal studies**

Male BALB/c athymic nude mice (4–6-week-old) were obtained from the Shanghai SLAC Laboratory Animal Company Limited for Biological Sciences and were reared in SPF Level Animal Experiment Center, West Campus of College of Life Sciences, USTC. The animal experiments of this subject have been approved by the Laboratory Animal Ethics Committee of USTC. In addition, all animals were operated in accordance with the regulations of USTC's Laboratory Animal Center.

During *in vivo* assays, intrahepatic, pulmonary, and skeletal metastases were observed. All mice were randomly grouped before any experiments started.1.5 x10^6^ HCC cells were injected in 100 μL of PBS into the spleen parenchyma, the left heart ventricle, or the tail vein. Following 4-6 weeks, the mice were euthanized and the tissues were harvested.

In order to minimize the pain of the experiment to the mice，all xenograft mice will be sacrificed if they meet any of the following conditions: 1. Reach the date indicated. 2. Estimate the Maximum diameter of tumor is about to reach 1.5cm. 3. Estimate the tumor is about to reach 10% of body weight.

**Statistical analysis**

The results of all cell culture and biochemical tests were repeatable three times without ambiguity. To determine whether there were differences between the different experimental groups, we performed statistical analysis using the program GraphPad Prism. Figure legends include statistical parameters, with 0.05 being considered statistically significant.

**Western blot**

Lysates of either cells or tissues were treated with RIPA buffer (Biotime, P0013) containing protease and phosphatase inhibitors. We measured the protein concentrations in the samples, separated them with SDS-PAGE, and subsequently transferred them onto PVDF membranes (Invitrogen, Carlsbad, CA, USA). Membranes were blocked with skim milk or BSA.Incubation with secondary antibodies was preceded by an incubation with primary antibodies. Thermo Fisher Scientific, Waltham, MA, USA, provided an enhanced chemiluminescence kit for detection of immunoreactive bands.

**Co-IP assay**

After incubating the cells with IP lysis buffer per well on ice, a protease and phosphatase inhibitor cocktail (Thermo Scientific) of 10 mL/mL was added to each well. A microcentrifuge tube was then used to transfer the lysate. Then，we immunoprecipitated the Dynabeads®-Ab-Ag complex using a Dynabeads^TM^ protein G IP kit and Magnet Starter Pack from Thermo Scientific.

**RNA Extraction and Quantitative RT-PCR**

Total RNA was extracted from cells using RNeasy Mini kit (Qiagen). Then, PrimeScript^TM^ RT reagent kit (TaKaRa) was used to synthesize cDNA. The cDNA was amplified by SYBR Premix Ex Taq Ⅱ reagent kit (TaKaRa) and ABI Stepone Plus instrument (Applied Biosystems) . The primers listed in Supplementary data 7 and normalized to ACTB.

**Immunohistochemical (IHC) staining**

After formalin-fixed and paraffin-embedded, following antibody incubation, sections were stained with Vector Laboratories' DAB kit and counterstained with Sigma-Aldrich's hematoxylin. Scoring was based on stain intensity and extent.

**Immunofluorescence (IF) assay**

For the IF assay, cells were fixed, permeabilized with 0.5% Triton X-100 , and blocked in 10% goat serum，then incubated with primary antibodies. The cells were incubated with fluorescent secondary antibodies (Invitrogen, Eugen) at next morning. Finally, the cells were counterstained with DAPI (Vector Laboratories).

**Flow cytometry analysis of apoptotic cells**

Annexin V-FITC Apoptosis Detection Kit (Beyotime) was used to determine the percentage apoptotic cells. Cells were analyzed on a BD LSRFortessa flow cytometer.

**Tartrate-resistant acid phosphatase (TRAP)**

TRAP staining was performed to identify and quantify osteoclast-like cells using the TRAP Kit (Sigma-Aldrich) in accordance with the manufacturer's instructions.

**Micro-computed tomography (Micro-CT) scan**

The bioluminescence-suspected bones were scanned using a Quantum GX micro-CT Imaging System (PerkinElmer, Inc. Boston, MA, USA). Three-dimensional images were reconstructed and analyzed for the visualization of osteolytic or osteoblastic lesions.

**Protein microarray（Full Moon Microsystems）**

Performing in accordance with the chip inspection standard process provided by Full Moon. The chip is blocked and incubated. Then using Agilent SureScan Dx Microarray Scanner chip scanner to scan chip.

**Bioluminescence assay**

Bioluminescence (BLI) assay was performed using a cryogenically cooled imaging system (IVIS spectrum, Perkin Elmer, USA). Mice were injected with a fresh stock solution of D-Luciferin, Firefly, potassium salt in PBS (150 mg/kg body weight, PerkinElmer, Inc. Boston, MA, USA) intra-peritoneally. Then in vivo imaging was performed 10-15 min after injection. And then imaged. Images of the animals were acquired using Living Image software (Xenogen, Corporation, Alameda, CA) by measuring photon flux. Pseudo-color overlays on gray scale images indicated the distribution of photons. And the BLI signal intensity was quantified as the sum of photons within region of interest (ROI).

**Transmission electron microscopy**

Fixation of cells in glutaraldehyde containing 0.1 mol/L sodium cacodylate at 2.5%. Then fixed using 1% osmiumtetr oxid. Afterwards, dehydration. The embedding was followed by cutting the samples into 50-nm sections and staining them with 3 % uranyl acetate and lead citrate. Images were acquired by using a 80kV electron microscope.
