# Supplementary material for: Cytochrome B5 type A alleviates HCC metastasis via regulating STOML2 related autophagy and promoting sensitivity to ruxolitinib
Source: Cell Death Dis. 2022 Jul 18;13(7):623. doi: 10.1038/s41419-022-05053-8 (PMC9293983; doi:10.1038/s41419-022-05053-8)
Supplement: Supplementary file 5 — Supplementary data 2 [file 41419_2022_5053_MOESM5_ESM.doc]

|  | **CYB5A gene expression** | |  |
| --- | --- | --- | --- |
|  | **Low expressionn** | **High expressionn** | ***p* value** |
| **Age(years)** |  |  |  |
| ≤60 | 95 | 77 | 0.0509 |
| >60 | 84 | 103 |  |
| **Gender** |  |  |  |
| Male | 104 | 139 | ***0.0001*** |
| Female | 75 | 41 |  |
| **AJCC** |  |  |  |
| I | 70 | 98 | ***0.0033*** |
| II+III+IV | 98 | 72 |  |
| **Tumor Size** |  |  |  |
| ≤2 | 127 | 143 | 0.0905 |
| >2 | 50 | 37 |  |
| **Lymph node** |  |  |  |
| Yes | 3 | 0 | 0.0826 |
| No | 122 | 124 |  |
| **Metastasis** |  |  |  |
| Yes | 2 | 2 | 0.9938 |
| No | 129 | 128 |  |
| **Tumor Grade** |  |  |  |
| G1+G2 | 87 | 137 | ***<0.0001*** |
| G3+G4 | 89 | 41 |  |
| **AFP** |  |  |  |
| ≤400 | 85 | 122 | ***<0.0001*** |
| >400 | 49 | 15 |  |

S Table 2 Clinic characteristic
