# Supplementary material for: Cytochrome B5 type A alleviates HCC metastasis via regulating STOML2 related autophagy and promoting sensitivity to ruxolitinib
Source: Cell Death Dis. 2022 Jul 18;13(7):623. doi: 10.1038/s41419-022-05053-8 (PMC9293983; doi:10.1038/s41419-022-05053-8)
Supplement: Supplementary file 10 — Supplementary data 7 [file 41419_2022_5053_MOESM10_ESM.doc]

1. Primer RNA list

| Name | Species | Forward primer sequence | Reversed primer sequence |
| --- | --- | --- | --- |
| CYB5A | Ho | CACCACAAGGTGTACGATTTGA | CATCTGTAGAGTGCCCGACAT |
| ACTB | Ho | CTCGCCTTTGCCGATCC | GAATCCTTCTGACCCATGCC |
| GAPDH | Ho | GAGAAGTATGACAACAGCCTCAA | GCCATCACGCCACAGTTT |
| STOML2 | Ho | AGGGGCTCTCTACTGGCTTC | ATCCGGTCTAACACAGGGATG |
| VCL | Ho | CTCGTCCGGGTTGGAAAAGAG | AGTAAGGGTCTGACTGAAGCAT |
| MMP9 | Ho | TGTACCGCTATGGTTACACTCG | GGCAGGGACAGTTGCTTCT |
| MAP1LC3B | Ho | AAGGCGCTTACAGCTCAATG | CTGGGAGGCATAGACCATGT |
| STAT3 | Ho | CAGCAGCTTGACACACGGTA | AAACACCAAAGTGGCATGTGA |
| BCL2 | Ho | GGTGGGGTCATGTGTGTGG | CGGTTCAGGTACTCAGTCATCC |
| HIF1A | Ho | GAACGTCGAAAAGAAAAGTCTCG | CCTTATCAAGATGCGAACTCACA |

1. Short hairpin (Sh) RNA sequence

| Name | Sequence |
| --- | --- |
| Sh-CYB5A-1 | CCGGGCTACTGAGAACTTTGAGGATCTCGAGATCCTCAAAGTTCTCAGTAGCTTTTTG |
| Sh-CYB5A-2 | CCGGCCATCCAGATGACAGACCAAACTCGAGTTTGGTCTGTCATCTGGATGGTTTTTG |
| Sh-CYB5A-3 | CCGGGCACCACAAGGTGTACGATTTCTCGAGAAATCGTACACCTTGTGGTGCTTTTTG |
| Sh-STAT3-1 | CCGGGCTGACCAACAATCCCAAGAACTCGAGTTCTTGGGATTGTTGGTCAGCTTTTT |
| Sh-STAT3-2 | CCGGGCACAATCTACGAAGAATCAACTCGAGTTGATTCTTCGTAGATTGTGCTTTTT |
| Sh-STAT3-3 | CCGGGCAAAGAATCACATGCCACTTCTCGAGAAGTGGCATGTGATTCTTTGCTTTTT |
| Sh-STOML2-1 | CCGGCCGTTATGAGATCAAGGATATCTCGAGATATCCTTGATCTCATAACGGTTTTTTG |
| Sh-STOML2-2 | CCGGCGACAATGTAACTCTGCAAATCTCGAGATTTGCAGAGTTACATTGTCGTTTTTTG |
| Sh-STOML2-3 | CCGGGCAAATCGATGGAGTCCTTTACTCGAGTAAAGGACTCCATCGATTTGCTTTTTTG |
