# Supplementary material for: Cytochrome B5 type A alleviates HCC metastasis via regulating STOML2 related autophagy and promoting sensitivity to ruxolitinib
Source: Cell Death Dis. 2022 Jul 18;13(7):623. doi: 10.1038/s41419-022-05053-8 (PMC9293983; doi:10.1038/s41419-022-05053-8)

Figure1 C

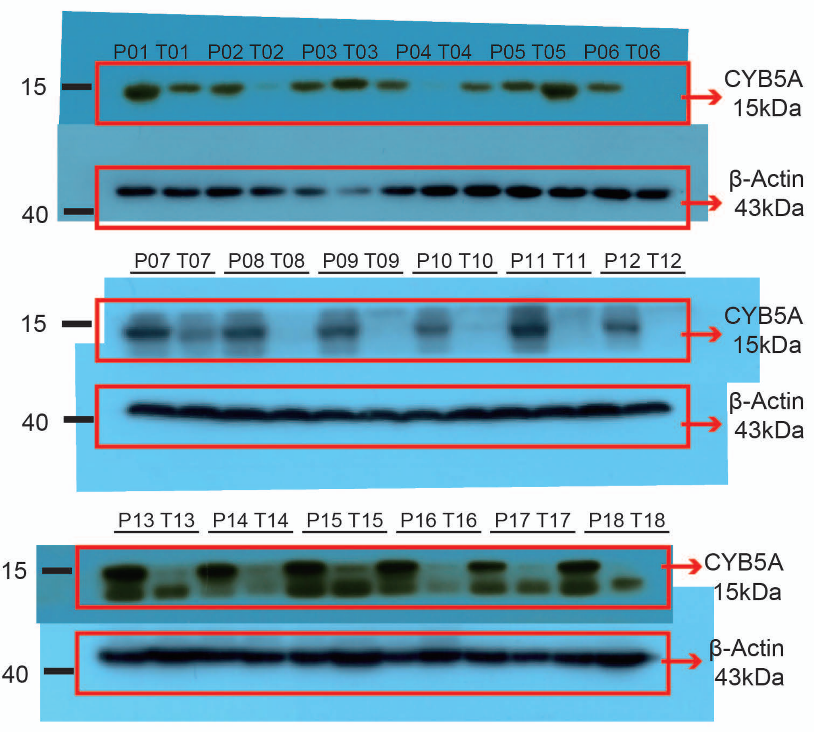

S Figure1 D

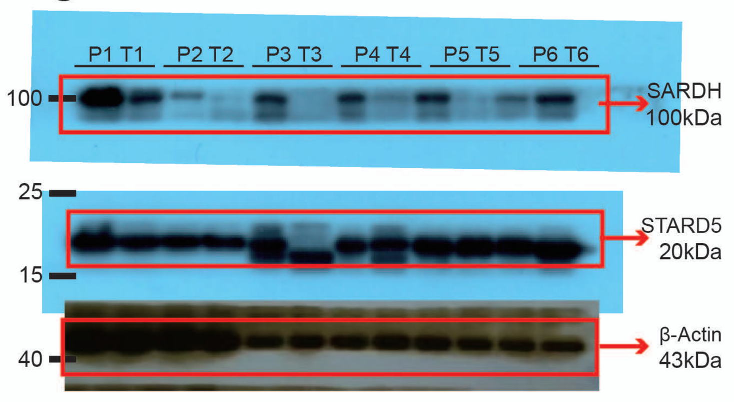

Figure1 H

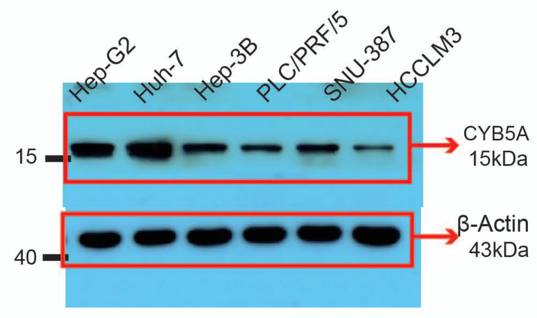

Figure3 D

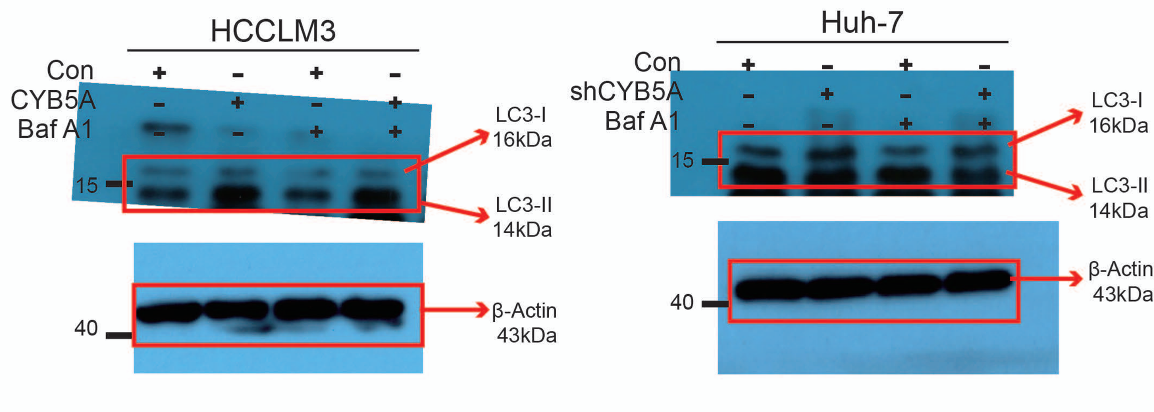

Figure4 B

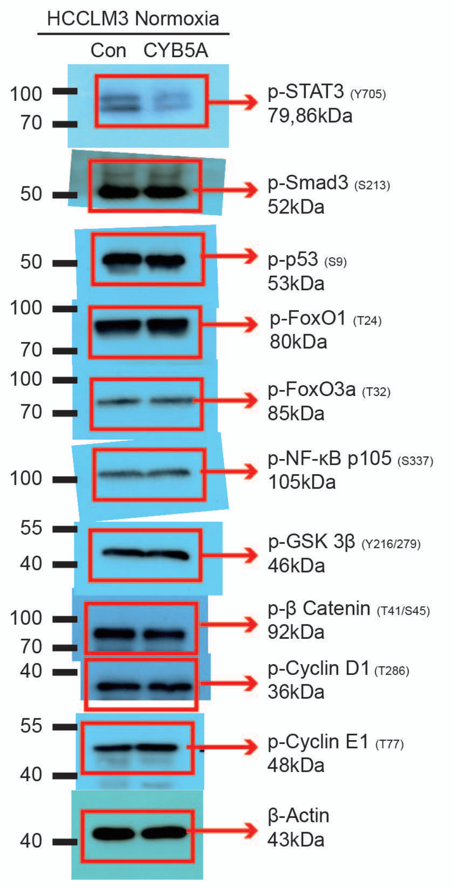

Figure4 C

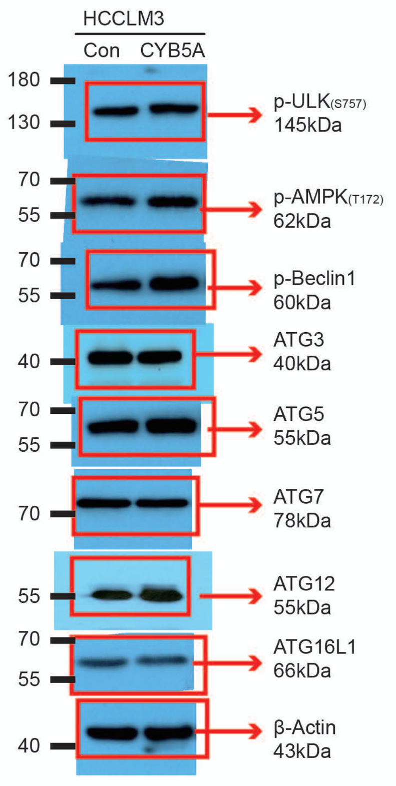

Figure4 D

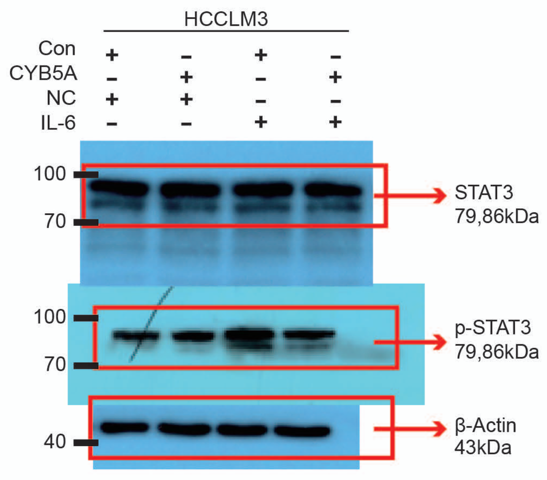

Figure4 E

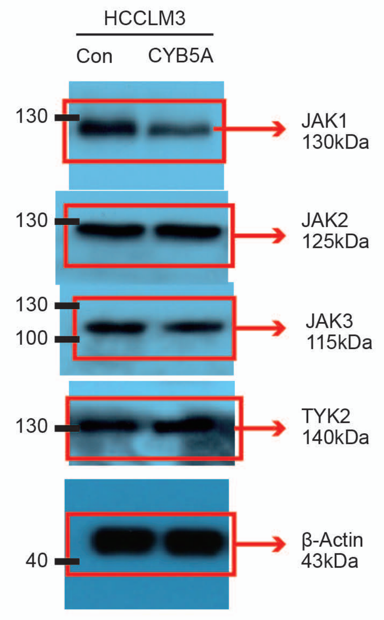

Figure4 F

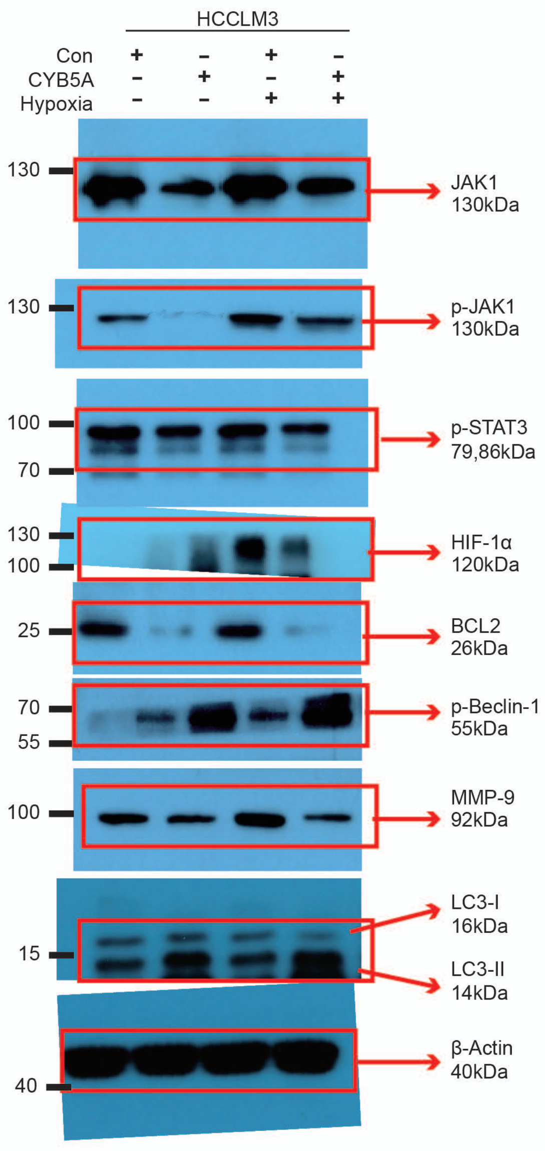

Figure5 E

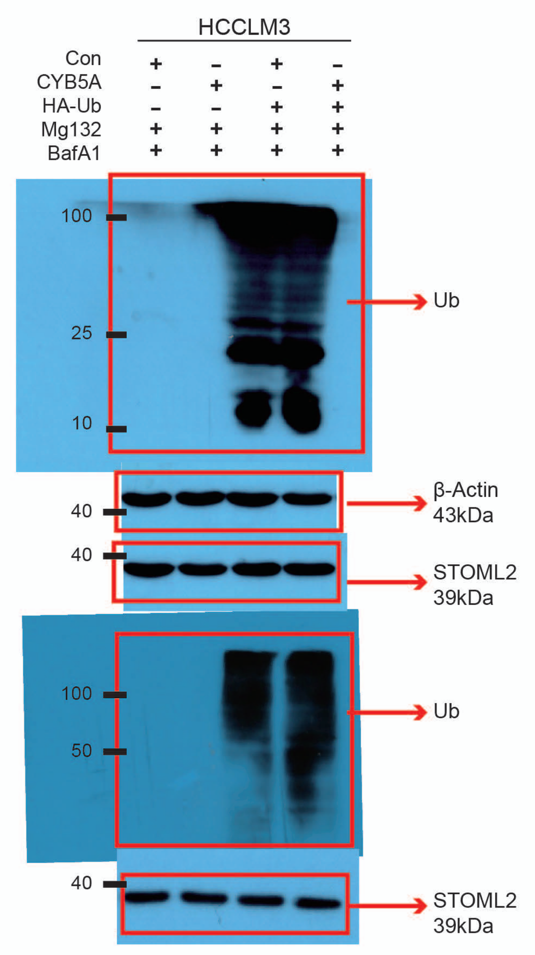

Figure5 B

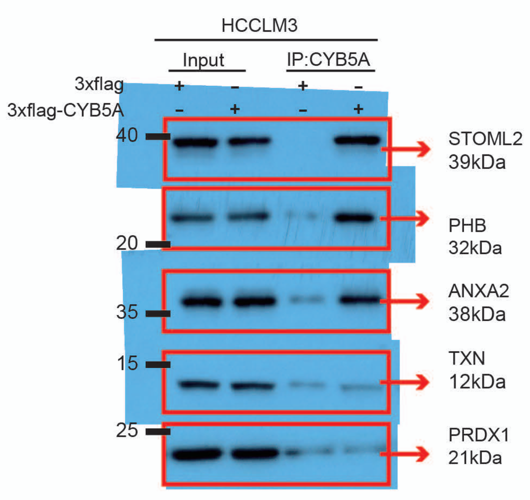

Figure5 C

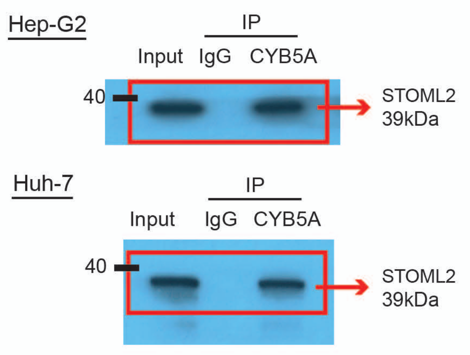

S Figure2-1 A

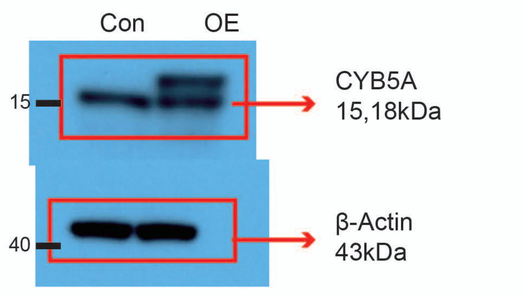

S Figure2-1 B

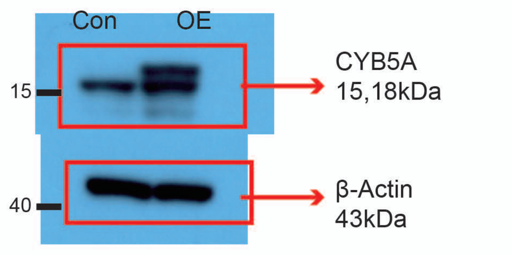

S Figure2-1 C

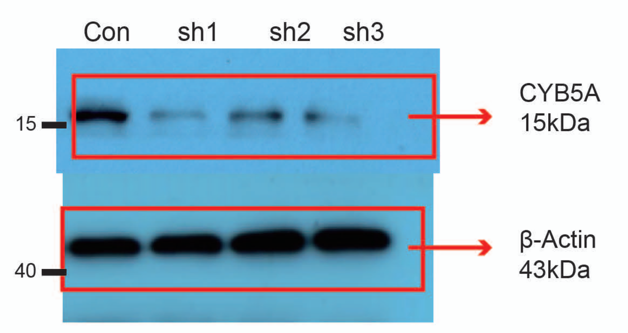

S Figure2-1 D

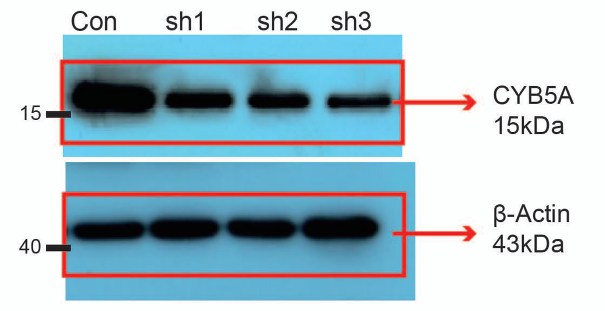

S Figure2-1 E

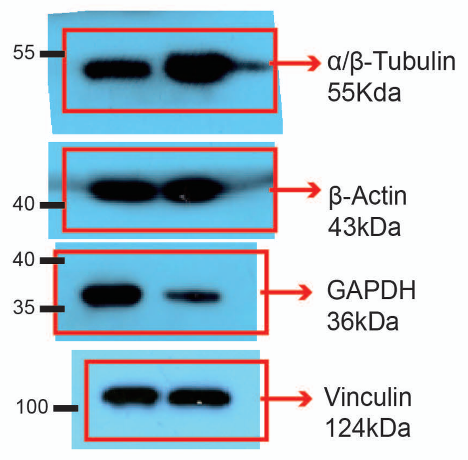

Figure5 G

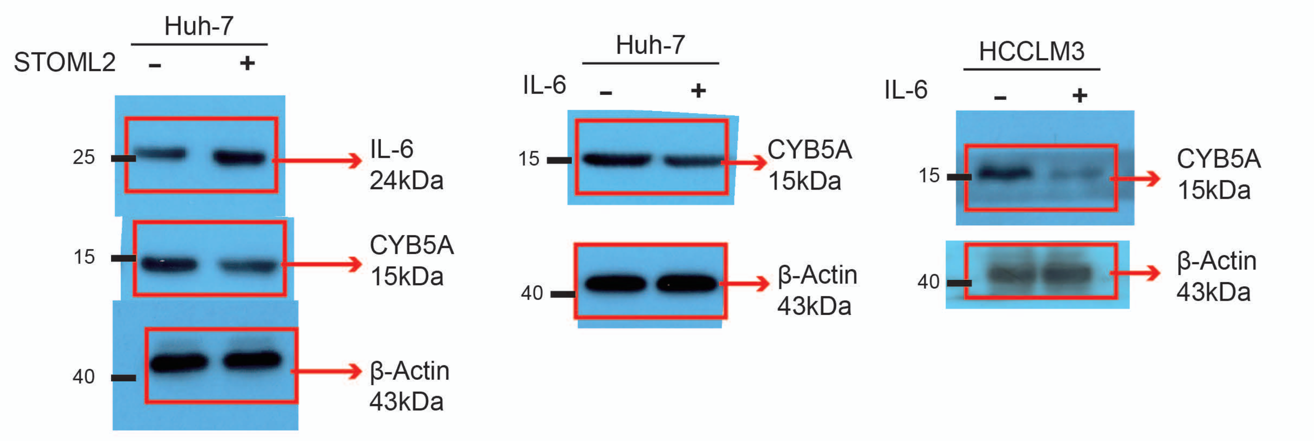

S Figure4-1 B

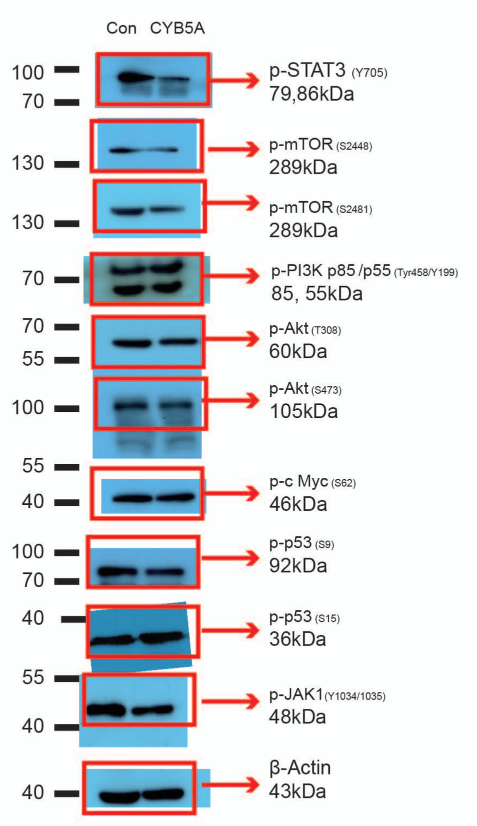

S Figure4-1 C

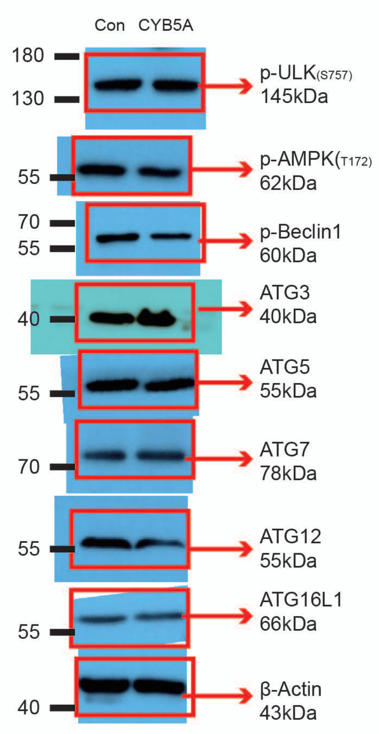

S Figure4-1 D

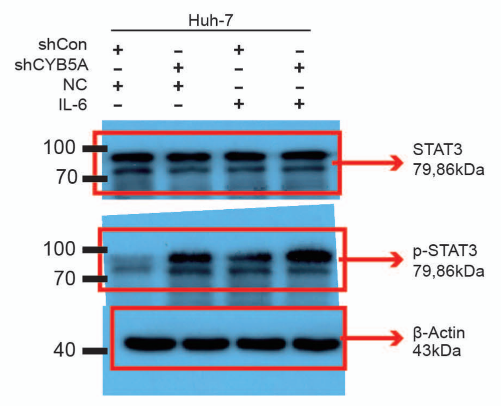

S Figure4-1 E

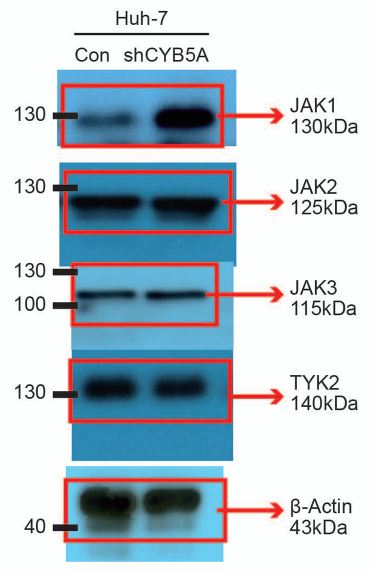

S Figure4-1 F

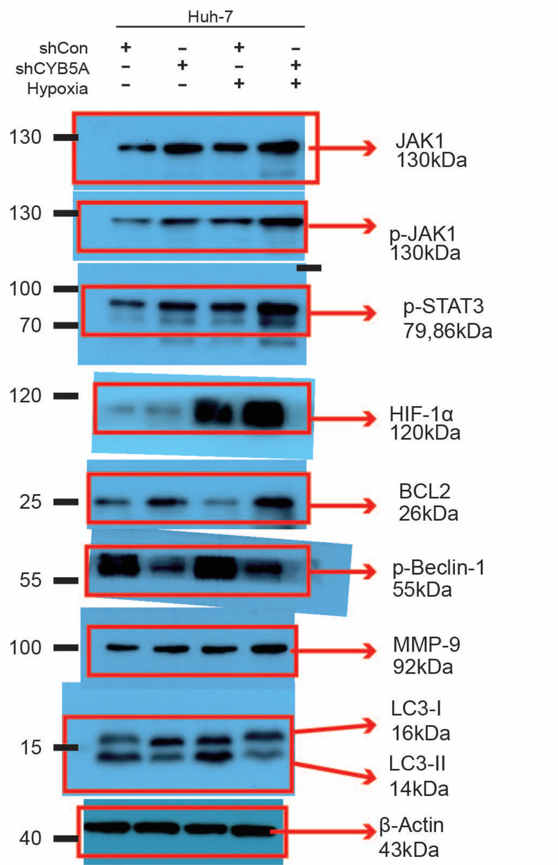

S Figure4-2 E

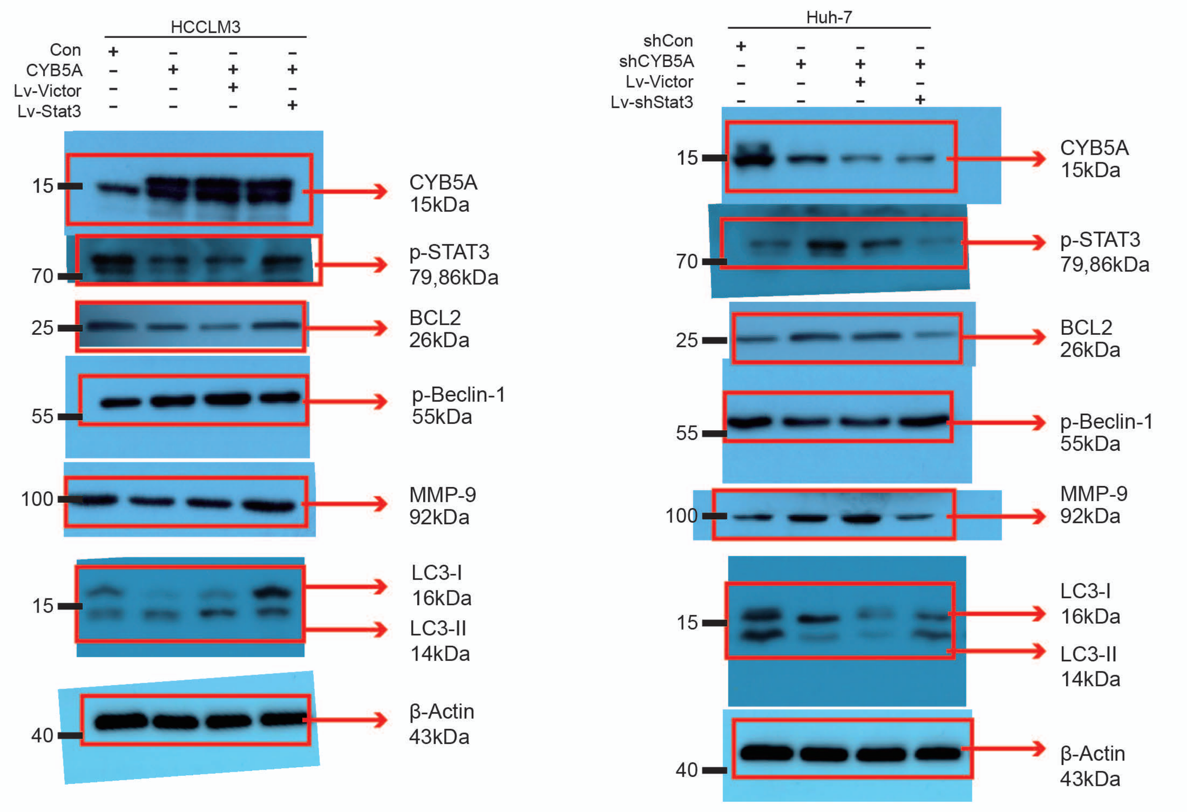

S Figure5-1 B

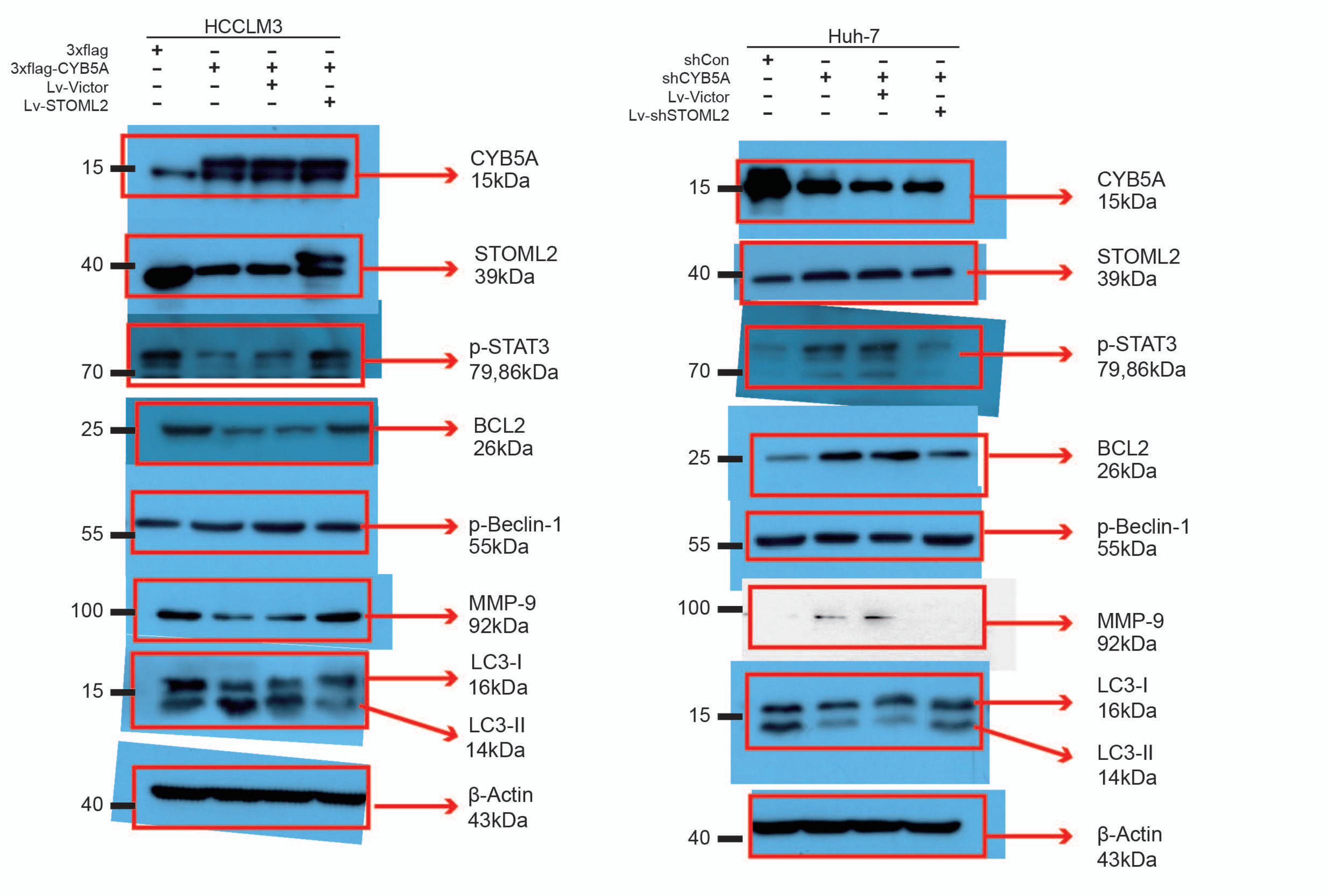

Supplement: Supplementary file 11 — Original Data File [file 41419_2022_5053_MOESM11_ESM.pdf]
