# Supplementary material for: Cytochrome B5 type A alleviates HCC metastasis via regulating STOML2 related autophagy and promoting sensitivity to ruxolitinib
Source: Cell Death Dis. 2022 Jul 18;13(7):623. doi: 10.1038/s41419-022-05053-8 (PMC9293983; doi:10.1038/s41419-022-05053-8)
Supplement: Supplementary file 13 — Co-author information changes statement [file 41419_2022_5053_MOESM13_ESM.pdf]

主　题:	Re:The change of co-authors about CDDIS-21-4379RR
发件人:	"郭泓瑞" <hongrui7456@163.com> 2022-6-27 14:00:36
收件人:	liulx@ustc.edu.cn

Dear Professor Liu：

I accept all the author information changes.

Sincerely yours，
Hongrui Guo

At 2022-06-27 11:48:19, liulx@ustc.edu.cn wrote:

> **Dear Authors：**
>
> Our recent manuscript CDDIS-21-4379RR, entitled "Cytochrome B5 Type A alleviates HCC metastasis via regulating STOML2 related autophagy and promoting sensitivity to Ruxolitinib." has been provisionally accepted for publication in Cell Death & Disease, lists you as a co-author. The author information has changed since the original submission.
>
> In particular, I added Jizhou Wang as a co-author in view of his important contribution to the revision process. And I adjusted the order considering the contributions of all authors.
>
> **As the author information has changed, I ask for your opinion:**
>
> If you **agree** with all the final author information, please reply **I accept all the author information changes.**
>
> If you **disagree** with the final author information or have other suggestions, please email me with details.
>
> The changes of Author List and Contributions are as follows:

Final Author List:

Hongrui Guo[1*], Shuhang Liang[2*], Yan Wang[3*], Shuo Zhou[2*], Dalong Yin[2*], Shugeng Zhang[2], Jizhou Wang[2], Dehai Wu[1], Kun Ma[1], Yufeng Liu[1], Linmao Sun[1], Changyong Ji[2], Xianying Li[2], Huanran Zhou[2], Guangchao Yang[1], Xinyu Guo[1], Tianming Cui[2], Zihao Li[1], Yao Liu[2+], Jiabei Wang[2+], Lianxin Liu[1,4+]

Final Author affiliations:

1.Department of Hepatic Surgery, Key Laboratory of Hepatosplenic Surgery, Ministry of Education, The First Affiliated Hospital of Harbin Medical University, Harbin, China

2.Department of Hepatobiliary Surgery, Anhui Province Key Laboratory of Hepatopancreatobiliary Surgery, The First Affiliated Hospital of USTC, Division of Life Sciences and Medicine, University of Science and Technology of China, Hefei, Anhui, 230001, China

3.Department of Colorectal Surgery, Harbin Medical University Cancer Hospital, Harbin, 150001 Heilongjiang, China

4. Lead contact

* These authors contributed equally to this work.

Final Author contributions:

Conceptualization, G.HR., L.SH., L.LX., W.JB., L.Y.; Methodology, Y.DL., W.DH., W.Y., Z.S., W.JZ., Z.SG., M.K., L.ZH.; Software, L.YF., S.LM., J.CY., L.XY., Y.GC.; Validation, G.HR., L.SH, Z.HR., L.ZH., G.XY., C.TM.; Formal Analysis, Z.S.,Y.DL.,L.LX., W.JB., L.Y.; Investigation, G.HR., L.SH, L.LX., W.JB.; Writing - Original Draft, G.HR., Z.SG.; Writing - Review & Editing, Z.HR., L.LX., W.JB., L.Y.; Visualization, G.HR., L.YF., S.LM., J.CY., L.XY., Y.GC.; Funding Acquisition, W.Y., L.Y., W.JB., L.LX.; Resources, L.LX., W.JB., L.Y.; Supervision, L.LX., W.JB., L.Y..

Thank you very much for your time and consideration. Wish you have a nice day!

Sincerely yours，

Lianxin Liu

主　题:	回复: The change of co-authors about CDDIS-21-4379RR
发件人:	"我只输给天" <154652031@qq.com> 2022-6-27 12:54:19
收件人:	liulx <liulx@ustc.edu.cn>

Dear professor Liu:
Thank you for your email. I accept all the author information changes.

Sincerely yours,

Shuhang Liang

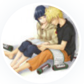

我只输给天
154652031@qq.com

------------------ 原始邮件 ------------------

发件人: "liulx" <liulx@ustc.edu.cn>;
发送时间: 2022年6月27日(星期一) 中午11:48
收件人: "郭泓瑞"<hongrui7456@163.com>;"我只输给天"<154652031@qq.com>;"wangyan2016md"<wangyan2016md@126.com>;"zhouwill01"<zhouwill01@163.com>;"尹大龙"<dalong48@163.com>;"张树庚"<shugeng517@163.com>;"王继洲"<wangjizhou1984@hotmail.com>;"tianxiadiyi_12345"<tianxiadiyi_12345@126.com>;"1694769800"<1694769800@qq.com>;"413951217"<413951217@qq.com>;"lemonzhutou"<lemonzhutou@163.com>;"cyji"<cyji@mail.ustc.edu.cn>;"lidonglps"<lidonglps@qq.com>;"drzhr1026"<drzhr1026@hotmail.com>;"ygc1988"<ygc1988@sina.com>;"1006065333"<1006065333@qq.com>;"1241346394"<1241346394@qq.com>;"1254007965"<1254007965@qq.com>;"刘尧"<liuyao66@ustc.edu.cn>;"jbwang16"<jbwang16@ustc.edu.cn>;
主题: The change of co-authors about CDDIS-21-4379RR

Dear Authors:

Our recent manuscript CDDIS-21-4379RR, entitled "Cytochrome B5 Type A alleviates HCC metastasis via regulating STOML2 related autophagy and promoting sensitivity to Ruxolitinib." has been provisionally accepted for publication in Cell Death & Disease, lists you as a co-author. The author information has changed since the original submission.

In particular, I added Jizhou Wang as a co-author in view of his important contribution to the revision process. And I adjusted the order conIsidering the contributions of all authors.

As the author information has changed, I ask for your opinion:

If you **agree** with all the final author information, please reply **I accept all the author information changes.**

If you **disagree** with the final author information or have other suggestions, please email me with details.

The changes of Author List and Contributions are as follows:

Final Author List:

Hongrui Guo[1*], Shuhang Liang[2*], Yan Wang[3*], Shuo Zhou[2*], Dalong Yin[2*], Shugeng Zhang[2], Jizhou Wang[2], Dehai Wu[1], Kun Ma[1], Yufeng Liu[1], Linmao Sun[1], Changyong Ji[2], Xianying Li[2], Huanran Zhou[2], Guangchao Yang[1], Xinyu Guo[1], Tianming Cui[2], Zihao Li[1], Yao Liu[2+], Jiabei Wang[2+], Lianxin Liu[1,4+]

Final Author affiliations:

1.Department of Hepatic Surgery, Key Laboratory of Hepatosplenic Surgery, Ministry of Education, The First Affiliated Hospital of Harbin Medical University, Harbin, China

2.Department of Hepatobiliary Surgery, Anhui Province Key Laboratory of Hepatopancreatobiliary Surgery, The First Affiliated Hospital of USTC, Division of Life Sciences and Medicine, University of Science and Technology of China, Hefei, Anhui, 230001, China

3.Department of Colorectal Surgery, Harbin Medical University Cancer Hospital, Harbin, 150001 Heilongjiang, China

4. Lead contact

* These authors contributed equally to this work.

Final Author contributions:

Conceptualization, G.HR., L.SH., L.LX., W.JB., L.Y.; Methodology, Y.DL., W.DH., W.Y., Z.S., W.JZ., Z.SG., M.K., L.ZH.; Software, L.YF., S.LM., J.CY., L.XY., Y.GC.; Validation, G.HR., L.SH, Z.HR., L.ZH., G.XY., C.TM.; Formal Analysis, Z.S.,Y.DL.,L.LX., W.JB., L.Y.; Investigation, G.HR., L.SH, L.LX., W.JB.; Writing - Original Draft, G.HR., Z.SG.; Writing - Review & Editing, Z.HR., L.LX., W.JB., L.Y.; Visualization, G.HR., L.YF., S.LM., J.CY., L.XY., Y.GC.; Funding Acquisition, W.Y., L.Y., W.JB., L.LX.; Resources, L.LX., W.JB., L.Y.; Supervision, L.LX., W.JB., L.Y..

Thank you very much for your time and consideration. Wish you have a nice day!

Sincerely yours,

Lianxin Liu

主　题:	回复：The change of co-authors about CDDIS-21-4379RR	
发件人:	"王岩" <wangyan2016md@126.com>	2022-6-27 13:15:29
收件人:	liulx <liulx@ustc.edu.cn>	

Dear Professor Lianxin Liu,
I accept all the author information changes.
Sincerely，Yan Wang

---- 回复的原邮件 ----

发件人	liulx@ustc.edu.cn<liulx@ustc.edu.cn>
日期	2022年06月27日 11:48
收件人	郭泓瑞<hongrui7456@163.com>、梁书航<154652031@qq.com>、wangyan2016md@126.com<wangyan2016md@126.com>、zhouwill01@163.com<zhouwill01@163.com>、尹大龙<dalong48@163.com>、张树庚<shugeng517@163.com>、王继洲<wangjizhou1984@hotmail.com>、tianxiadiyi_12345@126.com<tianxiadiyi_12345@126.com>、1694769800@qq.com<1694769800@qq.com>、413951217<413951217@qq.com>、lemonzhutou@163.com<lemonzhutou@163.com>、cyji@mail.ustc.edu.cn<cyji@mail.ustc.edu.cn>、lidonglps@qq.com<lidonglps@qq.com>、drzhr1026@hotmail.com<drzhr1026@hotmail.com>、ygc1988@sina.com<ygc1988@sina.com>、1006065333@qq.com<1006065333@qq.com>、1241346394<1241346394@qq.com>、1254007965@qq.com<1254007965@qq.com>、刘尧<liuyao66@ustc.edu.cn>、jbwang16<jbwang16@ustc.edu.cn>
主题	The change of co-authors about CDDIS-21-4379RR

Dear Authors：

Our recent manuscript CDDIS-21-4379RR, entitled "Cytochrome B5 Type A alleviates HCC metastasis via regulating STOML2 related autophagy and promoting sensitivity to Ruxolitinib." has been provisionally accepted for publication in Cell Death & Disease, lists you as a co-author. The author information has changed since the original submission.

In particular, I added Jizhou Wang as a co-author in view of his important contribution to the revision process. And I adjusted the order considering the contributions of all authors.

As the author information has changed, I ask for your opinion:

If you **agree** with all the final author information, please reply **I accept all the author information changes.**

If you **disagree** with the final author information or have other suggestions, please email me with details.

The changes of Author List and Contributions are as follows:

Final Author List:

Hongrui Guo[1]*, Shuhang Liang[2]*, Yan Wang[3]*, Shuo Zhou[2]*, Dalong Yin[2]*, Shugeng Zhang[2], Jizhou Wang[2], Dehai Wu[1], Kun Ma[1], Yufeng Liu[1], Linmao Sun[1], Changyong Ji[2], Xianying Li[2], Huanran Zhou[2], Guangchao Yang[1], Xinyu Guo[1], Tianming Cui[2], Zihao Li[1], Yao Liu[2]+, Jiabei Wang[2]+, Lianxin Liu[1,4]+

Final Author affiliations:

1.Department of Hepatic Surgery, Key Laboratory of Hepatosplenic Surgery, Ministry of Education, The First Affiliated Hospital of Harbin Medical University, Harbin, China

2.Department of Hepatobiliary Surgery, Anhui Province Key Laboratory of Hepatopancreatobiliary Surgery, The First Affiliated Hospital of USTC, Division of Life Sciences and Medicine, University of Science and Technology of China, Hefei, Anhui, 230001, China

3.Department of Colorectal Surgery, Harbin Medical University Cancer Hospital, Harbin, 150001 Heilongjiang, China

4. Lead contact

* These authors contributed equally to this work.

Final Author contributions:

Conceptualization, G.HR., L.SH., L.LX., W.JB., L.Y.; Methodology, Y.DL., W.DH., W.Y., Z.S., W.JZ., Z.SG., M.K., L.ZH.; Software, L.YF., S.LM., J.CY., L.XY., Y.GC.; Validation, G.HR., L.SH, Z.HR., L.ZH., G.XY., C.TM.; Formal Analysis, Z.S.,Y.DL.,L.LX., W.JB., L.Y.; Investigation, G.HR., L.SH, L.LX., W.JB.; Writing - Original Draft, G.HR., Z.SG.; Writing - Review & Editing, Z.HR., L.LX., W.JB., L.Y.; Visualization, G.HR., L.YF., S.LM., J.CY., L.XY., Y.GC.; Funding Acquisition, W.Y., L.Y., W.JB., L.LX.; Resources, L.LX., W.JB., L.Y.; Supervision, L.LX., W.JB., L.Y..

Thank you very much for your time and consideration. Wish you have a nice day!

Sincerely yours,

Lianxin Liu

主　题:	Re:The change of co-authors about CDDIS-21-4379RR
发件人:	"周烁" <zhouwill01@163.com> 2022-6-27 13:04:10
收件人:	liulx@ustc.edu.cn

Dear Professor Liu:

I accept all the author information changes.

Best wishes.

zhou shuo

At 2022-06-27 11:48:19, liulx@ustc.edu.cn wrote:

> **Dear Authors：**
>
> Our recent manuscript CDDIS-21-4379RR, entitled "Cytochrome B5 Type A alleviates HCC metastasis via regulating STOML2 related autophagy and promoting sensitivity to Ruxolitinib." has been provisionally accepted for publication in Cell Death & Disease, lists you as a co-author. The author information has changed since the original submission.
>
> In particular, I added Jizhou Wang as a co-author in view of his important contribution to the revision process. And I adjusted the order considering the contributions of all authors.
>
> **As the author information has changed, I ask for your opinion:**
>
> If you **agree** with all the final author information, please reply **I accept all the author information changes.**
>
> If you **disagree** with the final author information or have other suggestions, please email me with details.
>
> The changes of Author List and Contributions are as follows:

Final Author List:

Hongrui Guo[1*], Shuhang Liang[2*], Yan Wang[3*], Shuo Zhou[2*], Dalong Yin[2*], Shugeng Zhang[2], Jizhou Wang[2], Dehai Wu[1], Kun Ma[1], Yufeng Liu[1], Linmao Sun[1], Changyong Ji[2], Xianying Li[2], Huanran Zhou[2], Guangchao Yang[1], Xinyu Guo[1], Tianming Cui[2], Zihao Li[1], Yao Liu[2+], Jiabei Wang[2+], Lianxin Liu[1,4+]

Final Author affiliations:

1.Department of Hepatic Surgery, Key Laboratory of Hepatosplenic Surgery, Ministry of Education, The First Affiliated Hospital of Harbin Medical University, Harbin, China

2.Department of Hepatobiliary Surgery, Anhui Province Key Laboratory of Hepatopancreatobiliary Surgery, The First Affiliated Hospital of USTC, Division of Life Sciences and Medicine, University of Science and Technology of China, Hefei, Anhui, 230001, China

3.Department of Colorectal Surgery, Harbin Medical University Cancer Hospital, Harbin, 150001 Heilongjiang, China

4. Lead contact

* These authors contributed equally to this work.

Final Author contributions:

Conceptualization, G.HR., L.SH., L.LX., W.JB., L.Y.; Methodology, Y.DL., W.DH., W.Y., Z.S., W.JZ., Z.SG., M.K., L.ZH.; Software, L.YF., S.LM., J.CY., L.XY., Y.GC.; Validation, G.HR., L.SH, Z.HR., L.ZH., G.XY., C.TM.; Formal Analysis, Z.S.,Y.DL.,L.LX., W.JB., L.Y.; Investigation, G.HR., L.SH, L.LX., W.JB.; Writing - Original Draft, G.HR., Z.SG.; Writing - Review & Editing, Z.HR., L.LX., W.JB., L.Y.; Visualization, G.HR., L.YF., S.LM., J.CY., L.XY., Y.GC.; Funding Acquisition, W.Y., L.Y., W.JB., L.LX.; Resources, L.LX., W.JB., L.Y.; Supervision, L.LX., W.JB., L.Y..

Thank you very much for your time and consideration. Wish you have a nice day!

Sincerely yours,

Lianxin Liu

主　题:	Re:The change of co-authors about CDDIS-21-4379RR	
发件人:	DALONG <dalong48@163.com>	2022-6-27 14:04:03
收件人:	liulx@ustc.edu.cn	

Dear Prof. Liu,

I accept all the author information changes.
Best Regards

Dalong Yin

At 2022-06-27 11:48:19, liulx@ustc.edu.cn wrote:

> **Dear Authors:**
>
> Our recent manuscript CDDIS-21-4379RR, entitled "Cytochrome B5 Type A alleviates HCC metastasis via regulating STOML2 related autophagy and promoting sensitivity to Ruxolitinib." has been provisionally accepted for publication in Cell Death & Disease, lists you as a co-author. The author information has changed since the original submission.
>
> In particular, I added Jizhou Wang as a co-author in view of his important contribution to the revision process. And I adjusted the order considering the contributions of all authors.
>
> **As the author information has changed, I ask for your opinion:**
>
> If you **agree** with all the final author information, please reply **I accept all the author information changes.**
>
> If you **disagree** with the final author information or have other suggestions, please email me with details.
>
> The changes of Author List and Contributions are as follows:
>
> **Final Author List:**

Hongrui Guo[1*], Shuhang Liang[2*], Yan Wang[3*], Shuo Zhou[2*], Dalong Yin[2*], Shugeng Zhang[2], Jizhou Wang[2], Dehai Wu[1], Kun Ma[1], Yufeng Liu[1], Linmao Sun[1], Changyong Ji[2], Xianying Li[2], Huanran Zhou[2], Guangchao Yang[1], Xinyu Guo[1], Tianming Cui[2], Zihao Li[1], Yao Liu[2+], Jiabei Wang[2+], Lianxin Liu[1,4+]

Final Author affiliations:

1.Department of Hepatic Surgery, Key Laboratory of Hepatosplenic Surgery, Ministry of Education, The First Affiliated Hospital of Harbin Medical University, Harbin, China

2.Department of Hepatobiliary Surgery, Anhui Province Key Laboratory of Hepatopancreatobiliary Surgery, The First Affiliated Hospital of USTC, Division of Life Sciences and Medicine, University of Science and Technology of China, Hefei, Anhui, 230001, China

3.Department of Colorectal Surgery, Harbin Medical University Cancer Hospital, Harbin, 150001 Heilongjiang, China

4. Lead contact

* These authors contributed equally to this work.

Final Author contributions:

Conceptualization, G.HR., L.SH., L.LX., W.JB., L.Y.; Methodology, Y.DL., W.DH., W.Y., Z.S., W.JZ., Z.SG., M.K., L.ZH.; Software, L.YF., S.LM., J.CY., L.XY., Y.GC.; Validation, G.HR., L.SH Z.HR., L.ZH., G.XY., C.TM.; Formal Analysis, Z.S.,Y.DL.,L.LX., W.JB., L.Y.; Investigation, G.HR., L.SH, L.LX., W.JB.; Writing - Original Draft, G.HR., Z.SG.; Writing - Review & Editing, Z.HR., L.LX., W.JB., L.Y.; Visualization, G.HR., L.YF., S.LM., J.CY., L.XY., Y.GC.; Funding Acquisition, W.Y., L.Y., W.JB., L.LX.; Resources, L.LX., W.JB., L.Y.; Supervision, L.LX., W.JB., L.Y..

Thank you very much for your time and consideration. Wish you have a nice day!

Sincerely yours，

Lianxin Liu

主　题:	Re:The change of co-authors about CDDIS-21-4379RR
发件人:	"张树庚" <shugeng517@163.com> 2022-6-27 14:43:45
收件人:	liulx@ustc.edu.cn

Dear Professor Liu:

I accept all the author information changes.

Best wishes.
Zhang Shugeng

At 2022-06-27 11:48:19, liulx@ustc.edu.cn wrote:

> **Dear Authors：**
>
> Our recent manuscript CDDIS-21-4379RR, entitled "Cytochrome B5 Type A alleviates HCC metastasis via regulating STOML2 related autophagy and promoting sensitivity to Ruxolitinib." has been provisionally accepted for publication in Cell Death & Disease, lists you as a co-author. The author information has changed since the original submission.
>
> In particular, I added Jizhou Wang as a co-author in view of his important contribution to the revision process. And I adjusted the order considering the contributions of all authors.
>
> **As the author information has changed, I ask for your opinion:**
>
> If you **agree** with all the final author information, please reply **I accept all the author information changes.**
>
> If you **disagree** with the final author information or have other suggestions, please email me with details.
>
> The changes of Author List and Contributions are as follows:

Final Author List:

Hongrui Guo[1*], Shuhang Liang[2*], Yan Wang[3*], Shuo Zhou[2*], Dalong Yin[2*], Shugeng Zhang[2], Jizhou Wang[2], Dehai Wu[1], Kun Ma[1], Yufeng Liu[1], Linmao Sun[1], Changyong Ji[2], Xianying Li[2], Huanran Zhou[2], Guangchao Yang[1], Xinyu Guo[1], Tianming Cui[2], Zihao Li[1], Yao Liu[2+], Jiabei Wang[2+], Lianxin Liu[1,4+]

Final Author affiliations:

1.Department of Hepatic Surgery, Key Laboratory of Hepatosplenic Surgery, Ministry of Education, The First Affiliated Hospital of Harbin Medical University, Harbin, China

2.Department of Hepatobiliary Surgery, Anhui Province Key Laboratory of Hepatopancreatobiliary Surgery, The First Affiliated Hospital of USTC, Division of Life Sciences and Medicine, University of Science and Technology of China, Hefei, Anhui, 230001, China

3.Department of Colorectal Surgery, Harbin Medical University Cancer Hospital, Harbin, 150001 Heilongjiang, China

4. Lead contact

* These authors contributed equally to this work.

Final Author contributions:

Conceptualization, G.HR., L.SH., L.LX., W.JB., L.Y.; Methodology, Y.DL., W.DH., W.Y., Z.S., W.JZ., Z.SG., M.K., L.ZH.; Software, L.YF., S.LM., J.CY., L.XY., Y.GC.; Validation, G.HR., L.SH Z.HR., L.ZH., G.XY., C.TM.; Formal Analysis, Z.S.,Y.DL.,L.LX., W.JB., L.Y.; Investigation, G.HR., L.SH, L.LX., W.JB.; Writing - Original Draft, G.HR., Z.SG.; Writing - Review & Editing, Z.HR., L.LX., W.JB., L.Y.; Visualization, G.HR., L.YF., S.LM., J.CY., L.XY., Y.GC.; Funding Acquisition, W.Y., L.Y., W.JB., L.LX.; Resources, L.LX., W.JB., L.Y.; Supervision, L.LX., W.JB., L.Y..

Thank you very much for your time and consideration. Wish you have a nice day!

Sincerely yours，

Lianxin Liu

主　题:	Re: The change of co-authors about CDDIS-21-4379RR	
发件人:	"wangjoe@ustc.edu.cn" <wangjoe@ustc.edu.cn>	2022-6-28 7:28:59
收件人:	liulx <liulx@ustc.edu.cn>	

Dear Professor Lianxin Liu,
I accept all the author information changes.

Jizhou Wang

wangjoe@ustc.edu.cn

邮箱：wangjoe@ustc.edu.cn

---- 回复的原邮件 ----

发件人	liulx<liulx@ustc.edu.cn>
日期	2022年06月27日 14:23
收件人	wangjoe<wangjoe@ustc.edu.cn>
主题	The change of co-authors about CDDIS-21-4379RR

Dear Authors：Our recent manuscript CDDIS-21-4379RR, entitled "Cytochrome B5 Type A alleviates HCC metastasis via regulating STOML2 related autophagy and promoting sensitivity to Ruxolitinib." has been provisionally accepted for publication in Cell Death & Disease, lists you as a co-author. The author information has changed since the original submission. In particular, I added Jizhou Wang as a co-author in view of his important contribution to the revision process. And I adjusted the order considering the contributions of all authors. As the author information has changed, I ask for your opinion:If you agree with all the final author information, please reply I accept all the author information changes.If you disagree with the final author information or have other suggestions, please email me with details. The changes of Author List and Contributions are as follows: Final Author List:Hongrui Guo1*, Shuhang Liang2*, Yan Wang3*, Shuo Zhou2*, Dalong Yin2*, Shugeng Zhang2, Jizhou Wang2, Dehai Wu1, Kun Ma1, Yufeng Liu1, Linmao Sun1, Changyong Ji2, Xianying Li2, Huanran Zhou2, Guangchao Yang1, Xinyu Guo1, Tianming Cui2, Zihao Li1, Yao Liu2+, Jiabei Wang2+, Lianxin Liu1,4+ Final Author affiliations:1.Department of Hepatic Surgery, Key Laboratory of Hepatosplenic Surgery, Ministry of Education, The First Affiliated Hospital of Harbin Medical University, Harbin, China2.Department of Hepatobiliary Surgery, Anhui Province Key Laboratory of Hepatopancreatobiliary Surgery, The First Affiliated Hospital of USTC, Division of Life Sciences and Medicine, University of Science and Technology of China, Hefei, Anhui, 230001, China3.Department of Colorectal Surgery, Harbin Medical University Cancer Hospital, Harbin, 150001 Heilongjiang, China4. Lead contact* These authors contributed equally to this work. Final Author contributions: Conceptualization, G.HR., L.SH., L.LX., W.JB., L.Y.; Methodology, Y.DL., W.DH., W.Y., Z.S., W.JZ., Z.SG., M.K., L.ZH.; Software, L.YF., S.LM., J.CY., L.XY., Y.GC.; Validation, G.HR., L.SH, Z.HR., L.ZH., G.XY., C.TM.; Formal Analysis, Z.S.,Y.DL.,L.LX., W.JB., L.Y.; Investigation, G.HR., L.SH, L.LX., W.JB.; Writing - Original Draft, G.HR., Z.SG.; Writing - Review & Editing, Z.HR., L.LX., W.JB., L.Y.; Visualization, G.HR., L.YF., S.LM., J.CY., L.XY., Y.GC.; Funding Acquisition, W.Y., L.Y., W.JB., L.LX.; Resources, L.LX., W.JB., L.Y.; Supervision, L.LX., W.JB., L.Y.. Thank you very much for your time and consideration. Wish you have a nice day!Sincerely yours，Lianxin Liu

主　题:	回复：The change of co-authors about CDDIS-21-4379RR
发件人:	"Dr. Wu" <tianxiadiyi_12345@126.com> 2022-6-27 16:11:49
收件人:	"liulx@ustc.edu.cn" <liulx@ustc.edu.cn>, "郭泓瑞" <hongrui7456@163.com>, "梁书航" <154652031@qq.com>, "wangyan2016md@126.com" <wangyan2016md@126.com>, "zhouwill01@163.com" <zhouwill01@163.com>, "尹大龙" <dalong48@163.com>, "张树庚" <shugeng517@163.com>, "王继洲" <wangjizhou1984@hotmail.com>, "1694769800@qq.com" <1694769800@qq.com>, 413951217 <413951217@qq.com>, "lemonzhutou@163.com" <lemonzhutou@163.com>, "cyji@mail.ustc.edu.cn" <cyji@mail.ustc.edu.cn>, "lidonglps@qq.com" <lidonglps@qq.com>, "drzhr1026@hotmail.com" <drzhr1026@hotmail.com>, "ygc1988@sina.com" <ygc1988@sina.com>, "1006065333@qq.com" <1006065333@qq.com>, 1241346394 <1241346394@qq.com>, "1254007965@qq.com" <1254007965@qq.com>, "刘尧" <liuyao66@ustc.edu.cn>, jbwang16 <jbwang16@ustc.edu.cn>

Dear Professor Lianxin Liu,
I accept all the author information changes.

Dehai Wu

发自我的手机

-------- 原始邮件 --------
发件人： liulx@ustc.edu.cn
日期： 2022年6月27日周一 中午11:48
收件人： 郭泓瑞 <hongrui7456@163.com>, 梁书航 <154652031@qq.com>, wangyan2016md@126.com, zhouwill01@163.com, 尹大龙 <dalong48@163.com>, 张树庚 <shugeng517@163.com>, 王继洲 <wangjizhou1984@hotmail.com>, tianxiadiyi_12345@126.com, 1694769800@qq.com, 413951217 <413951217@qq.com>, lemonzhutou@163.com, cyji@mail.ustc.edu.cn, lidonglps@qq.com, drzhr1026@hotmail.com, ygc1988@sina.com, 1006065333@qq.com, 1241346394 <1241346394@qq.com>, 1254007965@qq.com, 刘尧 <liuyao66@ustc.edu.cn>, jbwang16 <jbwang16@ustc.edu.cn>
主 题： The change of co-authors about CDDIS-21-4379RR

Dear Authors：

Our recent manuscript CDDIS-21-4379RR, entitled "Cytochrome B5 Type A alleviates HCC metastasis via regulating STOML2 related autophagy and promoting sensitivity to Ruxolitinib." has been provisionally accepted for publication in Cell Death & Disease, lists you as a co-author. The author information has changed since the original submission.

In particular, I added Jizhou Wang as a co-author in view of his important contribution to the revision process. And I adjusted the order considering the contributions of all authors.

As the author information has changed, I ask for your opinion:

If you **agree** with all the final author information, please reply **I accept all the author information changes.**

If you **disagree** with the final author information or have other suggestions, please email me with details.

The changes of Author List and Contributions are as follows:

Final Author List:

Hongrui Guo[1*], Shuhang Liang[2*], Yan Wang[3*], Shuo Zhou[2*], Dalong Yin[2*], Shugeng Zhang[2], Jizhou Wang[2], Dehai Wu[1], Kun Ma[1], Yufeng Liu[1], Linmao Sun[1], Changyong Ji[2], Xianying Li[2], Huanran Zhou[2], Guangchao Yang[1], Xinyu Guo[1], Tianming Cui[2], Zihao Li[1], Yao Liu[2+], Jiabei Wang[2+], Lianxin Liu[1,4+]

Final Author affiliations:

1.Department of Hepatic Surgery, Key Laboratory of Hepatosplenic Surgery, Ministry of Education, The First Affiliated Hospital of Harbin Medical University, Harbin, China

2.Department of Hepatobiliary Surgery, Anhui Province Key Laboratory of Hepatopancreatobiliary Surgery, The First Affiliated Hospital of USTC, Division of Life Sciences and Medicine, University of Science and Technology of China, Hefei, Anhui, 230001, China

3.Department of Colorectal Surgery, Harbin Medical University Cancer Hospital, Harbin, 150001 Heilongjiang, China

4. Lead contact

* These authors contributed equally to this work.

Final Author contributions:

Conceptualization, G.HR., L.SH., L.LX., W.JB., L.Y.; Methodology, Y.DL., W.DH., W.Y., Z.S., W.JZ., Z.SG., M.K., L.ZH.; Software, L.YF., S.LM., J.CY., L.XY., Y.GC.; Validation, G.HR., L.SH, Z.HR., L.ZH., G.XY., C.TM.; Formal Analysis, Z.S.,Y.DL.,L.LX., W.JB., L.Y.; Investigation, G.HR., L.SH, L.LX., W.JB.; Writing - Original Draft, G.HR., Z.SG.; Writing - Review & Editing, Z.HR., L.LX., W.JB., L.Y.; Visualization, G.HR., L.YF., S.LM., J.CY., L.XY., Y.GC.; Funding Acquisition, W.Y., L.Y., W.JB., L.LX.; Resources, L.LX., W.JB., L.Y.; Supervision, L.LX., W.JB., L.Y..

Thank you very much for your time and consideration. Wish you have a nice day!

Sincerely yours,

Lianxin Liu

主　题:	Re: The change of co-authors about CDDIS-21-4379RR
发件人:	MK <1694769800@qq.com> 2022-6-27 14:29:17
收件人:	liulx <liulx@ustc.edu.cn>

Dear Professor Lianxin Liu,
I accept all the author information changes.
Kun Ma

---Original---

From: "liulx"<liulx@ustc.edu.cn>
Date: Mon, Jun 27, 2022 11:48 AM
To: "郭泓瑞"<hongrui7456@163.com>;"梁书航"<154652031@qq.com>;"wangyan2016md" <wangyan2016md@126.com>;"zhouwill01"<zhouwill01@163.com>;"尹大龙"<dalong48@163.com>;"张树庚" <shugeng517@163.com>;"王继洲"<wangjizhou1984@hotmail.com>;"tianxiadiyi_12345" <tianxiadiyi_12345@126.com>;"1694769800"<1694769800@qq.com>;"413951217"<413951217@qq.com>;"lemonzhutou" <lemonzhutou@163.com>;"cyji"<cyji@mail.ustc.edu.cn>;"lidonglps"<lidonglps@qq.com>;"drzhr1026" <drzhr1026@hotmail.com>;"ygc1988"<ygc1988@sina.com>;"1006065333"<1006065333@qq.com>;"1241346394" <1241346394@qq.com>;"1254007965"<1254007965@qq.com>;"刘尧"<liuyao66@ustc.edu.cn>;"jbwang16" <jbwang16@ustc.edu.cn>;
Subject: The change of co-authors about CDDIS-21-4379RR

Dear Authors:

Our recent manuscript CDDIS-21-4379RR, entitled "Cytochrome B5 Type A alleviates HCC metastasis via regulating STOML2 related autophagy and promoting sensitivity to Ruxolitinib." has been provisionally accepted for publication in Cell Death & Disease, lists you as a co-author. The author information has changed since the original submission.

In particular, I added Jizhou Wang as a co-author in view of his important contribution to the revision process. And I adjusted the order considering the contributions of all authors.

As the author information has changed, I ask for your opinion:

If you **agree** with all the final author information, please reply **I accept all the author information changes.**

If you **disagree** with the final author information or have other suggestions, please email me with details.

The changes of Author List and Contributions are as follows:

Final Author List:

Hongrui Guo[1]*, Shuhang Liang[2]*, Yan Wang[3]*, Shuo Zhou[2]*, Dalong Yin[2]*, Shugeng Zhang[2], Jizhou Wang[2], Dehai Wu[1], Kun Ma[1], Yufeng Liu[1], Linmao Sun[1], Changyong Ji[2], Xianying Li[2], Huanran Zhou[2], Guangchao Yang[1], Xinyu Guo[1], Tianming Cui[2], Zihao Li[1], Yao Liu[2]+, Jiabei Wang[2]+, Lianxin Liu[1,4]+

Final Author affiliations:

1.Department of Hepatic Surgery, Key Laboratory of Hepatosplenic Surgery, Ministry of Education, The First Affiliated Hospital of Harbin Medical University, Harbin, China

2.Department of Hepatobiliary Surgery, Anhui Province Key Laboratory of Hepatopancreatobiliary Surgery, The First Affiliated Hospital of USTC, Division of Life Sciences and Medicine, University of Science and Technology of China, Hefei, Anhui, 230001, China

3.Department of Colorectal Surgery, Harbin Medical University Cancer Hospital, Harbin, 150001 Heilongjiang, China

4. Lead contact

* These authors contributed equally to this work.

Final Author contributions:

Conceptualization, G.HR., L.SH., L.LX., W.JB., L.Y.; Methodology, Y.DL., W.DH., W.Y., Z.S., W.JZ., Z.SG., M.K., L.ZH.; Software, L.YF., S.LM., J.CY., L.XY., Y.GC.; Validation, G.HR., L.SH, Z.HR., L.ZH., G.XY., C.TM.; Formal Analysis, Z.S.,Y.DL.,L.LX., W.JB., L.Y.; Investigation, G.HR., L.SH, L.LX., W.JB.; Writing - Original Draft, G.HR., Z.SG.; Writing - Review & Editing, Z.HR., L.LX., W.JB., L.Y.; Visualization, G.HR., L.YF., S.LM., J.CY., L.XY., Y.GC.; Funding Acquisition, W.Y., L.Y., W.JB., L.LX.; Resources, L.LX., W.JB., L.Y.; Supervision, L.LX., W.JB., L.Y..

Thank you very much for your time and consideration. Wish you have a nice day!

Sincerely yours,

Lianxin Liu

主　题:	回复：The change of co-authors about CDDIS-21-4379RR	
发件人:	"刘雨枫" <413951217@qq.com>	2022-6-27 12:47:27
收件人:	liulx <liulx@ustc.edu.cn>	

Dear Lianxin Liu，

I accept all the author information changes.

Yufeng Liu

------------------ 原始邮件 ------------------

发件人: "liulx" <liulx@ustc.edu.cn>;
发送时间: 2022年6月27日(星期一) 中午11:48
收件人: "郭泓瑞"<hongrui7456@163.com>;"梁书航"<154652031@qq.com>;"wangyan2016md"<wangyan2016md@126.com>;"zhouwill01"<zhouwill01@163.com>;"尹大龙"<dalong48@163.com>;"张树庚"<shugeng517@163.com>;"王继洲"<wangjizhou1984@hotmail.com>;"tianxiadiyi_12345"<tianxiadiyi_12345@126.com>;"1694769800"<1694769800@qq.com>;"刘雨枫"<413951217@qq.com>;"lemonzhutou"<lemonzhutou@163.com>;"cyji"<cyji@mail.ustc.edu.cn>;"lidonglps"<lidonglps@qq.com>;"drzhr1026"<drzhr1026@hotmail.com>;"ygc1988"<ygc1988@sina.com>;"1006065333"<1006065333@qq.com>;"1241346394"<1241346394@qq.com>;"1254007965"<1254007965@qq.com>;"刘尧"<liuyao66@ustc.edu.cn>;"jbwang16"<jbwang16@ustc.edu.cn>;
主题: The change of co-authors about CDDIS-21-4379RR

Dear Authors：

Our recent manuscript CDDIS-21-4379RR, entitled "Cytochrome B5 Type A alleviates HCC metastasis via regulating STOML2 related autophagy and promoting sensitivity to Ruxolitinib." has been provisionally accepted for publication in Cell Death & Disease, lists you as a co-author. The author information has changed since the original submission.

In particular, I added Jizhou Wang as a co-author in view of his important contribution to the revision process. And I adjusted the order considering the contributions of all authors.

As the author information has changed, I ask for your opinion:

If you **agree** with all the final author information, please reply **I accept all the author information changes.**

If you **disagree** with the final author information or have other suggestions, please email me with details.

The changes of Author List and Contributions are as follows:

Final Author List:

Hongrui Guo[1*], Shuhang Liang[2*], Yan Wang[3*], Shuo Zhou[2*], Dalong Yin[2*], Shugeng Zhang[2], Jizhou Wang[2], Dehai Wu[1], Kun Ma[1], Yufeng Liu[1], Linmao Sun[1], Changyong Ji[2], Xianying Li[2], Huanran Zhou[2], Guangchao Yang[1], Xinyu Guo[1], Tianming Cui[2], Zihao Li[1], Yao Liu[2+], Jiabei Wang[2+], Lianxin Liu[1,4+]

Final Author affiliations:

1.Department of Hepatic Surgery, Key Laboratory of Hepatosplenic Surgery, Ministry of Education, The First Affiliated Hospital of Harbin Medical University, Harbin, China

2.Department of Hepatobiliary Surgery, Anhui Province Key Laboratory of Hepatopancreatobiliary Surgery, The First Affiliated Hospital of USTC, Division of Life Sciences and Medicine, University of Science and Technology of China, Hefei, Anhui, 230001, China

3.Department of Colorectal Surgery, Harbin Medical University Cancer Hospital, Harbin, 150001 Heilongjiang, China

4. Lead contact

* These authors contributed equally to this work.

Final Author contributions:

Conceptualization, G.HR., L.SH., L.LX., W.JB., L.Y.; Methodology, Y.DL., W.DH., W.Y., Z.S., W.JZ., Z.SG., M.K., L.ZH.; Software, L.YF., S.LM., J.CY., L.XY., Y.GC.; Validation, G.HR., L.SH, Z.HR., L.ZH., G.XY., C.TM.; Formal Analysis, Z.S.,Y.DL.,L.LX., W.JB., L.Y.; Investigation, G.HR., L.SH, L.LX., W.JB.; Writing - Original Draft, G.HR., Z.SG.; Writing - Review & Editing, Z.HR., L.LX., W.JB., L.Y.; Visualization, G.HR., L.YF., S.LM., J.CY., L.XY., Y.GC.; Funding Acquisition, W.Y., L.Y., W.JB., L.LX.; Resources, L.LX., W.JB., L.Y.; Supervision, L.LX., W.JB., L.Y..

Thank you very much for your time and consideration. Wish you have a nice day!

Sincerely yours,

Lianxin Liu

主　题:	Re:The change of co-authors about CDDIS-21-4379RR
发件人:	"孙林茂" <lemonzhutou@163.com> 2022-6-27 16:07:39
收件人:	liulx@ustc.edu.cn

Dear Lianxin Liu,

I accept all the author information changes.

Linmao Sun
2022.06.27

At 2022-06-27 11:48:19, liulx@ustc.edu.cn wrote:

> **Dear Authors:**
>
> Our recent manuscript CDDIS-21-4379RR, entitled "Cytochrome B5 Type A alleviates HCC metastasis via regulating STOML2 related autophagy and promoting sensitivity to Ruxolitinib." has been provisionally accepted for publication in Cell Death & Disease, lists you as a co-author. The author information has changed since the original submission.
>
> In particular, I added Jizhou Wang as a co-author in view of his important contribution to the revision process. And I adjusted the order considering the contributions of all authors.
>
> **As the author information has changed, I ask for your opinion:**
>
> If you **agree** with all the final author information, please reply **I accept all the author information changes.**
>
> If you **disagree** with the final author information or have other suggestions, please email me with details.
>
> The changes of Author List and Contributions are as follows:
>
> **Final Author List:**

Hongrui Guo[1*], Shuhang Liang[2*], Yan Wang[3*], Shuo Zhou[2*], Dalong Yin[2*], Shugeng Zhang[2], Jizhou Wang[2], Dehai Wu[1], Kun Ma[1], Yufeng Liu[1], Linmao Sun[1], Changyong Ji[2], Xianying Li[2], Huanran Zhou[2], Guangchao Yang[1], Xinyu Guo[1], Tianming Cui[2], Zihao Li[1], Yao Liu[2+], Jiabei Wang[2+], Lianxin Liu[1,4+]

Final Author affiliations:

1.Department of Hepatic Surgery, Key Laboratory of Hepatosplenic Surgery, Ministry of Education, The First Affiliated Hospital of Harbin Medical University, Harbin, China

2.Department of Hepatobiliary Surgery, Anhui Province Key Laboratory of Hepatopancreatobiliary Surgery, The First Affiliated Hospital of USTC, Division of Life Sciences and Medicine, University of Science and Technology of China, Hefei, Anhui, 230001, China

3.Department of Colorectal Surgery, Harbin Medical University Cancer Hospital, Harbin, 150001 Heilongjiang, China

4. Lead contact

* These authors contributed equally to this work.

Final Author contributions:

Conceptualization, G.HR., L.SH., L.LX., W.JB., L.Y.; Methodology, Y.DL., W.DH., W.Y., Z.S., W.JZ., Z.SG., M.K., L.ZH.; Software, L.YF., S.LM., J.CY., L.XY., Y.GC.; Validation, G.HR., L.SH Z.HR., L.ZH., G.XY., C.TM.; Formal Analysis, Z.S.,Y.DL.,L.LX., W.JB., L.Y.; Investigation, G.HR., L.SH, L.LX., W.JB.; Writing - Original Draft, G.HR., Z.SG.; Writing - Review & Editing, Z.HR., L.LX., W.JB., L.Y.; Visualization, G.HR., L.YF., S.LM., J.CY., L.XY., Y.GC.; Funding Acquisition, W.Y., L.Y., W.JB., L.LX.; Resources, L.LX., W.JB., L.Y.; Supervision, L.LX., W.JB., L.Y..

Thank you very much for your time and consideration. Wish you have a nice day!

Sincerely yours，

Lianxin Liu

主　题:	Re: The change of co-authors about CDDIS-21-4379RR - All accepted	
发件人:	cyji@mail.ustc.edu.cn	2022-6-27 13:31:08
收件人:	liulx@ustc.edu.cn	

Dear Professor Lianxin Liu,
I accept all the author information changes.
Ji Changyong
2022.06.27

-----原始邮件-----
发件人:liulx@ustc.edu.cn
发送时间:2022-06-27 11:48:19 (星期一)
收件人: "郭泓瑞" <hongrui7456@163.com>, "梁书航" <154652031@qq.com>, wangyan2016md@126.com, zhouwill01@163.com, "尹大龙" <dalong48@163.com>, "张树庚" <shugeng517@163.com>, "王继洲" <wangjizhou1984@hotmail.com>, tianxiadiyi_12345@126.com, 1694769800@qq.com, 413951217 <413951217@qq.com>, lemonzhutou@163.com, cyji@mail.ustc.edu.cn, lidonglps@qq.com, drzhr1026@hotmail.com, ygc1988@sina.com, 1006065333@qq.com, 1241346394 <1241346394@qq.com>, 1254007965@qq.com, "刘尧" <liuyao66@ustc.edu.cn>, jbwang16 <jbwang16@ustc.edu.cn>
抄送:
主题: The change of co-authors about CDDIS-21-4379RR

Dear Authors:

Our recent manuscript CDDIS-21-4379RR, entitled "Cytochrome B5 Type A alleviates HCC metastasis via regulating STOML2 related autophagy and promoting sensitivity to Ruxolitinib." has been provisionally accepted for publication in Cell Death & Disease, lists you as a co-author. The author information has changed since the original submission.

In particular, I added Jizhou Wang as a co-author in view of his important contribution to the revision process. And I adjusted the order considering the contributions of all authors.

As the author information has changed, I ask for your opinion:

If you **agree** with all the final author information, please reply **I accept all the author information changes.**

If you **disagree** with the final author information or have other suggestions, please email me with details.

The changes of Author List and Contributions are as follows:

Final Author List:

Hongrui Guo[1]*, Shuhang Liang[2]*, Yan Wang[3]*, Shuo Zhou[2]*, Dalong Yin[2]*, Shugeng Zhang[2], Jizhou Wang[2], Dehai Wu[1], Kun Ma[1], Yufeng Liu[1], Linmao Sun[1], Changyong Ji[2], Xianying Li[2], Huanran Zhou[2], Guangchao Yang[1], Xinyu Guo[1], Tianming Cui[2], Zihao Li[1], Yao Liu[2]+, Jiabei Wang[2]+, Lianxin Liu[1,4]+

Final Author affiliations:

1.Department of Hepatic Surgery, Key Laboratory of Hepatosplenic Surgery, Ministry of Education, The First Affiliated Hospital of Harbin Medical University, Harbin, China

2.Department of Hepatobiliary Surgery, Anhui Province Key Laboratory of Hepatopancreatobiliary Surgery, The First Affiliated Hospital of USTC, Division of Life Sciences and Medicine, University of Science and Technology of China, Hefei, Anhui, 230001, China

3.Department of Colorectal Surgery, Harbin Medical University Cancer Hospital, Harbin, 150001 Heilongjiang, China

4. Lead contact

* These authors contributed equally to this work.

Final Author contributions:

Conceptualization, G.HR., L.SH., L.LX., W.JB., L.Y.; Methodology, Y.DL., W.DH., W.Y., Z.S., W.JZ., Z.SG., M.K., L.ZH.; Software, L.YF., S.LM., J.CY., L.XY., Y.GC.; Validation, G.HR., L.SH, Z.HR., L.ZH., G.XY., C.TM.; Formal Analysis, Z.S.,Y.DL.,L.LX., W.JB., L.Y.; Investigation, G.HR., L.SH, L.LX., W.JB.; Writing - Original Draft, G.HR., Z.SG.; Writing - Review & Editing, Z.HR., L.LX., W.JB., L.Y.; Visualization, G.HR., L.YF., S.LM., J.CY., L.XY., Y.GC.; Funding Acquisition, W.Y., L.Y., W.JB., L.LX.; Resources, L.LX., W.JB., L.Y.; Supervision, L.LX., W.JB., L.Y..

Thank you very much for your time and consideration. Wish you have a nice day!

Sincerely yours,

Lianxin Liu

主　题:	回复: The change of co-authors about CDDIS-21-4379RR	
发件人:	"Ace、" <lidonglps@qq.com>	2022-6-27 12:49:59
收件人:	liulx <liulx@ustc.edu.cn>	

Dear Professor Liu
I accept all the author information changes.
Sincerely yours,
Xianying Li

------------------ 原始邮件 ------------------

发件人: "liulx" <liulx@ustc.edu.cn>;
发送时间: 2022年6月27日(星期一) 中午11:48
收件人: "郭泓瑞"<hongrui7456@163.com>;"梁书航"<154652031@qq.com>;"wangyan2016md" <wangyan2016md@126.com>;"zhouwill01"<zhouwill01@163.com>;"尹大龙"<dalong48@163.com>;"张树庚" <shugeng517@163.com>;"王继洲"<wangjizhou1984@hotmail.com>;"tianxiadiyi_12345" <tianxiadiyi_12345@126.com>;"1694769800"<1694769800@qq.com>;"413951217"<413951217@qq.com>;"lemonzhutou" <lemonzhutou@163.com>;"cyji"<cyji@mail.ustc.edu.cn>;"Ace、"<lidonglps@qq.com>;"drzhr1026" <drzhr1026@hotmail.com>;"ygc1988"<ygc1988@sina.com>;"1006065333"<1006065333@qq.com>;"1241346394" <1241346394@qq.com>;"1254007965"<1254007965@qq.com>;"刘尧"<liuyao66@ustc.edu.cn>;"jbwang16" <jbwang16@ustc.edu.cn>;
主题: The change of co-authors about CDDIS-21-4379RR

Dear Authors:

Our recent manuscript CDDIS-21-4379RR, entitled "Cytochrome B5 Type A alleviates HCC metastasis via regulating STOML2 related autophagy and promoting sensitivity to Ruxolitinib." has been provisionally accepted for publication in Cell Death & Disease, lists you as a co-author. The author information has changed since the original submission.

In particular, I added Jizhou Wang as a co-author in view of his important contribution to the revision process. And I adjusted the order considering the contributions of all authors.

As the author information has changed, I ask for your opinion:

If you **agree** with all the final author information, please reply **I accept all the author information changes.**

If you **disagree** with the final author information or have other suggestions, please email me with details.

The changes of Author List and Contributions are as follows:

Final Author List:

Hongrui Guo[1]*, Shuhang Liang[2]*, Yan Wang[3]*, Shuo Zhou[2]*, Dalong Yin[2]*, Shugeng Zhang[2], Jizhou Wang[2], Dehai Wu[1], Kun Ma[1], Yufeng Liu[1], Linmao Sun[1], Changyong Ji[2], Xianying Li[2], Huanran Zhou[2], Guangchao Yang[1], Xinyu Guo[1], Tianming Cui[2], Zihao Li[1], Yao Liu[2]+, Jiabei Wang[2]+, Lianxin Liu[1,4]+

Final Author affiliations:

1.Department of Hepatic Surgery, Key Laboratory of Hepatosplenic Surgery, Ministry of Education, The First Affiliated Hospital of Harbin Medical University, Harbin, China

2.Department of Hepatobiliary Surgery, Anhui Province Key Laboratory of Hepatopancreatobiliary Surgery, The First Affiliated Hospital of USTC, Division of Life Sciences and Medicine, University of Science and Technology of China, Hefei, Anhui, 230001, China

3.Department of Colorectal Surgery, Harbin Medical University Cancer Hospital, Harbin, 150001 Heilongjiang, China

4. Lead contact

* These authors contributed equally to this work.

Final Author contributions:

Conceptualization, G.HR., L.SH., L.LX., W.JB., L.Y.; Methodology, Y.DL., W.DH., W.Y., Z.S., W.JZ., Z.SG., M.K., L.ZH.; Software, L.YF., S.LM., J.CY., L.XY., Y.GC.; Validation, G.HR., L.SH, Z.HR., L.ZH., G.XY., C.TM.; Formal Analysis, Z.S.,Y.DL.,L.LX., W.JB., L.Y.; Investigation, G.HR., L.SH, L.LX., W.JB.; Writing - Original Draft, G.HR., Z.SG.; Writing - Review & Editing, Z.HR., L.LX., W.JB., L.Y.; Visualization, G.HR., L.YF., S.LM., J.CY., L.XY., Y.GC.; Funding Acquisition, W.Y., L.Y., W.JB., L.LX.; Resources, L.LX., W.JB., L.Y.; Supervision, L.LX., W.JB., L.Y..

Thank you very much for your time and consideration. Wish you have a nice day!

Sincerely yours,

Lianxin Liu

主　题:	回复: The change of co-authors about CDDIS-21-4379RR	
发件人:	"周 焕然" <drzhr1026@hotmail.com>	2022-6-27 15:22:17
收件人:	"liulx@ustc.edu.cn" <liulx@ustc.edu.cn>	

Dear Professor Lianxin Liu,

I accept all the author information changes.

Best wishes,

Huanran Zhou

发件人: liulx@ustc.edu.cn <liulx@ustc.edu.cn>
发送时间: 2022年6月27日 11:48
收件人: 郭泓瑞 <hongrui7456@163.com>; 梁书航 <154652031@qq.com>; wangyan2016md@126.com <wangyan2016md@126.com>; zhouwill01@163.com <zhouwill01@163.com>; 尹大龙 <dalong48@163.com>; 张树庚 <shugeng517@163.com>; 王继洲 <wangjizhou1984@hotmail.com>; tianxiadiyi_12345@126.com <tianxiadiyi_12345@126.com>; 1694769800@qq.com <1694769800@qq.com>; 413951217 <413951217@qq.com>; lemonzhutou@163.com <lemonzhutou@163.com>; cyji@mail.ustc.edu.cn <cyji@mail.ustc.edu.cn>; lidonglps@qq.com <lidonglps@qq.com>; drzhr1026@hotmail.com <drzhr1026@hotmail.com>; ygc1988@sina.com <ygc1988@sina.com>; 1006065333@qq.com <1006065333@qq.com>; 1241346394 <1241346394@qq.com>; 1254007965@qq.com <1254007965@qq.com>; 刘尧 <liuyao66@ustc.edu.cn>; jbwang16 <jbwang16@ustc.edu.cn>
主题: The change of co-authors about CDDIS-21-4379RR

Dear Authors：
Our recent manuscript CDDIS-21-4379RR, entitled "Cytochrome B5 Type A alleviates HCC metastasis via regulating STOML2 related autophagy and promoting sensitivity to Ruxolitinib." has been provisionally accepted for publication in Cell Death & Disease, lists you as a co-author. The author information has changed since the original submission.

In particular, I added Jizhou Wang as a co-author in view of his important contribution to the revision process. And I adjusted the order considering the contributions of all authors.

As the author information has changed, I ask for your opinion:
If you **agree** with all the final author information, please reply **I accept all the author information changes.**
If you **disagree** with the final author information or have other suggestions, please email me with details.

The changes of Author List and Contributions are as follows:

Final Author List:
Hongrui Guo[1*], Shuhang Liang[2*], Yan Wang[3*], Shuo Zhou[2*], Dalong Yin[2*], Shugeng Zhang[2], Jizhou Wang[2], Dehai Wu[1], Kun Ma[1], Yufeng Liu[1], Linmao Sun[1], Changyong Ji[2], Xianying Li[2], Huanran Zhou[2], Guangchao Yang[1], Xinyu Guo[1], Tianming Cui[2], Zihao Li[1], Yao Liu[2+], Jiabei Wang[2+], Lianxin Liu[1,4+]

Final Author affiliations:
1.Department of Hepatic Surgery, Key Laboratory of Hepatosplenic Surgery, Ministry of Education, The First Affiliated Hospital of Harbin Medical University, Harbin, China
2.Department of Hepatobiliary Surgery, Anhui Province Key Laboratory of Hepatopancreatobiliary Surgery, The First Affiliated Hospital of USTC, Division of Life Sciences and Medicine, University of Science and Technology of China, Hefei, Anhui, 230001, China
3.Department of Colorectal Surgery, Harbin Medical University Cancer Hospital, Harbin, 150001 Heilongjiang, China

4. Lead contact
* These authors contributed equally to this work.

Final Author contributions:
Conceptualization, G.HR., L.SH., L.LX., W.JB., L.Y.; Methodology, Y.DL., W.DH., W.Y., Z.S., W.JZ., Z.SG., M.K., L.ZH.; Software, L.YF., S.LM., J.CY., L.XY., Y.GC.; Validation, G.HR., L.SH, Z.HR., L.ZH., G.XY., C.TM.; Formal Analysis, Z.S.,Y.DL.,L.LX., W.JB., L.Y.; Investigation, G.HR., L.SH L.LX., W.JB.; Writing - Original Draft, G.HR., Z.SG.; Writing - Review & Editing, Z.HR., L.LX., W.JB., L.Y.; Visualization, G.HR., L.YF., S.LM., J.CY., L.XY., Y.GC.; Funding Acquisition, W.Y., L.Y., W.JB., L.LX.; Resources, L.LX., W.JB., L.Y.; Supervision, L.LX., W.JB., L.Y..

Thank you very much for your time and consideration. Wish you have a nice day!

Sincerely yours,
Lianxin Liu

主　题:	回复：The change of co-authors about CDDIS-21-4379RR	
发件人:	ygc1988@sina.com	2022-6-27 20:45:41
收件人:	liulx <liulx@ustc.edu.cn>	

Dear Professor Lianxin Liu，
 I accept all the author information changes.

Guangchao Yang
----- 原始邮件 -----
发件人：liulx@ustc.edu.cn
收件人：郭泓瑞 <hongrui7456@163.com>, 梁书航 <154652031@qq.com>, wangyan2016md@126.com, zhouwill01@163.com, 尹大龙 <dalong48@163.com>, 张树庚 <shugeng517@163.com>, 王继洲 <wangjizhou1984@hotmail.com>, tianxiadiyi_12345@126.com, 1694769800@qq.com, 413951217 <413951217@qq.com>, lemonzhutou@163.com, cyji@mail.ustc.edu.cn, lidonglps@qq.com, drzhr1026@hotmail.com, ygc1988@sina.com, 1006065333@qq.com, 1241346394 <1241346394@qq.com>, 1254007965@qq.com, 刘尧 <liuyao66@ustc.edu.cn>, jbwang16 <jbwang16@ustc.edu.cn>
主题：The change of co-authors about CDDIS-21-4379RR
日期：2022年06月27日 11点48分

Dear Authors：

Our recent manuscript CDDIS-21-4379RR, entitled "Cytochrome B5 Type A alleviates HCC metastasis via regulating STOML2 related autophagy and promoting sensitivity to Ruxolitinib." has been provisionally accepted for publication in Cell Death & Disease, lists you as a co-author. The author information has changed since the original submission.

In particular, I added Jizhou Wang as a co-author in view of his important contribution to the revision process. And I adjusted the order considering the contributions of all authors.

As the author information has changed, I ask for your opinion:

If you **agree** with all the final author information, please reply **I accept all the author information changes.**

If you **disagree** with the final author information or have other suggestions, please email me with details.

The changes of Author List and Contributions are as follows:

Final Author List:

Hongrui Guo[1*], Shuhang Liang[2*], Yan Wang[3*], Shuo Zhou[2*], Dalong Yin[2*], Shugeng Zhang[2], Jizhou Wang[2], Dehai Wu[1], Kun Ma[1], Yufeng Liu[1], Linmao Sun[1], Changyong Ji[2], Xianying Li[2], Huanran Zhou[2], Guangchao Yang[1], Xinyu Guo[1], Tianming Cui[2], Zihao Li[1], Yao Liu[2+], Jiabei Wang[2+], Lianxin Liu[1,4+]

Final Author affiliations:

1.Department of Hepatic Surgery, Key Laboratory of Hepatosplenic Surgery, Ministry of Education, The First Affiliated Hospital of Harbin Medical University, Harbin, China

2.Department of Hepatobiliary Surgery, Anhui Province Key Laboratory of Hepatopancreatobiliary Surgery, The First Affiliated Hospital of USTC, Division of Life Sciences and Medicine, University of

Science and Technology of China, Hefei, Anhui, 230001, China

3.Department of Colorectal Surgery, Harbin Medical University Cancer Hospital, Harbin, 150001 Heilongjiang, China

4. Lead contact

* These authors contributed equally to this work.

Final Author contributions:

Conceptualization, G.HR., L.SH., L.LX., W.JB., L.Y.; Methodology, Y.DL., W.DH., W.Y., Z.S., W.JZ., Z.SG., M.K., L.ZH.; Software, L.YF., S.LM., J.CY., L.XY., Y.GC.; Validation, G.HR., L.SH, Z.HR., L.ZH., G.XY., C.TM.; Formal Analysis, Z.S.,Y.DL.,L.LX., W.JB., L.Y.; Investigation, G.HR., L.SH, L.LX., W.JB.; Writing - Original Draft, G.HR., Z.SG.; Writing - Review & Editing, Z.HR., L.LX., W.JB., L.Y.; Visualization, G.HR., L.YF., S.LM., J.CY., L.XY., Y.GC.; Funding Acquisition, W.Y., L.Y., W.JB., L.LX.; Resources, L.LX., W.JB., L.Y.; Supervision, L.LX., W.JB., L.Y..

Thank you very much for your time and consideration. Wish you have a nice day!

Sincerely yours，

Lianxin Liu

主　题:	回复: The change of co-authors about CDDIS-21-4379RR	
发件人:	"郭新宇" <1006065333@qq.com>	2022-6-27 13:05:33
收件人:	liulx <liulx@ustc.edu.cn>	

Dear Professor Liu:

I accept all the author information changes.

Best wishes.
Guo Xinyu

------------------ 原始邮件 ------------------

发件人: "liulx" <liulx@ustc.edu.cn>;
发送时间: 2022年6月27日(星期一) 中午11:48
收件人: "郭泓瑞"<hongrui7456@163.com>;"梁书航"<154652031@qq.com>;"wangyan2016md" <wangyan2016md@126.com>;"zhouwill01"<zhouwill01@163.com>;"尹大龙"<dalong48@163.com>;"张树庚" <shugeng517@163.com>;"王继洲"<wangjizhou1984@hotmail.com>;"tianxiadiyi_12345" <tianxiadiyi_12345@126.com>;"1694769800"<1694769800@qq.com>;"413951217"<413951217@qq.com>;"lemonzhutou" <lemonzhutou@163.com>;"cyji"<cyji@mail.ustc.edu.cn>;"lidonglps"<lidonglps@qq.com>;"drzhr1026" <drzhr1026@hotmail.com>;"ygc1988"<ygc1988@sina.com>;"郭新宇"<1006065333@qq.com>;"1241346394" <1241346394@qq.com>;"1254007965"<1254007965@qq.com>;"刘尧"<liuyao66@ustc.edu.cn>;"jbwang16" <jbwang16@ustc.edu.cn>;
主题: The change of co-authors about CDDIS-21-4379RR

Dear Authors:

Our recent manuscript CDDIS-21-4379RR, entitled "Cytochrome B5 Type A alleviates HCC metastasis via regulating STOML2 related autophagy and promoting sensitivity to Ruxolitinib." has been provisionally accepted for publication in Cell Death & Disease, lists you as a co-author. The author information has changed since the original submission.

In particular, I added Jizhou Wang as a co-author in view of his important contribution to the revision process. And I adjusted the order considering the contributions of all authors.

As the author information has changed, I ask for your opinion:

If you **agree** with all the final author information, please reply **I accept all the author information changes.**

If you **disagree** with the final author information or have other suggestions, please email me with details.

The changes of Author List and Contributions are as follows:

Final Author List:

Hongrui Guo[1]*, Shuhang Liang[2]*, Yan Wang[3]*, Shuo Zhou[2]*, Dalong Yin[2]*, Shugeng Zhang[2], Jizhou Wang[2], Dehai Wu[1], Kun Ma[1], Yufeng Liu[1], Linmao Sun[1], Changyong Ji[2], Xianying Li[2],

Huanran Zhou[2], Guangchao Yang[1], Xinyu Guo[1], Tianming Cui[2], Zihao Li[1], Yao Liu[2+], Jiabei Wang[2+], Lianxin Liu[1,4+]

Final Author affiliations:

1.Department of Hepatic Surgery, Key Laboratory of Hepatosplenic Surgery, Ministry of Education, The First Affiliated Hospital of Harbin Medical University, Harbin, China

2.Department of Hepatobiliary Surgery, Anhui Province Key Laboratory of Hepatopancreatobiliary Surgery, The First Affiliated Hospital of USTC, Division of Life Sciences and Medicine, University of Science and Technology of China, Hefei, Anhui, 230001, China

3.Department of Colorectal Surgery, Harbin Medical University Cancer Hospital, Harbin, 150001 Heilongjiang, China

4. Lead contact

* These authors contributed equally to this work.

Final Author contributions:

Conceptualization, G.HR., L.SH., L.LX., W.JB., L.Y.; Methodology, Y.DL., W.DH., W.Y., Z.S., W.JZ., Z.SG., M.K., L.ZH.; Software, L.YF., S.LM., J.CY., L.XY., Y.GC.; Validation, G.HR., L.SH, Z.HR., L.ZH., G.XY., C.TM.; Formal Analysis, Z.S.,Y.DL.,L.LX., W.JB., L.Y.; Investigation, G.HR., L.SH, L.LX., W.JB.; Writing - Original Draft, G.HR., Z.SG.; Writing - Review & Editing, Z.HR., L.LX., W.JB., L.Y.; Visualization, G.HR., L.YF., S.LM., J.CY., L.XY., Y.GC.; Funding Acquisition, W.Y., L.Y., W.JB., L.LX.; Resources, L.LX., W.JB., L.Y.; Supervision, L.LX., W.JB., L.Y..

Thank you very much for your time and consideration. Wish you have a nice day!

Sincerely yours,

Lianxin Liu

主　题:	回复：The change of co-authors about CDDIS-21-4379RR	
发件人:	"传说中的小铭" <1241346394@qq.com>	2022-6-27 13:17:41
收件人:	liulx <liulx@ustc.edu.cn>	

Dear Professor Lianxin Liu，
I accept all the author information changes.

Tianming Cui

------------------ 原始邮件 ------------------

发件人: "liulx" <liulx@ustc.edu.cn>;
发送时间: 2022年6月27日(星期一) 中午11:48
收件人: "郭泓瑞"<hongrui7456@163.com>;"梁书航"<154652031@qq.com>;"wangyan2016md"<wangyan2016md@126.com>;"zhouwill01"<zhouwill01@163.com>;"尹大龙"<dalong48@163.com>;"张树庚"<shugeng517@163.com>;"王继洲"<wangjizhou1984@hotmail.com>;"tianxiadiyi_12345"<tianxiadiyi_12345@126.com>;"1694769800"<1694769800@qq.com>;"413951217"<413951217@qq.com>;"lemonzhutou"<lemonzhutou@163.com>;"cyji"<cyji@mail.ustc.edu.cn>;"lidonglps"<lidonglps@qq.com>;"drzhr1026"<drzhr1026@hotmail.com>;"ygc1988"<ygc1988@sina.com>;"1006065333"<1006065333@qq.com>;"传说中的小铭"<1241346394@qq.com>;"1254007965"<1254007965@qq.com>;"刘尧"<liuyao66@ustc.edu.cn>;"jbwang16"<jbwang16@ustc.edu.cn>;
主题: The change of co-authors about CDDIS-21-4379RR

Dear Authors：

Our recent manuscript CDDIS-21-4379RR, entitled "Cytochrome B5 Type A alleviates HCC metastasis via regulating STOML2 related autophagy and promoting sensitivity to Ruxolitinib." has been provisionally accepted for publication in Cell Death & Disease, lists you as a co-author. The author information has changed since the original submission.

In particular, I added Jizhou Wang as a co-author in view of his important contribution to the revision process. And I adjusted the order considering the contributions of all authors.

As the author information has changed, I ask for your opinion:

If you **agree** with all the final author information, please reply **I accept all the author information changes.**

If you **disagree** with the final author information or have other suggestions, please email me with details.

The changes of Author List and Contributions are as follows:

Final Author List:

Hongrui Guo[1*], Shuhang Liang[2*], Yan Wang[3*], Shuo Zhou[2*], Dalong Yin[2*], Shugeng Zhang[2], Jizhou Wang[2], Dehai Wu[1], Kun Ma[1], Yufeng Liu[1], Linmao Sun[1], Changyong Ji[2], Xianying Li[2], Huanran Zhou[2], Guangchao Yang[1], Xinyu Guo[1], Tianming Cui[2], Zihao Li[1], Yao Liu[2+], Jiabei Wang[2+], Lianxin Liu[1,4+]

Final Author affiliations:

1.Department of Hepatic Surgery, Key Laboratory of Hepatosplenic Surgery, Ministry of Education, The First Affiliated Hospital of Harbin Medical University, Harbin, China

2.Department of Hepatobiliary Surgery, Anhui Province Key Laboratory of Hepatopancreatobiliary Surgery, The First Affiliated Hospital of USTC, Division of Life Sciences and Medicine, University of Science and Technology of China, Hefei, Anhui, 230001, China

3.Department of Colorectal Surgery, Harbin Medical University Cancer Hospital, Harbin, 150001 Heilongjiang, China

4. Lead contact

* These authors contributed equally to this work.

Final Author contributions:

Conceptualization, G.HR., L.SH., L.LX., W.JB., L.Y.; Methodology, Y.DL., W.DH., W.Y., Z.S., W.JZ., Z.SG., M.K., L.ZH.; Software, L.YF., S.LM., J.CY., L.XY., Y.GC.; Validation, G.HR., L.SH, Z.HR., L.ZH., G.XY., C.TM.; Formal Analysis, Z.S.,Y.DL.,L.LX., W.JB., L.Y.; Investigation, G.HR., L.SH, L.LX., W.JB.; Writing - Original Draft, G.HR., Z.SG.; Writing - Review & Editing, Z.HR., L.LX., W.JB., L.Y.; Visualization, G.HR., L.YF., S.LM., J.CY., L.XY., Y.GC.; Funding Acquisition, W.Y., L.Y., W.JB., L.LX.; Resources, L.LX., W.JB., L.Y.; Supervision, L.LX., W.JB., L.Y..

Thank you very much for your time and consideration. Wish you have a nice day!

Sincerely yours,

Lianxin Liu

主　题:	The change of co-authors about CDDIS-21-4379RR	
发件人:	"李梓豪" <1254007965@qq.com>	2022-6-27 13:16:27
收件人:	liulx <liulx@ustc.edu.cn>	

Dear Professor Lianxin Liu，　I accept all the author information changes. zihao Li

---原始邮件---

发件人："liulx"

发送时间："2022/6/27 11:48:21"

收件人："郭泓瑞","梁书航"<154652031@qq.com>,"wangyan2016md","zhouwill01","尹大龙","张树庚","王继洲","tianxiadiyi_12345","1694769800"<1694769800@qq.com>,"413951217"<413951217@qq.com>,"lemonzhutou","cyji","lidonglps","drzhr1026","ygc1988","1006065333"<1006065333@qq.com>,"1241346394"<1241346394@qq.com>,"1254007965"<1254007965@qq.com>,"刘尧","jbwang16"

主题：The change of co-authors about CDDIS-21-4379RR

Dear Authors：

Our recent manuscript CDDIS-21-4379RR, entitled "Cytochrome B5 Type A alleviates HCC metastasis via regulating STOML2 related autophagy and promoting sensitivity to Ruxolitinib." has been provisionally accepted for publication in Cell Death & Disease, lists you as a co-author. The author information has changed since the original submission.

In particular, I added Jizhou Wang as a co-author in view of his important contribution to the revision process. And I adjusted the order considering the contributions of all authors.

As the author information has changed, I ask for your opinion:

If you **agree** with all the final author information, please reply **I accept all the author information changes.**

If you **disagree** with the final author information or have other suggestions, please email me with details.

The changes of Author List and Contributions are as follows:

Final Author List:

Hongrui Guo[1*], Shuhang Liang[2*], Yan Wang[3*], Shuo Zhou[2*], Dalong Yin[2*], Shugeng Zhang[2], Jizhou Wang[2], Dehai Wu[1], Kun Ma[1], Yufeng Liu[1], Linmao Sun[1], Changyong Ji[2], Xianying Li[2], Huanran Zhou[2], Guangchao Yang[1], Xinyu Guo[1], Tianming Cui[2], Zihao Li[1], Yao Liu[2+], Jiabei Wang[2+], Lianxin Liu[1,4+]

Final Author affiliations:

1.Department of Hepatic Surgery, Key Laboratory of Hepatosplenic Surgery, Ministry of Education, The First Affiliated Hospital of Harbin Medical University, Harbin, China

2.Department of Hepatobiliary Surgery, Anhui Province Key Laboratory of Hepatopancreatobiliary Surgery, The First Affiliated Hospital of USTC, Division of Life Sciences and Medicine, University of Science and Technology of China, Hefei, Anhui, 230001, China

3.Department of Colorectal Surgery, Harbin Medical University Cancer Hospital, Harbin, 150001 Heilongjiang, China

4. Lead contact

* These authors contributed equally to this work.

Final Author contributions:

Conceptualization, G.HR., L.SH., L.LX., W.JB., L.Y.; Methodology, Y.DL., W.DH., W.Y., Z.S., W.JZ., Z.SG., M.K., L.ZH.; Software, L.YF., S.LM., J.CY., L.XY., Y.GC.; Validation, G.HR., L.SH, Z.HR., L.ZH., G.XY., C.TM.; Formal Analysis, Z.S.,Y.DL.,L.LX., W.JB., L.Y.; Investigation, G.HR., L.SH, L.LX., W.JB.; Writing - Original Draft, G.HR., Z.SG.; Writing - Review & Editing, Z.HR., L.LX., W.JB., L.Y.; Visualization, G.HR., L.YF., S.LM., J.CY., L.XY., Y.GC.; Funding Acquisition, W.Y., L.Y., W.JB., L.LX.; Resources, L.LX., W.JB., L.Y.; Supervision, L.LX., W.JB., L.Y..

Thank you very much for your time and consideration. Wish you have a nice day!

Sincerely yours,

Lianxin Liu

主　题:	Re: The change of co-authors about CDDIS-21-4379RR
发件人:	"刘尧" <liuyao66@ustc.edu.cn> 2022-6-27 12:38:32
收件人:	liulx@ustc.edu.cn

Dear Prof. Lianxin Liu,

Congratulations! I accept all the author information changes.

Yao Liu

-----原始邮件-----
发件人:liulx@ustc.edu.cn
发送时间:2022-06-27 11:48:19 (星期一)
收件人: "郭泓瑞" <hongrui7456@163.com>, "梁书航" <154652031@qq.com>, wangyan2016md@126.com, zhouwill01@163.com, "尹大龙" <dalong48@163.com>, "张树庚" <shugeng517@163.com>, "王继洲" <wangjizhou1984@hotmail.com>, tianxiadiyi_12345@126.com, 1694769800@qq.com, 413951217 <413951217@qq.com>, lemonzhutou@163.com, cyji@mail.ustc.edu.cn, lidonglps@qq.com, drzhr1026@hotmail.com, ygc1988@sina.com, 1006065333@qq.com, 1241346394 <1241346394@qq.com>, 1254007965@qq.com, "刘尧" <liuyao66@ustc.edu.cn>, jbwang16 <jbwang16@ustc.edu.cn>
抄送:
主题: The change of co-authors about CDDIS-21-4379RR

Dear Authors：

Our recent manuscript CDDIS-21-4379RR, entitled "Cytochrome B5 Type A alleviates HCC metastasis via regulating STOML2 related autophagy and promoting sensitivity to Ruxolitinib." has been provisionally accepted for publication in Cell Death & Disease, lists you as a co-author. The author information has changed since the original submission.

In particular, I added Jizhou Wang as a co-author in view of his important contribution to the revision process. And I adjusted the order considering the contributions of all authors.

As the author information has changed, I ask for your opinion:

If you **agree** with all the final author information, please reply **I accept all the author information changes.**

If you **disagree** with the final author information or have other suggestions, please email me with details.

The changes of Author List and Contributions are as follows:

Final Author List:

Hongrui Guo[1*], Shuhang Liang[2*], Yan Wang[3*], Shuo Zhou[2*], Dalong Yin[2*], Shugeng Zhang[2], Jizhou Wang[2], Dehai Wu[1], Kun Ma[1], Yufeng Liu[1], Linmao Sun[1], Changyong Ji[2], Xianying Li[2], Huanran Zhou[2], Guangchao Yang[1], Xinyu Guo[1], Tianming Cui[2], Zihao Li[1], Yao Liu[2+], Jiabei Wang[2+], Lianxin Liu[1,4+]

Final Author affiliations:

1.Department of Hepatic Surgery, Key Laboratory of Hepatosplenic Surgery, Ministry of Education, The First Affiliated Hospital of Harbin Medical University, Harbin, China

2.Department of Hepatobiliary Surgery, Anhui Province Key Laboratory of Hepatopancreatobiliary Surgery, The First Affiliated Hospital of USTC, Division of Life Sciences and Medicine, University of Science and Technology of China, Hefei, Anhui, 230001, China

3.Department of Colorectal Surgery, Harbin Medical University Cancer Hospital, Harbin, 150001 Heilongjiang, China

4. Lead contact

* These authors contributed equally to this work.

Final Author contributions:

Conceptualization, G.HR., L.SH., L.LX., W.JB., L.Y.; Methodology, Y.DL., W.DH., W.Y., Z.S., W.JZ., Z.SG., M.K., L.ZH.; Software, L.YF., S.LM., J.CY., L.XY., Y.GC.; Validation, G.HR., L.SH Z.HR., L.ZH., G.XY., C.TM.; Formal Analysis, Z.S.,Y.DL.,L.LX., W.JB., L.Y.; Investigation, G.HR., L.SH, L.LX., W.JB.; Writing - Original Draft, G.HR., Z.SG.; Writing - Review & Editing, Z.HR., L.LX., W.JB., L.Y.; Visualization, G.HR., L.YF., S.LM., J.CY., L.XY., Y.GC.; Funding Acquisition, W.Y., L.Y., W.JB., L.LX.; Resources, L.LX., W.JB., L.Y.; Supervision, L.LX., W.JB., L.Y..

Thank you very much for your time and consideration. Wish you have a nice day!

Sincerely yours，

Lianxin Liu

主　题:	Re: The change of co-authors about CDDIS-21-4379RR	
发件人:	jbwang16@ustc.edu.cn	2022-6-27 15:24:50
收件人:	liulx@ustc.edu.cn	

I accept all the author information changes.

Jiabei Wang

-----原始邮件-----
发件人:liulx@ustc.edu.cn
发送时间:2022-06-27 11:48:19 (星期一)
收件人: "郭泓瑞" <hongrui7456@163.com>, "梁书航" <154652031@qq.com>, wangyan2016md@126.com, zhouwill01@163.com, "尹大龙" <dalong48@163.com>, "张树庚" <shugeng517@163.com>, "王继洲" <wangjizhou1984@hotmail.com>, tianxiadiyi_12345@126.com, 1694769800@qq.com, 413951217 <413951217@qq.com>, lemonzhutou@163.com, cyji@mail.ustc.edu.cn, lidonglps@qq.com, drzhr1026@hotmail.com, ygc1988@sina.com, 1006065333@qq.com, 1241346394 <1241346394@qq.com>, 1254007965@qq.com, "刘尧" <liuyao66@ustc.edu.cn>, jbwang16 <jbwang16@ustc.edu.cn>
抄送:
主题: The change of co-authors about CDDIS-21-4379RR

Dear Authors:

Our recent manuscript CDDIS-21-4379RR, entitled "Cytochrome B5 Type A alleviates HCC metastasis via regulating STOML2 related autophagy and promoting sensitivity to Ruxolitinib." has been provisionally accepted for publication in Cell Death & Disease, lists you as a co-author. The author information has changed since the original submission.

In particular, I added Jizhou Wang as a co-author in view of his important contribution to the revision process. And I adjusted the order considering the contributions of all authors.

As the author information has changed, I ask for your opinion:

If you **agree** with all the final author information, please reply **I accept all the author information changes.**

If you **disagree** with the final author information or have other suggestions, please email me with details.

The changes of Author List and Contributions are as follows:

Final Author List:

Hongrui Guo[1]*, Shuhang Liang[2]*, Yan Wang[3]*, Shuo Zhou[2]*, Dalong Yin[2]*, Shugeng Zhang[2], Jizhou Wang[2], Dehai Wu[1], Kun Ma[1], Yufeng Liu[1], Linmao Sun[1], Changyong Ji[2], Xianying Li[2], Huanran Zhou[2], Guangchao Yang[1], Xinyu Guo[1], Tianming Cui[2], Zihao Li[1], Yao Liu[2+], Jiabei Wang[2+], Lianxin Liu[1,4+]

Final Author affiliations:

1.Department of Hepatic Surgery, Key Laboratory of Hepatosplenic Surgery, Ministry of Education, The First Affiliated Hospital of Harbin Medical University, Harbin, China

2.Department of Hepatobiliary Surgery, Anhui Province Key Laboratory of Hepatopancreatobiliary Surgery, The First Affiliated Hospital of USTC, Division of Life Sciences and Medicine, University of Science and Technology of China, Hefei, Anhui, 230001, China

3.Department of Colorectal Surgery, Harbin Medical University Cancer Hospital, Harbin, 150001 Heilongjiang, China

4. Lead contact

* These authors contributed equally to this work.

Final Author contributions:

Conceptualization, G.HR., L.SH., L.LX., W.JB., L.Y.; Methodology, Y.DL., W.DH., W.Y., Z.S., W.JZ., Z.SG., M.K., L.ZH.; Software, L.YF., S.LM., J.CY., L.XY., Y.GC.; Validation, G.HR., L.SH, Z.HR., L.ZH., G.XY., C.TM.; Formal Analysis, Z.S.,Y.DL.,L.LX., W.JB., L.Y.; Investigation, G.HR., L.SH, L.LX., W.JB.; Writing - Original Draft, G.HR., Z.SG.; Writing - Review & Editing, Z.HR., L.LX., W.JB., L.Y.; Visualization, G.HR., L.YF., S.LM., J.CY., L.XY., Y.GC.; Funding Acquisition, W.Y., L.Y., W.JB., L.LX.; Resources, L.LX., W.JB., L.Y.; Supervision, L.LX., W.JB., L.Y..

Thank you very much for your time and consideration. Wish you have a nice day!

Sincerely yours，

Lianxin Liu
